# Phyllofenones F–M, Scalarane Sesterterpenes from the Marine Sponge *Phyllospongia foliascens*

**DOI:** 10.3390/md21100507

**Published:** 2023-09-26

**Authors:** Hao-Bing Yu, Bo Hu, Zhe Ning, Ying He, Xiao-Ling Men, Zi-Fei Yin, Bing-Hua Jiao, Xiao-Yu Liu, Hou-Wen Lin

**Affiliations:** 1 Department of Marine Biomedicine and Polar Medicine, Naval Medical Center of PLA, Naval Medical University, Shanghai 200433, China; yuhaobing1986@126.com (H.-B.Y.); hb8601@163.com (B.H.); ningzhe95@163.com (Z.N.); yinghe_hys@163.com (Y.H.); xlmen2021@163.com (X.-L.M.); yinzifei870730smmu@163.com (Z.-F.Y.); jiaobh@live.cn (B.-H.J.); 2 Research Center for Marine Drugs, State Key Laboratory of Oncogenes and Related Genes, School of Medicine, Shanghai Jiao Tong University, Shanghai 200127, China; 3 School of Traditional Chinese Medicine, Naval Medical University, Shanghai 200433, China; 4 Department of Biochemistry and Molecular Biology, College of Basic Medical Sciences, Naval Medical University, Shanghai 200433, China

**Keywords:** scalarane, *Phyllospongia foliascens*, cytotoxicity, antibacterial activity

## Abstract

Eight new scalarane sesterterpenes, phyllofenones F–M (**1**–**8**), together with two known analogues, carteriofenones B and A (**9**–**10**), were isolated from the marine sponge *Phyllospongia foliascens* collected from the South China Sea. The structures of these compounds were determined based on extensive spectroscopic and quantum chemical calculation analysis. The antibacterial and cytotoxic activity of these compounds was evaluated. Among them, only compounds **4** and **6** displayed weak inhibitory activity against *Staphylococcus aureus* and *Escherichia coli*, with MIC values of 16 μg/mL and 8 μg/mL, respectively. Compounds **1**–**10** exhibited cytotoxic activity against the HeLa, HCT-116, H460, and SW1990 cancer cell lines, with IC_50_ values ranging from 3.4 to 19.8 μM.

## 1. Introduction

Marine organisms have gained increasing attention as potential sources of interesting secondary metabolites with broad-spectrum activity and novel chemical structures [1]. Scalaranes, a family of bioactive marine sesterterpenoids, possess a 6/6/6/6 tetracyclic or 6/6/6/6/5 pentacyclic fused ring system [2]. Scalaranes can be divided into homoscalaranes (methylated at C-20 or C-24) and bishomoscalaranes (methylated at both C-20 and C-24) [2,3]. They are exclusively obtained from nudibranchs and their food chain, marine sponges [3,4]. The majority (around 90%) of scalaranes have been isolated from various marine sponges, including *Hyrtios* sp., *Phyllospongia* (previously identified as *Carteriospongia*) sp., *Dysidea* sp., *Lendenfeldia* sp., *hippospongia* sp., *Scalarispongia* sp., *Spongia* sp., *Psammocinia* sp., *Ircinia* sp., *Euryspongia* sp., *Hyatella* sp., *Hyattlla* sp., *Coscinoderma* sp., *Smenospongia* sp., and *Collospongia* sp. [2,4]. Since the first scalarane, scalarin, was reported in 1972 [5], approximately 500 scalaranes have been identified [2,3]. Moreover, extensive research has been conducted on the synthesis or semi-synthesis of scalarane derivatives due to their diverse bioactivity, including cytotoxic [6], anti-inflammatory [7], antimicrobial [4,8], and enzyme-inhibitory activity [9].

**Figure 1:**
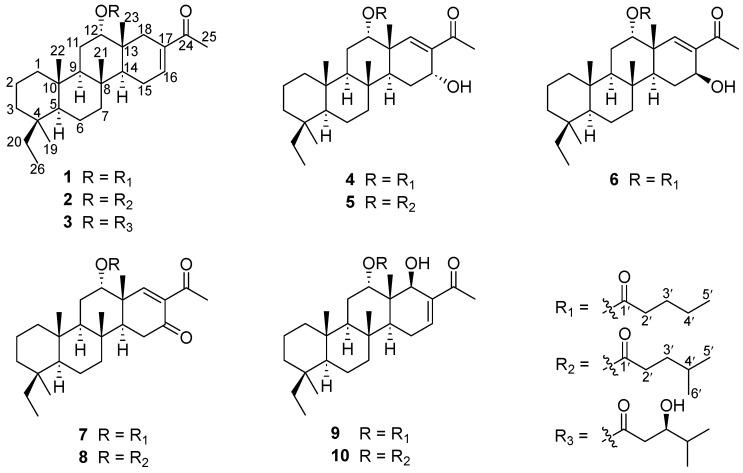
Structures of the isolated compounds **1**–**10**.

During our ongoing research on bioactive secondary metabolites from marine sponges in the South China Sea [4,7,10], we discovered that an extract obtained from the sponge *P. foliascens* demonstrated potent cytotoxic activity against human cancer cells. Following a comprehensive chemical investigation of the bioactive extracts, we successfully isolated eight new scalaranes, phyllofenones F–M (**1**–**8**), along with two known analogues, carteriofenones B and A (**9**–**10**) (Figure 1). Herein, we report the isolation, structure elucidation, and bioactivity of these scalaranes.

## 2. Results

**Figure 2:**
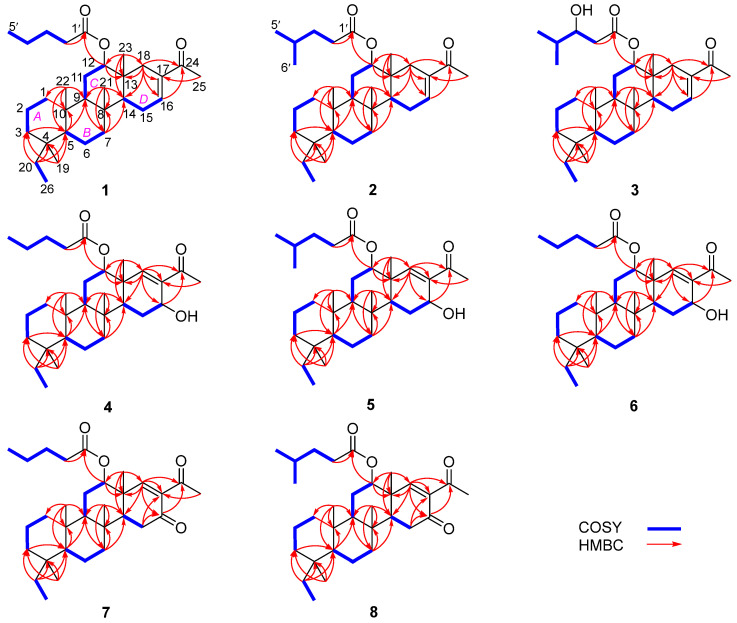
Key COSY and HMBC correlations of **1**–**8**.

**Figure 3:**
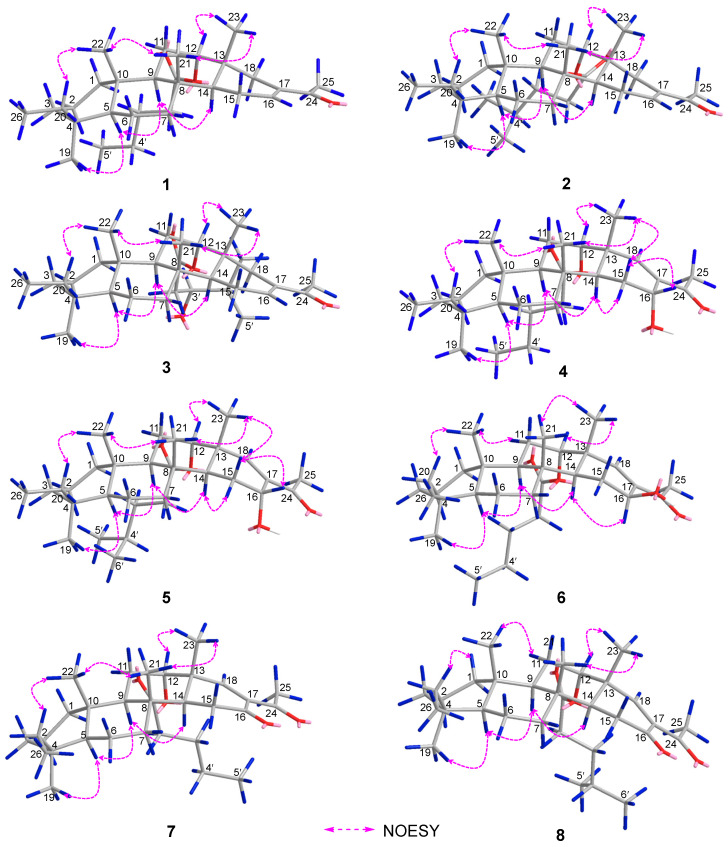
Key NOESY correlations of **1**–**8**.

**Figure 4:**
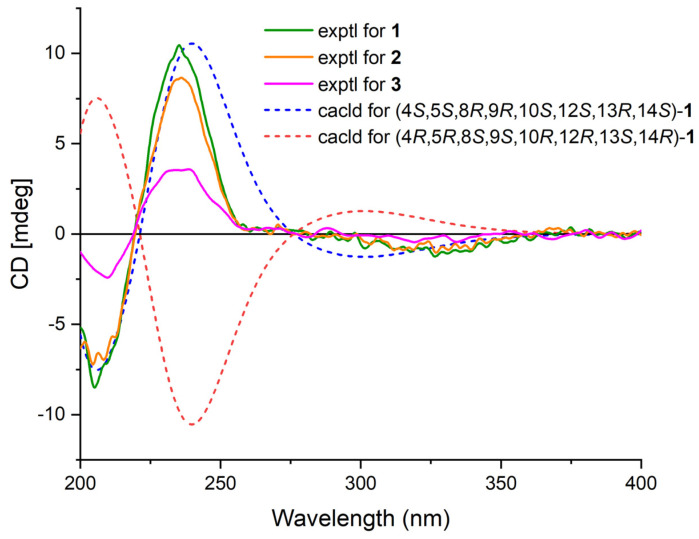
Experimental ECD spectra of **1**–**3** and calculated ECD spectra of **1**.

Phyllofenone F (**1**) was obtained as a white powder. Its molecular formula was determined to be C_31_H_50_O_3_ based on the molecular ion peak at [M + Na]^+^ *m*/*z* 493.3652, indicating the presence of seven degrees of unsaturation. The existence of a carbonyl group was confirmed by the IR absorption at 1723 cm^–1^ [4]. In the ^1^H NMR spectrum of **1** (Table 1), five methyl singlets were observed at δ_H_ 0.80, 0.83, 0.85, 0.92, and 2.28, along with two methyl triplets at δ_H_ 0.74 (t, 7.5) and 0.92 (t, 7.5) and one olefinic methine multiplet at δ_H_ 6.85. The ^13^C NMR and 135 DEPT spectra indicated the presence of seven methyl groups (including one ketone methyl at δ_C_ 25.3), 12 methylene groups, five methine groups (including one oxygenated at δ_C_ 76.7 and one olefinic at δ_C_ 139.5), and seven quaternary carbons (including one olefinic at δ_C_ 137.7, one ester carbonyl δ_C_ 172.9, and one ketone carbonyl at δ_C_ 199.0). The NMR data accounted for three degrees of unsaturation, suggesting a tetracyclic core structure in **1**. The analysis of the 1D NMR data revealed that the C26 20,24-bishomo-25-norscalarane sesterterpene skeleton of **1** was closely related to the known compound phyllofenone A (Figure S85) [11]. The HMBC correlations (Figure 2) from H_3_-19 and H_2_-20 to C-3, C-4, and C-5, from H_3_-21 to C-7, C-8, C-9, and C-14, from H_3_-22 to C-1, C-5, C-9, and C-10, from H_3_-23 to C-12, C-13, C-14, and C-18, and from H-18α and H-18β to C-16 and C-17, together with the COSY correlations (Figure 2) of H-1α/H-2α/H-3α, H-5/H_2_-6/H_2_-7, H-9/H_2_-11/H-12, and H-14/H_2_-15/H-16, provided further evidence of the presence of an A/B/C/D ring scalarane system in **1** [11,12]. The COSY correlations from H_2_-2′ to H_2_-3′, H_2_-3′/H_2_-4′, and H_2_-4′/H_3_-5′ and HMBC correlations from H-12 and H_2_-2′ to C-1′ suggested that a valerate group was connected to ring C via the downfield shift carbon C-12 (δ_C_ 76.7). Additionally, HMBC correlations from H_3_-25 (δ_H_ 2.28) to C-17 and C-24 (δ_C_ 199.0) indicated the presence of an acetyl group at C-17. Therefore, the planar structure of **1** was established as a 6/6/6/6 tetracyclic scalarane sesterterpene. The relative configuration of **1** was deduced from a NOESY experiment. The NOESY correlations between H_2_-20/H_3_-22, H_3_-21/H_3_-22, H-12/H_3_-23, and H_3_-21/H_3_-23 indicated their β-orientation, whereas the NOESY correlations between H-5/H_3_-19, H-5/H-9, and H-9/H-14 suggested their α-orientation and therefore that all junctures for rings were *trans* A/B/C/D (Figure 3). Furthermore, the β-orientation of H_2_-19 and H-12 was also deduced from the ^13^C NMR chemical shift of CH_2_-20 (δ_C_ 24.5) and the small *J*-value (*J* = 2.5 Hz) observed for H-12 (δ_H_ 4.79), respectively [4,11]. Therefore, the relative configuration of **1** was determined. The CD spectrum of **1** exhibited a characteristic positive Cotton effect at 237 nm, and its specific rotation (${\left[\alpha \right]}_{D}^{25}$ +73.5, *c* 0.1, MeCN) was almost identical to that of phyllofenone A (Figure S85) (CD 237 nm +6.2 MeCN; ${\left[\alpha \right]}_{D}$ +8.0, *c* 0.3, MeOH) [11]. These findings indicated that **1** possessed the same absolute configuration as phyllofenone A, 4*S*, 5*S*, 8*R*, 9*R*, 10*S*, 12*S*, 13*R*, 14*S*, which was also supported by the comparison of the experimental and calculated ECD spectra (Figure 4).

Phyllofenone G (**2**) was also isolated as a white powder. Its molecular formula was determined to be C_32_H_52_O_3_, with 11 degrees of unsaturation, based on the HRESIMS ion at *m*/*z* 507.3815 [M + Na]^+^. A thorough analysis of the 1D NMR data (Table 1) of **2** indicated that it belonged to the same class of scalarane skeletons as **1**. However, notable differences between **1** and **2** were observed. Compound **2** exhibited additional methyl and methine signals, while one methylene resonance was absent, indicating that C-12 in **2** was substituted by a 4-methylpentanoate subunit. This was confirmed by the COSY correlations of H_2_-2′ (δ_H_ 2.32)/H_2_-3′ (δ_H_ 1.54), H_2_-3′/H-4′ (δ_H_ 1.60), H-4′/H_3_-5′ (δ_H_ 0.90), and H-4′/H_3_-6′ (δ_H_ 0.90), as well as the HMBC correlations from H_2_-2′ and H-12 (δ_H_ 4.80) to C-1′ (δ_C_ 173.1) (Figure 2). The similarity observed in the NOESY correlations (Figure 3), the coupling constant of H-12, and the CD spectra (Figure 4) between **1** and **2** indicated that **2** possessed the same absolute configuration as **1** [4,11].

Phyllofenone H (**3**), isolated as a white powder, showed a molecular formula of C_32_H_52_O_4_ based on HRESIMS, which is larger than that of **2** by 16 amu. The NMR data (Table 1) of **2** were almost identical to those of **3**, supporting the presence of the same scalarane core. However, an obvious difference was noted, with **3** containing a 3-OH-4-methylpentanoate subunit instead of the 4-methylpentanoate subunit found in **2**. The presence of the 3-OH-4-methylpentanoate group was confirmed by the COSY correlations of H_2_-2′a (δ_H_ 2.41)/H_2_-3′ (δ_H_ 3.79), H_2_-3′/H-4′ (δ_H_ 1.71), H-4′/H_3_-5′ (δ_H_ 0.97), and H-4′/H_3_-6′ (δ_H_ 0.93) and HMBC correlations from H_2_-2′a and H-12 (δ_H_ 4.85) to C-1′ (δ_C_ 172.9) (Figure 2), along the molecular formula and ^13^C NMR chemical shift of C-3′ (δ_C_ 72.7). The relative configuration of **3** was identical to that of **2** in the comparison of their chemical shifts, the coupling constant of H-12, NOESY correlations (Figure 3), and CD spectra (Figure 4) [4,11]. The 3′*R* configuration was determined by comparing the specific rotations of (*S*)-3-hydroxy-4-methylpentanoic acid (${\left[\alpha \right]}_{D}$ −25.3, CHCl_3_, *c* 1.2) [13] with (*R*)-3-hydroxy-4-methylpentanoic acid (${\left[\alpha \right]}_{D}$ +39.8, CHCl_3_, *c* 1.0) [14,15] and those of **3** (${\left[\alpha \right]}_{D}^{25}$ +78.1, MeCN, *c* 0.1) with **2** (${\left[\alpha \right]}_{D}^{25}$ +60.2, MeCN, *c* 0.1), respectively. Additionally, the *R* configuration for C-3′ was also confirmed by the specific rotation of the 3-hydroxy-4-methylpentanoic acid subunit (${\left[\alpha \right]}_{D}^{25}$ +2.1, CHCl_3_, *c* 0.1), which was derived from the hydrolysis of **3** and subsequent purification by small-scale column chromatography on silica gel. The absolute configuration of **3** was determined as 4*S*, 5*S*, 8*R*, 9*R*, 10*S*, 12*S*, 13*R*, 14*S*, 3′*R*.

**Figure 5:**
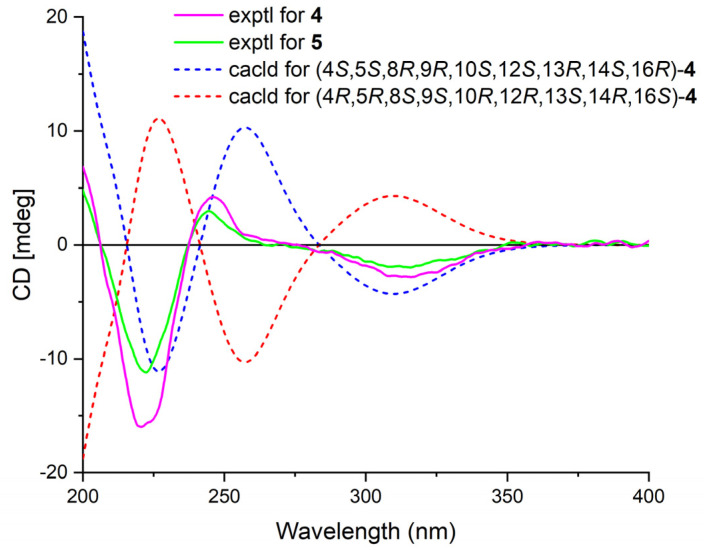
Experimental ECD spectra of **4**–**5** and calculated ECD spectra of **4**.

Phyllofenone I (**4**) was also isolated as a white powder, and its molecular formula was determined as C_31_H_50_O_4_ based on HRESIMS data (Table 2); it was 16 amu larger than that of **1**. The IR spectrum indicated the presence of hydroxy (3535 cm^−1^) and carbonyl (1729 cm^−1^) groups [16]. Careful analysis of the NMR data revealed a 6/6/6/6 fused scalarane sesterterpene for **4**, similar to **1** [16,17]. A notable distinction between these two compounds was the downshift of a single olefinic carbon from δ_C_ 139.5 in **1** to δ_C_ 152.2 in **4**, as well as the presence of an oxygenated methine (δ_C_ 63.3, δ_H_ 4.56) in **4** instead of the methylene (δ_C_ 35.5, δ_H_ 2.21) found in **1**. These differences suggested the existence of a C-17/C-18 double bond in ring D with hydroxy substitution at C-16. Additional HMBC correlations from H-16 to C-17 and C-14, from H-18 to C-13, C-17, and C-24, and from H_3_-23 to C-13, C-14, and C-18, along with consecutive COSY correlations of H-14/H_2_-15/H-16, confirmed this hypothesis (Figure 2). The NOESY correlations of H-5/H-9 and H_3_-19, H-7α/H-9 and H-14, and H-14/H-15α indicated that these protons were on the same face of the molecule. Other sets of NOESY correlations of H_3_-22/H_3_-21, H-20β, and H_3_-23, H_3_-23/H-12 and H-15β, and H-15β/H-16 and H_3_-21 indicated that these protons were located on the other face of the molecule (Figure 3). Based on the chemical shifts, the coupling constant of H-12, and the above NOESY data [4,11], the relative configuration of **4** was determined as depicted. Moreover, the quantum chemical electronic circular dichroism (ECD) calculation method was employed to determine the whole absolute configurations of **4**. The calculated ECD spectrum of the 4*S*, 5*S*, 8*R*, 9*R*, 10*S*, 12*S*, 13*R*, 14*S*, 16*R* enantiomer approximately matched the Cotton effects observed in the experimental ECD spectrum of **4** (Figure 5), enabling the assignment of the absolute configuration as shown.

Phyllofenone J (**5**) was obtained as a white powder, and its molecular formula was determined to be C_32_H_52_O_4_ by HRESIMS analysis. The NMR data of **5** (Table 2) were almost identical to those for **4** except for the different substituent at C-12. In compound **5**, a 4-methylpentanoate group was attached to C-12 instead of the valerate group present in **4**. This was supported by the HMBC correlations from H-12 (δ_H_ 5.05) and H_2_-2′ (δ_H_ 1.48) to C-1′ (δ_C_ 173.8), as well as COSY correlations of H_2_-2′/H_2_-3′ (δ_H_ 2.30)/H-4′ (δ_H_ 1.55)/H_3_-5′ (δ_H_ 0.87) and H-4′/H_3_-6′ (δ_H_ 0.88) (Figure 2). Furthermore, the similarity of their NOESY correlations (Figure 3), the coupling constant of H-12, and the CD spectra (Figure 5) between **4** and **5** suggested that compound **5** possessed the same absolute configuration as **4** [4,11].

**Figure 6:**
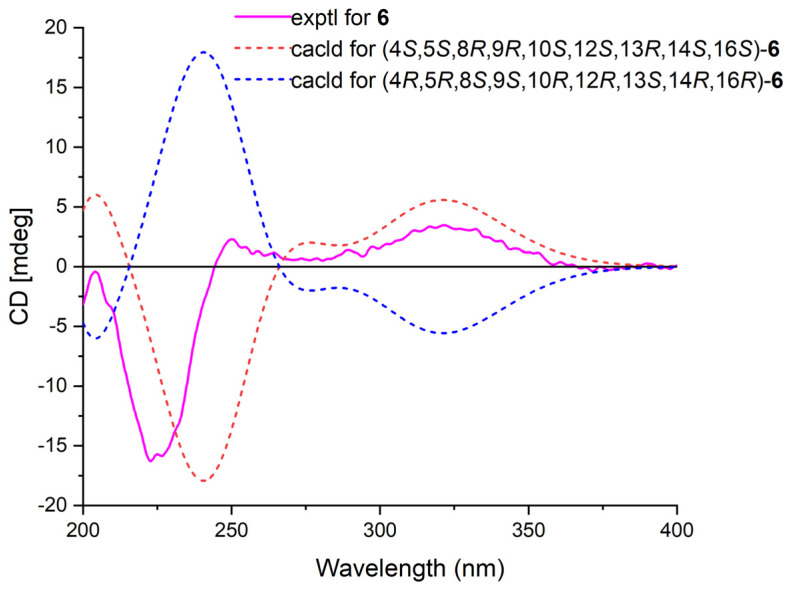
Calculated and experimental ECD spectra of **6**.

Phyllofenone K (**6**) was isolated as a white powder and assigned the molecular formula of C_31_H_50_O_4_ based on HRESIMS data. The NMR data of **6** (Table 2) closely resembled those of **4**, suggesting that they shared the same planar scalarane structure. This hypothesis was confirmed by the HMBC and COSY correlations, as shown in Figure 2. Significant differences between **4** and **6** were observed in the downfield shifts of C-14 (from δ_C_ 43.8 in **4** to δ_C_ 47.3 in **6**) and C-16 (from δ_C_ 63.3 in **4** to δ_C_ 68.0 in **6**). These data indicated that these two compounds were a pair of epimers. Moreover, two groups of NOESY correlations, H-5/H-6α, H-5/H_3_-19, H-6α/H-9, H-7α/H-9, H-7α/H-14, H-7α/H-15α, and H-7α/H-16 and H_2_-20/H_3_-21, H_2_-20/H_3_-22, H-12/H_3_-23, H-15β/H_3_-23, and H_3_-21/H_3_-23, were detected from the NOESY spectrum (Figure 3). These two sets of correlations revealed the cofacial orientation of H-4, H-10, H-14, H-16, and H_3_-19, as well as H-12, H_2_-20, H_3_-21, H_3_-22, and H_3_-23, respectively. Subsequently, the absolute configuration of **6** was determined as 4*S*, 5*S*, 8*R*, 9*R*, 10*S*, 12*S*, 13*R*, 14*S*, 16*S* by comparing its calculated and experimental ECD spectra (Figure 6).

**Figure 7:**
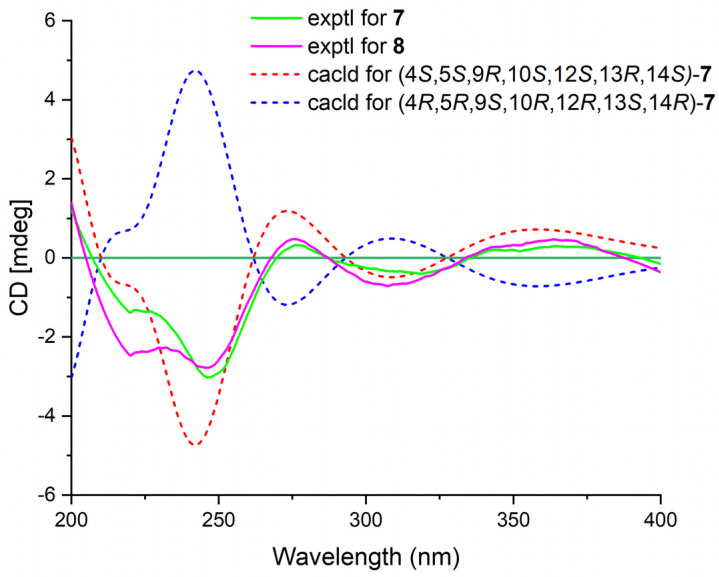
Experimental ECD spectra of **7**–**8** and calculated ECD spectra of **7**.

Phyllofenone L (**7**) was obtained as a white power. Its molecular formula was determined to be C_31_H_48_O_4_ based on the analysis of the HRESIMS spectrum. A detailed comparison of the NMR data (Table 3) between **7** and **4** indicated that they shared an almost identical scalarane skeleton. The main distinction between **4** and **7** was the absence of an oxygenated methine carbon (δ_C_ 63.3) and the presence of a carbonyl carbon (δ_C_ 197.8) at C-16 in **7**. This deduction was supported by the HMBC correlations from H-18 and H_3_-25 to C-17 and C-24, from H_2_-15 to C-13, C-14, C-16, and C-17, and from H_3_-23 to C-13, C-14, and C-18 (Figure 2). The absolute configuration of **7** was determined to be 4*S*, 5*S*, 9*R*, 10*S*, 12*S*, 13*R*, 14*S* through CD spectral comparison (Figure 7), followed by analysis of the NOESY correlations, the coupling constant of H-12, and ^13^C NMR chemical shifts [4,11].

Phyllofenone M (**8**) was also obtained as a white power. The molecular formula, C_32_H_50_O_4_, was deduced from its HRESIMS data (*m*/*z* 521.3608 [M + Na]^+^). Compound **8** showed chemical shifts (Table 3) that were nearly identical to those of **7**, with the only differences being similar to those found between **4** and **5**. Correlations observed in the 2D NMR spectra (Figure 2) confirmed the same scalarane core between **7** and **8**, with the 4-methylpentanoate group substituted at C-12 in **8** instead of the valerate group in **7**. By comparing the CD spectra (Figure 7) and specific rotation values between **7** and **8**, the absolute configuration of **8** was unequivocally established as 4*S*, 5*S*, 9*R*, 10*S*, 12*S*, 13*R*, 14*S*.

The known compounds carteriofenones B and A (**9**–**10**) were also isolated from *P. foliascens* and were completely characterized via a comparison of their NMR data with those previously reported [18].

The cytotoxic activity of compounds **1**–**10** was tested against HeLa, HCT-116, H460, and SW1990 by the CCK-8 method. Among them, only compound **5** exhibited significant cytotoxic activity against the above cancer cell lines, with IC_50_ values ranging from 3.4 to 7.3 μM (Table 4). Comparing the relative potency of these scalaranes revealed that the substitution of a 4-methylpentanoate group at C-12 increased the cytotoxicity compared to a valerate group at C-12. This observation is consistent with previously reported results [4]. Additionally, the α-OH substitution at C-16 in **4**, compared to the β-OH substitution at C-16 in **6**, led to increased cytotoxic activity. Furthermore, these derivatives were also assayed for antibacterial activity against *Vibrio parahaemolyticus*, *V. alginolyticus*, *V. cholerae*, *V. vulnificus*, *Staphylococcus aureus*, and *Escherichia coli* by the minimum inhibitory concentration (MIC) method. Only compounds **4** and **6** displayed weak activity against *S. aureus* and *E. coli*, with MIC values of 16 μg/mL and 8 μg/mL, respectively.

## 3. Materials and Methods

### 3.1. General Experimental Procedures

Optical rotations were recorded on a Perkin-Elmer model 341 polarimeter (Perkin-Elmer Inc., Waltham, MA, USA). The UV and CD spectra were recorded on a UV-8000 spectrophotometer (Shanghai Metash instruments Co., Ltd., Shanghai, China) and a Jasco J-715 spectropolarimeter in MeOH (JASCO, Tokyo, Japan), respectively. The IR spectra were obtained on a VERTEX 70v FT-IR spectrometer (Bruker Biospin Corp., Billerica, MA, USA). The NMR experiments were conducted on a Bruker AMX-500 instrument (Bruker Biospin Corp., Billerica, MA, USA). HRESIMS spectra were recorded on an AB SCIEX Triple Tof-4600 spectrometer (ABSciex, Vaughan ON, Canada). Column chromatographic separations were carried out using silica gel (200–300 mesh, Qingdao Ocean Chemical Co., Ltd., Qingdao, China) and ODS (50 μm, YMC Co., Ltd., Kyoto, Japan). MPLC was carried out on a SepaBean machine (Santai Technology Co., Ltd., Changzhou, China). RP HPLC was performed on a YMC-Pack Pro C18 column (250 × 10 mm, 5 mm, YMC Co., Ltd., Kyoto, Japan) using a Waters 1525 binary HPLC pump with a Waters 2998 photodiode array detector (Waters Corp., Milford, MA, USA). Analytical thin-layer chromatography was performed on silica gel HSGF254 plates and visualized by spraying with anisaldehyde–H_2_SO_4_ reagent.

### 3.2. Sponge Material

The marine sponge was collected off the Woody Island in the South China Sea in December 2021 and authenticated by Prof. Prof. Hou-Wen Lin. A voucher specimen (XS-2021.12) has been deposited at the Naval Medical Center of PLA, Naval Medical University.

### 3.3. Extraction and Isolation

The sponge (0.6 kg, dry weight) was extracted three times with MeOH/CH_2_Cl_2_ (*v*/*v* 1:1, 1 L). The crude extract (29.7 g), obtained after evaporating the MeOH/CH_2_Cl_2_ was separated into 11 fractions (Fr. A−K) by a silica gel column with gradient petroleum ether/EtOAc (100:1, 50:1, 25:1, 10:1, 5:1, 3:1, 1:1, 0:1, *v*/*v*). Fraction F (3.72 g) was subjected to a silica gel column with a gradient elution of petroleum ether/EtOAc (1:0, 20:1, 15:1, 10:1, 5:1, 0:1, *v*/*v*) to afford six subfractions (Fr. F1−F6). Fr. F6 (128.4 mg) was further purified by reversed-phase semi-preparative HPLC (95% CH_3_OH/5% H_2_O, 2.0 mL/min, 236 nm), yielding **1** (5.3 mg, t_R_ = 32.0 min) and **2** (4.5 mg, t_R_ = 36.0 min). Fraction G (4.26 g) was subjected to a silica gel column eluted with a gradient of petroleum ether/EtOAc (100:1, 80:1, 50:1, 30:1, 20:1, 10:1, 5:1, 0:1, *v*/*v*) to obtain eight fractions (Fr. G1−G8). Fr. G3 (64.5 mg) was purified with 90% CH_3_OH/10% H_2_O by HPLC (254 nm, 2.0 mL/min) to afford **7** (5.5 mg, t_R_ = 39.8 min) and **8** (6.3 mg, t_R_ = 45.9 min). Fr. G4 (444.1 mg) was chromatographed over ODS using a gradient elution of MeOH/H_2_O (from 40% to 100%) to give eight fractions, G4a−G4h. Fr. G4b (33.4 mg) was purified by semi-preparative HPLC (90% CH_3_OH/10% H_2_O, 2 mL/min, 225 nm) to provide **5** (2.5 mg, t_R_ = 32.0 min). Fr. G4c (60.1 mg) was separated by HPLC (90% CH_3_OH/10% H_2_O, 2 mL/min, 235 nm) to obtain **9** (5.0 mg, t_R_ = 26.6 min) and **10** (2.6 mg, t_R_ = 28.9 min). Fr. G4d (54.2 mg) was purified with 88% CH_3_CN/12% H_2_O via HPLC (2.0 mL/min, 234 nm) to afford **6** (2.2 mg, t_R_ = 48.0 min) and **3** (2.8 mg, t_R_ = 54.0 min). Fr. G4g (33.0 mg) was further purified with 95% CH_3_CN via HPLC (2.0 mL/min, 238 nm) to afford **4** (2.1 mg, t_R_ = 41.0 min).

Phyllofenone F (**1**): white powder; ${\left[\alpha \right]}_{D}^{25}$ +73.5 (*c* 0.1, MeCN); UV (MeCN) λ_max_ 209 (3.61), 232 (3.93) nm; CD (MeCN) (Δε) 205 (−12.1), 235 (+14.9); IR (KBr) *v*_max_ 2956, 922, 2853, 1723, 1644, 1456, 1383, 1289, 1259, 1240, 1170, 1087, 1033, 956, 817, 801, 732, 604 cm^–1^; HRESIMS *m*/*z* [M + Na]^+^ 493.3653 (Calcd. C_31_H_50_O_3_Na, 493.3652, Δ = 0.2 ppm); for ^1^H and ^13^C NMR data, see Table 1.

Phyllofenone G (**2**): white powder; ${\left[\alpha \right]}_{D}^{25}$ +60.2 (*c* 0.1, MeCN); UV (MeCN) λ_max_ 209 (3.46), 233 (3.85) nm; CD (MeCN) (Δε) 205 (−10.5), 237 (+12.6); IR (KBr) *v*_max_ 2955, 2924, 2870, 2850, 1729, 1666, 1645, 1465, 1348, 1258, 1166, 1122, 1099, 1070, 1033, 1006, 991, 954, 935, 817, 601, 574 cm^–1^; HRESIMS *m*/*z* [M + Na]^+^ 507.3815 (Calcd. C_32_H_52_O_3_Na, 507.3809, Δ = 1.2 ppm); for ^1^H and ^13^C NMR data, see Table 1.

Phyllofenone H (**3**): white powder; ${\left[\alpha \right]}_{D}^{25}$ +78.1 (*c* 0.1, MeCN); UV (MeCN) λ_max_ 229 (3.73) nm; CD (MeCN) (Δε) 208 (−3.5), 235 (+5.3); IR (KBr) *v*_max_ 3459, 2956, 2925, 2871, 2852, 1731, 1668, 1462, 1425, 1384, 1350, 1301, 1257, 1167, 1130, 1099, 1033, 1005, 959, 860, 818, 721, 668, 630, 599, 574 cm^–1^; HRESIMS m/z [M + Na]^+^ 523.3757 (Calcd. C_32_H_52_O_4_Na, 523.3758, Δ = −0.2 ppm); for ^1^H and ^13^C NMR data, see Table 1.

Phyllofenone I (**4**): white powder; ${\left[\alpha \right]}_{D}^{25}$ −53.2 (*c* 0.1, MeCN); UV (MeCN) λ_max_ 205 (4.09), 224 (4.27) nm; CD (MeCN) (Δε) 220 (−23.6), 246 (+6.2), 313 (−4.1); IR (KBr) *v*_max_ 3535, 2957, 2930, 2872, 1729, 1661, 1461, 1381, 1252, 1175, 1093, 1034, 1015, 966, 931, 867, 568 cm^–1^; HRESIMS *m*/*z* [M + Na]^+^ 509.3599 (Calcd. C_31_H_50_O_4_Na, 509.3601, Δ = −0.6 ppm); for ^1^H and ^13^C NMR data, see Table 2.

Phyllofenone J (**5**): white powder; ${\left[\alpha \right]}_{D}^{25}$ −55.6 (*c* 0.1, MeCN); UV (MeCN) λ_max_ 202 (3.73), 226 (4.07) nm; CD (MeCN) (Δε) 223 (−16.9), 245 (+4.5), 313 (−2.9); IR (KBr) *v*_max_ 3540, 2956, 2929, 2871, 1728, 1660, 1462, 1384, 1268, 1225, 1177, 1100, 1034, 1013, 963, 931, 885, 867, 776, 713, 632, 567, 461 cm^–1^; HRESIMS *m*/*z* [M + Na]+ 523.3759 (Calcd. C_32_H_52_O_4_Na, 523.3758 Δ = 0.1 ppm); for ^1^H and ^13^C NMR data, see Table 2.

Phyllofenone K (**6**): white powder; ${\left[\alpha \right]}_{D}^{25}$ −14.9 (*c* 0.1, MeCN); UV (MeCN) λ_max_ 208 (3.94), 228 (4.10) nm; CD (MeCN) (Δε) 223 (−24.0), 250 (+3.4), 322 (+5.1); IR (KBr) *v*_max_ 3235, 2956, 2927, 2871, 1730, 1655, 1462, 1370, 1294, 1252, 1172, 1093, 1077, 1052, 1033, 1004, 885, 675, 632, 604, 517 cm^–1^; HRESIMS *m*/*z* [M + Na]+ 509.3605 (Calcd. C_31_H_50_O_4_Na, 509.3601, Δ = 0.8 ppm); for ^1^H and ^13^C NMR data, see Table 2.

Phyllofenone L (**7**): white powder; ${\left[\alpha \right]}_{D}^{25}$ +14.8 (*c* 0.1, MeCN); UV (MeCN) λ_max_ 211 (3.65), 224 (3.68) nm; CD (MeCN) (Δε) 247 (−4.4), 276 (+0.4), 310 (−1.0), 365 (+0.4); IR (KBr) *v*_max_ 2957, 2929, 2872, 1770, 1733, 1692, 1678, 1601, 1461, 1418, 1381, 1356, 1288, 1272, 1243, 1227, 1168, 1129, 1107, 1087, 1048, 1034, 1004, 984, 962, 925 cm^–1^; HRESIMS *m*/*z* [M + Na]^+^ 507.3438 (Calcd. C_31_H_48_O_4_Na, 507.3445, Δ = −1.5 ppm); for ^1^H and ^13^C NMR data, see Table 3.

Phyllofenone M (**8**): white powder; ${\left[\alpha \right]}_{D}^{25}$ +21.9 (*c* 0.1, MeCN); UV (MeCN) λ_max_ 211 (3.54), 225 (3.58) nm; CD (MeCN) (Δε) 247 (−4.2), 276 (0.7), 309 (−1.0), 365 (+0.7); IR (KBr) *v*_max_ 2956, 2925, 2871, 2852, 1735, 1693, 1679, 1601, 1465, 1417, 1357, 1272, 1243, 1227, 1165, 1110, 1034 cm^–1^; HRESIMS *m*/*z* [M + Na]^+^ 521.3608 (Calcd. C_32_H_50_O_4_Na, 521.3601, Δ = 1.4 ppm); for ^1^H and ^13^C NMR data, see Table 3.

### 3.4. Acid Hydrolysis of Phyllofenone H (***3***)

A solution of phyllofenone H (**3**) (1.0 mg in 6 M HCl, 1 mL) was heated to 110 °C in a sealed vial and maintained at 110 °C for 12 h. Subsequently, the hydrolysate was evaporated to dryness under a stream of dry N_2_ and subjected to purification through a small column (6 × 70 mm) on silica gel (200 mesh) eluted with CH_2_Cl_2_/MeOH (20:1, *v*/*v*) to give (*R*)-3-hydroxy-4-methylpentanoic acid [19].

(*R*)-3-hydroxy-4-methylpentanoic acid: colorless oil; ${\left[\alpha \right]}_{D}^{25}$ +2.1 (*c* 0.10, CHCl_3_); ESIMS *m*/*z* 155.15 [M + Na]^+^; ^1^H NMR (500 MHz, CDCl3): δ_H_ 3.81 (1H, m), 2.49 (1H, dd, 16.5, 3.5), 2.59 (1H, 16.5, 9.0), 1.72 (1H, m), 0.96 (3H, d, 6.5 Hz), 0.95 (3H, d, 6.5 Hz).

### 3.5. Biological Assays

The cytotoxicity of compounds **1**–**10** against the HeLa, HCT-116, H460, and SW1990 human cancer cell lines was determined using the CCK-8 method [4,20], with cisplatin used as a positive control. The antimicrobial activity of compounds **1**–**10** against *Vibrio parahaemolyticus*, *V. alginolyticus*, *V. cholerae*, *V. vulnificus*, *Staphylococcus aureus*, and *Escherichia coli* was evaluated as previously described [4], with levofloxacin used as a positive control.

## 4. Conclusions

Eight new scalarane sesterterpenes, phyllofenones F–M (**1**–**8**), along with two known analogues, carteriofenones B and A (**9**–**10**), were isolated from a South China Sea sponge, *P. foliascens*. In conjunction with our previous chemical study of the same organism, we discovered that a 4-methylpentanoate group substituted at C-12 positively affected the activity. Additionally, compound **5** displayed notable cytotoxicity, highlighting the significance of the α-OH substitution at C-16 for its cytotoxic properties. Collectively, this research contributes to the expansion of the chemical molecular diversity of the scalarane sesterterpene family.

## Figures and Tables

**Table 1:** ^1^H (500 MHz) and ^13^C NMR (125 MHz) spectroscopic data of **1**–**3** in CDCl_3_.

Position	1		2		3	
	δ_C_	δ_H_, Mult. (*J* in Hz)	δ_C_	δ_H_, Mult. (*J* in Hz)	δ_C_	δ_H_, Mult. (*J* in Hz)
1α	40.1, CH_2_	0.63, td (12.0, 6.0)	40.1, CH_2_	0.64, td (12.0, 6.0)	40.0, CH_2_	0.64, td (12.5, 4.0)
1β		1.59, m		1.59, m		1.57, m
2α	18.2, CH_2_	1.43, m	18.2, CH_2_	1.43, m	18.1, CH_2_	1.42, m
2β		1.36, m		1.37, m		1.36, m
3α	36.7, CH_2_	0.83, m	36.7, CH_2_	0.84, m	36.6, CH_2_	0.84, m
3β		1.66, m		1.66, m		1.65, m
4	36.1, C		36.1, C		36.1, C	
5	58.7, CH	0.88, m	58.7, CH	0.86, m	58.6, CH	1.90, m
6	18.0, CH_2_	1.54, m	18.0, CH_2_	1.55, m	17.9, CH_2_	1.52, m
7α	41.7, CH_2_	1.73, m	41.7, CH_2_	1.74, m	41.6, CH_2_	1.73, m
7β		0.99, td (13.0, 4.0)		0.99, m		0.98, m
8	37.4, C		37.4, C		37.4, C	
9	53.0, CH	1.28, dd (12.5, 2.5)	53.0, CH	1.28, dd (12.0, 2.5)	53.0, CH	1.26, dd (12.0, 2.5)
10	36.7, C		36.9, C		36.9, C	
11α	22.3, CH_2_	1.74, m	22.2, CH_2_	1.75, m	22.3, CH_2_	1.70, m
11β		1.62, m		1.63, m		1.77, dt (8.5, 3.5)
12	76.7, CH	4.79, t (2.5)	76.8, CH	4.80, t (2.5)	77.6, CH	4.85, t (2.5)
13	35.9, C		35.9, C		35.8, C	
14	47.9, CH	1.53, m	47.8, CH	1.52, m	47.8, CH	1.52, m
15α	23.9, CH_2_	2.16, m	23.9, CH_2_	2.18, m	23.9, CH_2_	2.18, m
15β		2.28, m		2.29, m		2.27, m
16	139.5, CH	6.85, m	139.5, CH	6.85, m	139.7, CH	6.86, q (2.5)
17	137.7, C		137.7, C		137.5, C	
18α	35.5, CH_2_	2.21, m	35.5, CH_2_	2.21, m	35.3, CH	2.20, m
18β		1.95, d (18.0)		1.95, d (18.5)		1.95, d (16.5)
19	28.5, CH_3_	0.80, s	28.6, CH_3_	0.79, s	28.5, CH_3_	0.79, s
20a	24.5, CH_2_	1.16, m	24.5, CH_2_	1.15, m	24.5, CH_2_	1.15, m
20b		1.54, m		1.54, m		1.51, m
21	15.9, CH_3_	0.90, s	15.9, CH_3_	0.90, s	15.9, CH_3_	0.90, s
22	17.1, CH_3_	0.83, s	17.1, CH_3_	0.83, s	17.1, CH_3_	0.84, s
23	19.9, CH_3_	0.85, s	19.9, CH_3_	0.85, s	19.9, CH_3_	0.86, s
24	199.0, C		199.1, C		199.1, C	
25	25.3, CH_3_	2.28, s	25.3, CH_3_	2.23, s	25.2, CH_3_	2.29, s
26	8.7, CH_3_	0.74, t (7.5)	8.7, CH_3_	0.74, s	8.6, CH_3_	0.74, t (7.5)
1′	172.9, C		173.1, C		172.9, C	
2′a	34.6, CH_2_	2.33, t (7.5)	32.9, CH_2_	2.32, t (7.5)	38.7, CH_2_	2.41, dd (16.5, 9.5)
2′b						2.56, dd (16.5, 2.5)
3′	27.3, CH_2_	1.62, m	34.0, CH_2_	1.54, m	72.7, CH	3.79, t (7.5)
4′	22.4, CH_2_	1.38, m	27.7, CH	1.60, m	33.1, CH	1.71, m
5′	13.8, CH_3_	0.92, t (7.5)	22.3, CH_3_	0.90, d 2.0	17.8, CH_3_	0.97, d (6.5)
6′			22.3, CH_3_	0.90, d 2.0	18.6, CH_3_	0.93, d (6.5)

**Table 2:** ^1^H (500 MHz) and ^13^C NMR (125 MHz) spectroscopic data of **4**–**6** in CDCl_3_.

Position	4		5		6	
	δ_C_	δ_H_, Mult. (*J* in Hz)	δ_C_	δ_H_, Mult. (*J* in Hz)	δ_C_	δ_H_, Mult. (*J* in Hz)
1α	40.1, CH_2_	0.69, td (10.5, 3.0)	40.1, CH_2_	0.67, td (13.0, 4.5)	40.0, CH_2_	0.65, td (11.0, 3.5)
1β		1.56, m		1.55, m		1.56, m
2α	18.3, CH_2_	1.50, m	18.3, CH_2_	1.47, m	17.9, CH_2_	1.47, m
2β		1.36, m		1.34, m		1.34, m
3α	36.6, CH_2_	0.86, m	36.6, CH_2_	0.83, m	36.6, CH_2_	0.84, m
3β		1.67, m		1.65, m		1.66, m
4	36.1, C		36.1, C		36.1, C	
5	58.7, CH	0.95, dd (10.5, 2.0)	58.6, CH	0.93, dd (12.0, 2.0)	58.5, CH	0.90, m
6	17.9, CH_2_	1.59, m	17.9, CH_2_	1.55, m	18.2, CH_2_	1.55, m
7α	41.1, CH_2_	1.84, dt (10.5, 2.5)	41.1, CH_2_	1.81, m	41.2, CH_2_	1.84, dt (10.5, 2.5)
7β		1.14, td (10.5, 2.5)		1.11, td (13.0, 4.0)		0.97, m
8	36.9, C		37.0, C		37.0, C	
9	53.5, CH	1.38, m	53.5, CH	1.38, m	53.4, CH	1.24, m
10	37.0, C		36.9, C		37.2, C	
11α	22.3, CH_2_	1.75, m	22.3, CH_2_	1.74, m	22.1, CH_2_	1.72, m
11β		1.58, m		1.37, m		1.75, m
12	76.1, CH	5.06, t (2.0)	76.2, CH	5.05, t (3.0)	76.1, CH	5.03, t (2.5)
13	41.4, C		41.4, C		41.5, C	
14	43.8, CH	1.88, m	43.7, CH	1.88, m	47.3, CH	1.48, m
15α	25.2, CH_2_	1.58, m	25.2, CH_2_	1.58, m	25.5, CH_2_	1.49, m
15β		1.87, m		1.86, m		2.12, m
16	63.3, CH	4.56, d (4.0)	63.3, CH	4.54, d (4.5)	68.0, CH	4.59, m
17	138.2, C		138.2, C		138.8, C	
18	152.2, CH	6.60, s	152.2, CH	6.59, s	152.4, CH	6.57, s
19	28.5, CH_3_	0.81, s	28.5, CH_3_	0.79, br.s	28.5, CH_3_	0.80, s
20a	24.5, CH_2_	1.19, m	24.4, CH_2_	1.17, m	24.4, CH_2_	1.19, m
20b		1.53, m		1.59, m		1.52, d (6.0)
21	17.1, CH_3_	0.88, s	17.1, CH_3_	0.87, s	17.1, CH_3_	0.89, s
22	16.8, CH_3_	0.86, s	16.8, CH_3_	0.84, s	16.7, CH_3_	0.84, s
23	19.7, CH_3_	1.07, s	19.7, CH_3_	1.05, s	21.1, CH_3_	1.18, s
24	201.5, C		201.5, C		202.1, C	
25	25.4, CH_3_	2.26, s	25.4, CH_3_	2.24, s	25.6, CH_3_	2.23, s
26	8.6, CH_3_	0.76, t (6.5)	8.7, CH_3_	0.74, t (4.5)	8.6, CH_3_	0.74, t (6.0)
1′	173.6, C		173.8, C		173.4, C	
2′	34.6, CH_2_	2.31, td (6.0, 1.0)	32.9, CH_2_	2.30, t (7.5)	34.5, CH_2_	2.28, dd (6.0, 1.5)
3′	27.5, CH_2_	1.58, m	34.2, CH_2_	1.48, m	27.4, CH_2_	1.57, m
4′	22.3, CH_2_	1.35, m	27.6, CH	1.55, m	22.3, CH_2_	1.34, dd (12.5, 6.0)
5′	13.8, CH_3_	0.90, t (6.0)	22.3, CH_3_	0.87, d (3.5)	13.8, CH_3_	0.90, t (6.0)
6′			22.2, CH_3_	0.88, d (3.5)		

**Table 3:** ^1^H (500 MHz) and ^13^C NMR (125 MHz) spectroscopic data of **1** in CDCl_3_.

Position	7		8	
	δ_C_	δ_H_, Mult. (*J* in Hz)	δ_C_	δ_H_, Mult. (*J* in Hz)
1α	40.0, CH_2_	0.65, td (11.0, 3.5)	40.0, CH_2_	0.63, td (13.5, 4.0)
1β		1.58, m		1.56, m
2α	17.8, CH_2_	1.47, m	18.2, CH_2_	1.47, m
2β		1.36, m		1.35, m
3α	36.6, CH_2_	0.85, m	36.6, CH_2_	0.82, m
3β		1.68, m		1.67, m
4	36.1, C		36.1, C	
5	58.8, CH	0.90, m	58.8, CH	0.88, m
6α	17.8, CH_2_	1.59, m	17.8, CH_2_	1.57, m
6β				
7α	40.8, CH_2_	1.75, m	40.8, CH_2_	1.70, m
7β		1.04, td (10.5, 3.0)		1.01, td (12.0, 3.5)
8	37.3, C		37.3, C	
9	53.0, CH	1.27, m	53.0, CH	1.25, m
10	37.0, C		37.0, C	
11α	22.0, CH_2_	1.87, dt (11.0, 1.5)	22.0, CH_2_	1.85, dt (15.0, 3.0)
11β		1.73, m		1.73, m
12	75.8, CH	5.08, t (2.5)	75.8, CH	5.04, t (3.0)
13	41.3, C		41.3, C	
14	48.7, CH	2.13, dd (12.0, 9.0)	48.7, CH	2.09, dd (14.5, 4.0)
15α	34.9, CH_2_	2.43, m	34.9, CH_2_	2.40, m
15β		2.52, dd (14.5, 3.5)		2.50, dd (17.5, 4.0)
16	197.8, C		197.1, C	
17	136.5, C		136.5, C	
18	163.9, CH	7.31, s	163.9, CH	7.29, s
19	28.5, CH_3_	0.82, s	28.5, CH_3_	0.82, s
20a	24.5, CH_2_	1.18, m	24.5, CH_2_	1.15, m
20b		1.54, m		1.51, m
21	16.4, CH_3_	0.96, s	16.4, CH_3_	0.93, s
22	16.9, CH_3_	0.87, s	16.9, CH_3_	0.84, s
23	18.5, CH_3_	1.17, s	18.5, CH_3_	1.15, s
24	197.9, C		197.8, C	
25	30.6, CH_3_	2.44, s	30.7, CH_3_	2.42, s
26	8.7, CH_3_	0.76, t (6.0)	8.6, CH_3_	0.80, t (7.5)
1′	173.0, C		173.2, C	
2′	34.4, CH_2_	2.31, t (6.5)	32.7 CH_2_	2.29, t (8.0)
3′	27.3, CH_2_	1.58, m	34.1, CH_2_	1.48, m
4′	22.3, CH_2_	1.36, m	27.6, CH	1.56, m
5′	13.8, CH_3_	0.92, t (4.0)	22.2, CH_3_	0.88, d (5.0)
6′			22.3, CH_3_	0.89, d (5.0)

**Table 4:** Cytotoxicity of compounds **1**−**10** to human cancer cell lines (IC_50_ in μM).

Compd	HeLa	HCT-116	H460	SW1990
**1**	>20	>20	>20	>20
**2**	11.8	19.8	18.5	9.8
**3**	NT	NT	NT	NT
**4**	15.3	17.2	15.3	13.2
**5**	3.4	7.3	5.9	3.5
**6**	>20	>20	>20	>20
**7**	18.8	16.2	12.3	11.5
**8**	6.9	7.2	10.1	9.2
**9**	>20	>20	>20	>20
**10**	14.4	16.2	17.7	14.6
Cisplatin	0.5	2.6	2.8	1.1

## Data Availability

The data presented in this study are available on request from the corresponding author.

## Supplementary Materials

The following are available online at https://www.mdpi.com/article/10.3390/md21100507/s1, S1. Quantum chemical CD calculation of compound **1**; S2. Quantum chemical CD calculation of compound **4**; S3. Quantum chemical CD calculation of compound **6**; S4. Quantum chemical CD calculation of compound **7**; S5. ^1^H NMR spectrum of phyllofenone F (**1**) in CDCl_3_; S6. ^13^C NMR spectrum of phyllofenone F (**1**) in CDCl_3_; S7. DEPT135 spectrum of phyllofenone F (**1**) in CDCl_3_; S8. HSQC spectrum of phyllofenone F (**1**) in CDCl_3_; S9. COSY spectrum of phyllofenone F (**1**) in CDCl_3_; S10. HMBC spectrum of phyllofenone F (**1**) in CDCl_3_; S11. NOESY spectrum of phyllofenone F (**1**) in CDCl_3_; S12. HRESIMS of phyllofenone F (**1**); S13. UV spectrum of phyllofenone F (**1**); S14. IR spectrum of phyllofenone F (**1**) (KBr); S15. ^1^H NMR spectrum of phyllofenone G (**2**) in CDCl_3_; S16. ^13^C NMR spectrum of phyllofenone G (**2**) in CDCl_3_; S17. DEPT135 spectrum of phyllofenone G (**2**) in CDCl_3_; S18. HSQC spectrum of phyllofenone G (**2**) in CDCl_3_; S19. COSY spectrum of phyllofenone G (**2**) in CDCl_3_; S20. HMBC spectrum of phyllofenone G (**2**) in CDCl_3_; S21. NOESY spectrum of phyllofenone G (**2**) in CDCl_3_; S22. HRESIMS of phyllofenone G (**2**); S23. UV spectrum of phyllofenone G (**2**) in MeOH; S24 IR spectrum of phyllofenone G (**2**) (KBr); S25. ^1^H NMR spectrum of phyllofenone H (**3**) in CDCl_3_; S26. ^13^C NMR spectrum of phyllofenone H (**3**) in CDCl_3_; S27. DEPT135 spectrum of phyllofenone H (**3**) in CDCl_3_; S28. HSQC spectrum of phyllofenone H (**3**) in CDCl_3_; S29. COSY spectrum of phyllofenone H (**3**) in CDCl_3_; S30. HMBC spectrum of phyllofenone H (**3**) in CDCl_3_; S31. NOESY spectrum of phyllofenone H (**3**) in CDCl_3_; S32. HRESIMS of phyllofenone H (**3**); S33. UV spectrum of phyllofenone H (**3**). S34. IR spectrum of phyllofenone H (**3**) (KBr); S35. ^1^H NMR spectrum of phyllofenone I (**4**) in CDCl_3_; S36. ^13^C NMR spectrum of phyllofenone I (**4**) in CDCl_3_; S37. DEPT135 spectrum of phyllofenone I (**4**) in CDCl_3_; S38. HSQC spectrum of phyllofenone I (**4**) in CDCl_3_; S39. COSY spectrum of phyllofenone I (**4**) in CDCl_3_; S40. HMBC spectrum of phyllofenone I (**4**) in CDCl_3_; S41. NOESY spectrum of phyllofenone I (**4**) in CDCl_3_; S42. HRESIMS of phyllofenone I (**4**); S43. UV spectrum of phyllofenone I (**4**); S44. IR spectrum of phyllofenone I (**4**) (KBr); S45. ^1^H NMR spectrum of phyllofenone J (**5**) in CDCl_3_; S46. ^13^C NMR spectrum of phyllofenone J (**5**) in CDCl_3_; S47. DEPT135 spectrum of phyllofenone J (**5**) in CDCl_3_; S48. HSQC spectrum of phyllofenone J (**5**) in CDCl_3_; S49. COSY spectrum of phyllofenone J (**5**) in CDCl_3_; S50. HMBC spectrum of phyllofenone J (**5**) in CDCl_3_; S51. NOESY spectrum of phyllofenone J (**5**) in CDCl_3_; S52. HRESIMS of phyllofenone J (**5**); S53. UV spectrum of phyllofenone J (**5**); S54. IR spectrum of phyllofenone J (**5**) (KBr); S55. ^1^H NMR spectrum of phyllofenone K (**6**) in CDCl_3_; S56. ^13^C NMR spectrum of phyllofenone K (**6**) in CDCl_3_; S57. DEPT135 spectrum of phyllofenone K (**6**) in CDCl_3_; S58. HSQC spectrum of phyllofenone K (**6**) in CDCl_3_; S59. COSY spectrum of phyllofenone K (**6**) in CDCl_3_; S60. HMBC spectrum of phyllofenone K (**6**) in CDCl_3_; S61. NOESY spectrum of phyllofenone K (**6**) in CDCl_3_; S62. HRESIMS of phyllofenone K (**6**); S63. UV spectrum of phyllofenone K (**6**); S64. IR spectrum of phyllofenone K (**6**) (KBr); S65. ^1^H NMR spectrum of phyllofenone L (**7**) in CDCl_3_; S66. ^13^C NMR spectrum of phyllofenone L (**7**) in CDCl_3_; S67. DEPT135 spectrum of phyllofenone L (**7**) in CDCl_3_; S68. HSQC spectrum of phyllofenone L (**7**) in CDCl_3_; S69. COSY spectrum of phyllofenone L (**7**) in CDCl_3_; S70. HMBC spectrum of phyllofenone L (**7**) in CDCl_3_; S71. NOESY spectrum of phyllofenone L (**7**) in CDCl_3_; S72. HRESIMS of phyllofenone L (**7**); S73. UV spectrum of phyllofenone L (**7**) in MeOH; S74. IR spectrum of phyllofenone L (**7**) (KBr); S75. ^1^H NMR spectrum of phyllofenone M (**8**) in CDCl_3_; S76. ^13^C NMR spectrum of phyllofenone M (**8**) in CDCl_3_; S77. DEPT135 spectrum of phyllofenone M (**8**) in CDCl_3_; S78. HSQC spectrum of phyllofenone M (**8**) in CDCl_3_; S79. COSY spectrum of phyllofenone M (**8**) in CDCl_3_; S80. HMBC spectrum of phyllofenone M (**8**) in CDCl_3_; S81. NOESY spectrum of phyllofenone M (**8**) in CDCl_3_; S82. HRESIMS of phyllofenone M (**8**); S83. UV spectrum of phyllofenone M (**8**); S84. IR spectrum of phyllofenone M (**8**) (KBr); S85. The structure of phyllofenone A.
