# Supplementary material for: Phyllofenones F–M, Scalarane Sesterterpenes from the Marine Sponge Phyllospongia foliascens
Source: Mar Drugs. 2023 Sep 26;21(10):507. doi: 10.3390/md21100507 (PMC10608082; doi:10.3390/md21100507)

Supplementary data

Phyllofenones F–M, Scalarane Sesterterpenes from the Marine Sponge *Phyllospongia foliascens*

Hao-Bing Yu [1,†], Bo Hu [1,†], Zhe Ning [1], Ying He [1], Xiao-Ling Men [1,2], Zi-Fei Yin [1,3], Bing-Hua Jiao [1,4], Xiao-Yu Liu [1,*] and Hou-Wen Lin [2,*]

1. Department of Marine Biomedicine and Polar Medicine, Naval Medical Center of PLA, Naval Medical University, Shanghai 200433, China
2. Research Center for Marine Drugs, State Key Laboratory of Oncogenes and Related Genes, School of Medicine, Shanghai Jiao Tong University, Shanghai 200127, China
3. School of Traditional Chinese Medicine, Naval Medical University, Shanghai 200433, China
4. Department of Biochemistry and Molecular Biology, College of Basic Medical Sciences, Naval Medical University, Shanghai 200433, China

* Correspondence: biolxy@163.com (X.-Y. L.); hwlin@sjtu.edu.cn (H.-W. L.); Tel.: +86-21-81883267

† These authors contributed equally to this work.

S1. Quantum chemical CD calculation of compound **1**.
S2. Quantum chemical CD calculation of compound **4**.
S3. Quantum chemical CD calculation of compound **6**.
S4. Quantum chemical CD calculation of compound **7**.
S5. ^{1}H NMR spectrum of phyllofenone F (**1**) in $CDCl_3$.
S6. ^{13}C NMR spectrum of phyllofenone F (**1**) in $CDCl_3$.
S7. DEPT135 spectrum of phyllofenone F (**1**) in $CDCl_3$.
S8. HSQC spectrum of phyllofenone F (**1**) in $CDCl_3$.
S9. COSY spectrum of phyllofenone F (**1**) in $CDCl_3$.
S10. HMBC spectrum of phyllofenone F (**1**) in $CDCl_3$.
S11. NOESY spectrum of phyllofenone F (**1**) in $CDCl_3$.
S12. HRESIMS of phyllofenone F (**1**).
S13. UV spectrum of phyllofenone F (**1**).
S14. IR spectrum of phyllofenone F (**1**) (KBr).
S15. ^{1}H NMR spectrum of phyllofenone G (**2**) in $CDCl_3$.
S16. ^{13}C NMR spectrum of phyllofenone G (**2**) in $CDCl_3$.
S17. DEPT135 spectrum of phyllofenone G (**2**) in $CDCl_3$.
S18. HSQC spectrum of phyllofenone G (**2**) in $CDCl_3$.
S19. COSY spectrum of phyllofenone G (**2**) in $CDCl_3$.
S20. HMBC spectrum of phyllofenone G (**2**) in $CDCl_3$.
S21. NOESY spectrum of phyllofenone G (**2**) in $CDCl_3$.
S22. HRESIMS of phyllofenone G (**2**).
S23. UV spectrum of phyllofenone G (**2**) in MeOH.
S24 IR spectrum of phyllofenone G (**2**) (KBr).
S25. ^{1}H NMR spectrum of phyllofenone H (**3**) in $CDCl_3$.
S26. ^{13}C NMR spectrum of phyllofenone H (**3**) in $CDCl_3$.
S27. DEPT135 spectrum of phyllofenone H (**3**) in $CDCl_3$.
S28. HSQC spectrum of phyllofenone H (**3**) in $CDCl_3$.
S29. COSY spectrum of phyllofenone H (**3**) in $CDCl_3$.
S30. HMBC spectrum of phyllofenone H (**3**) in $CDCl_3$.
S31. NOESY spectrum of phyllofenone H (**3**) in $CDCl_3$.
S32. HRESIMS of phyllofenone H (**3**).
S33. UV spectrum of phyllofenone H (**3**).
S34. IR spectrum of phyllofenone H (**3**) (KBr).
S35. ^{1}H NMR spectrum of phyllofenone I (**4**) in $CDCl_3$.
S36. ^{13}C NMR spectrum of phyllofenone I (**4**) in $CDCl_3$.
S37. DEPT135 spectrum of phyllofenone I (**4**) in $CDCl_3$.
S38. HSQC spectrum of phyllofenone I (**4**) in $CDCl_3$.
S39. COSY spectrum of phyllofenone I (**4**) in $CDCl_3$.
S40. HMBC spectrum of phyllofenone I (**4**) in $CDCl_3$.
S41. NOESY spectrum of phyllofenone I (**4**) in $CDCl_3$.
S42. HRESIMS of phyllofenone I (**4**).
S43. UV spectrum of phyllofenone I (**4**).
S44. IR spectrum of phyllofenone I (**4**) (KBr).

S45. ^{1}H NMR spectrum of phyllofenone J (**5**) in $CDCl_3$.
S46. ^{13}C NMR spectrum of phyllofenone J (**5**) in $CDCl_3$.
S47. DEPT135 spectrum of phyllofenone J (**5**) in $CDCl_3$.
S48. HSQC spectrum of phyllofenone J (**5**) in $CDCl_3$.
S49. COSY spectrum of phyllofenone J (**5**) in $CDCl_3$.
S50. HMBC spectrum of phyllofenone J (**5**) in $CDCl_3$.
S51. NOESY spectrum of phyllofenone J (**5**) in $CDCl_3$.
S52. HRESIMS of phyllofenone J (**5**).
S53. UV spectrum of phyllofenone J (**5**).
S54. IR spectrum of phyllofenone J (**5**) (KBr).
S55. ^{1}H NMR spectrum of phyllofenone K (**6**) in $CDCl_3$.
S56. ^{13}C NMR spectrum of phyllofenone K (**6**) in $CDCl_3$.
S57. DEPT135 spectrum of phyllofenone K (**6**) in $CDCl_3$.
S58. HSQC spectrum of phyllofenone K (**6**) in $CDCl_3$.
S59. COSY spectrum of phyllofenone K (**6**) in $CDCl_3$.
S60. HMBC spectrum of phyllofenone K (**6**) in $CDCl_3$.
S61. NOESY spectrum of phyllofenone K (**6**) in $CDCl_3$.
S62. HRESIMS of phyllofenone K (**6**).
S63. UV spectrum of phyllofenone K (**6**).
S64. IR spectrum of phyllofenone K (**6**) (KBr).
S65. ^{1}H NMR spectrum of phyllofenone L (**7**) in $CDCl_3$.
S66. ^{13}C NMR spectrum of phyllofenone L (**7**) in $CDCl_3$.
S67. DEPT135 spectrum of phyllofenone L (**7**) in $CDCl_3$.
S68. HSQC spectrum of phyllofenone L (**7**) in $CDCl_3$.
S69. COSY spectrum of phyllofenone L (**7**) in $CDCl_3$.
S70. HMBC spectrum of phyllofenone L (**7**) in $CDCl_3$.
S71. NOESY spectrum of phyllofenone L (**7**) in $CDCl_3$.
S72. HRESIMS of phyllofenone L (**7**).
S73. UV spectrum of phyllofenone L (**7**) in MeOH.
S74. IR spectrum of phyllofenone L (**7**) (KBr).
S75. ^{1}H NMR spectrum of phyllofenone M (**8**) in $CDCl_3$.
S76. ^{13}C NMR spectrum of phyllofenone M (**8**) in $CDCl_3$.
S77. DEPT135 spectrum of phyllofenone M (**8**) in $CDCl_3$.
S78. HSQC spectrum of phyllofenone M (**8**) in $CDCl_3$.
S79. COSY spectrum of phyllofenone M (**8**) in $CDCl_3$.
S80. HMBC spectrum of phyllofenone M (**8**) in $CDCl_3$.
S81. NOESY spectrum of phyllofenone M (**8**) in $CDCl_3$.
S82. HRESIMS of phyllofenone M (**8**).
S83. UV spectrum of phyllofenone M (**8**).
S84. IR spectrum of phyllofenone M (**8**) (KBr).
S85. The structure of phyllofenone A.

S1. Quantum chemical CD calculation of compound **1**.

Conformational analysis was initially performed using Spartan'14 software (Wavefunction, Inc., Irvine, CA, USA) at MMFF94 force field. The conformers with Boltzmann-population of over 5% were chosen for ECD calculations, and then the conformers were initially optimized at B3LYP/DGDZVP level in MeOH using the continuum polarizable continuum model (CPCM). Harmonic vibration frequencies were calculated to confirm the stability of these conformers. As revealed by the frequency analysis, no imaginary frequencies were observed in ground states. The theoretical calculation of ECD was conducted in MeOH using Time-dependent Density functional theory (TD-DFT) at the B3LYP//DGDZVP level for all conformers of compound **1**. The CD spectra were generated by the program GaussView 6.0 (University of Würzburg, Würzburg, Germany) using a Gaussian band shape with 0.3 eV exponential half-width from dipole-length dipolar and rotational strengths.

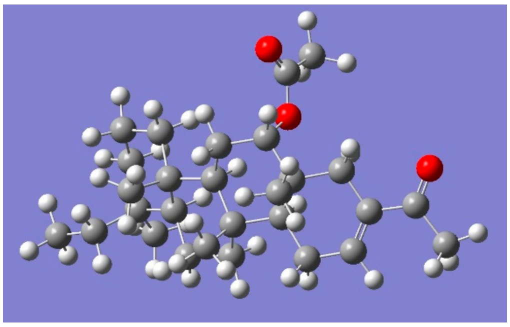

1a

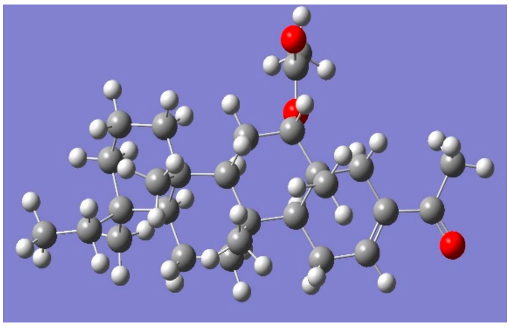

1b

	Conformer	ΔE(kcal/mol)	Population(%)
compound Model-**1**	**1a**	0	76.9
	**1b**	0.71	23.1

S2. Quantum chemical CD calculation of compound **4**.

Conformational analysis was initially performed using Spartan'14 software (Wavefunction, Inc., Irvine, CA, USA) at MMFF94 force field. The conformers with Boltzmann-population of over 5% were chosen for ECD calculations, and then the conformers were initially optimized at B3LYP/6-311G level in MeOH using the continuum polarizable continuum model (CPCM). Harmonic vibration frequencies were calculated to confirm the stability of these conformers. As revealed by the frequency analysis, no imaginary frequencies were observed in ground states. The theoretical calculation of ECD was conducted in MeOH using Time-dependent Density functional theory (TD-DFT) at the B3LYP/6-311G+ level for all conformers of compound **4**. The CD spectra were generated by the program GaussView 6.0 (University of Würzburg, Würzburg, Germany) using a Gaussian band shape with 0.3 eV exponential half-width from dipole-length dipolar and rotational strengths.

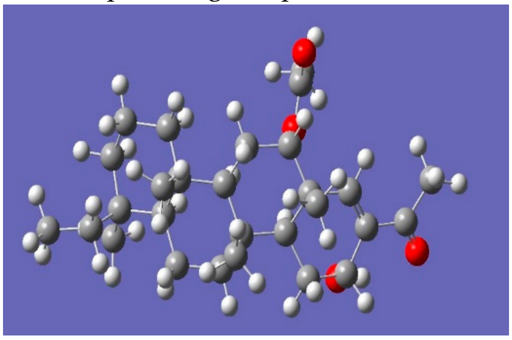

4a

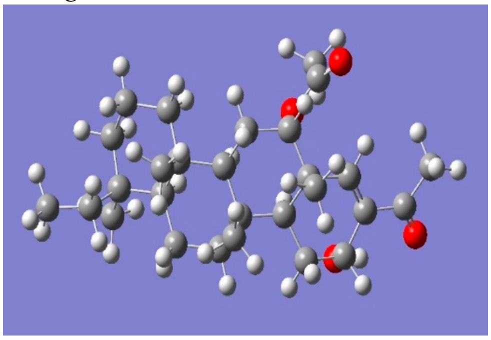

4b

	Conformer	ΔE(kcal/mol)	Population(%)
compound Model-**4**	**4a**	0	73.1
	**4b**	0.59	26.9

S3. Quantum chemical CD calculation of compound **6**.

Conformational analysis was initially performed using Spartan'14 software (Wavefunction, Inc., Irvine, CA, USA) at MMFF94 force field. The conformers with Boltzmann-population of over 5% were chosen for ECD calculations, and then the conformers were initially optimized at B3LYP/6-311G level in MeOH using the continuum polarizable continuum model (CPCM). Harmonic vibration frequencies were calculated to confirm the stability of these conformers. As revealed by the frequency analysis, no imaginary frequencies were observed in ground states. The theoretical calculation of ECD was conducted in MeOH using Time-dependent Density functional theory (TD-DFT) at the B3LYP/6-311G+ level for all conformers of compound **6**. The CD spectra were generated by the program GaussView 6.0 (University of Würzburg, Würzburg, Germany) using a Gaussian band shape with 0.3 eV exponential half-width from dipole-length dipolar and rotational strengths.

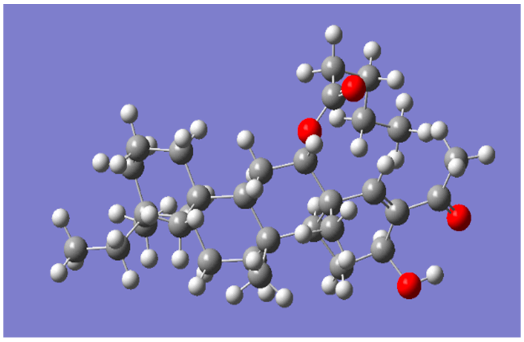

6a

compound Model-**6**	Conformer	ΔE(kcal/mol)	Population(%)
	6a	0	99.9%

S4. Quantum chemical CD calculation of compound **7**.

Conformational analysis was initially performed using Spartan’14 software (Wavefunction, Inc., Irvine, CA, USA) at MMFF94 force field. The conformers with Boltzmann-population of over 5% were chosen for ECD calculations, and then the conformers were initially optimized at B3LYP/6-311G level in MeOH using the continuum polarizable continuum model (CPCM). Harmonic vibration frequencies were calculated to confirm the stability of these conformers. As revealed by the frequency analysis, no imaginary frequencies were observed in ground states. The theoretical calculation of ECD was conducted in MeOH using Time-dependent Density functional theory (TD-DFT) at the B3LYP/6-311G+ level for all conformers of compound **7**. The CD spectra were generated by the program GaussView 6.0 (University of Würzburg, Würzburg, Germany) using a Gaussian band shape with 0.3 eV exponential half-width from dipole-length dipolar and rotational strengths.

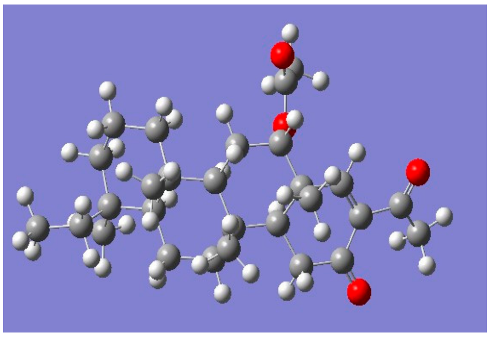

7a

compound Model-7	Conformer	ΔE(kcal/mol)	Population(%)
	**7a**	0	99.9%

S5. ^{1}H NMR spectrum of phyllofenone F (**1**) in $CDCl_3$.

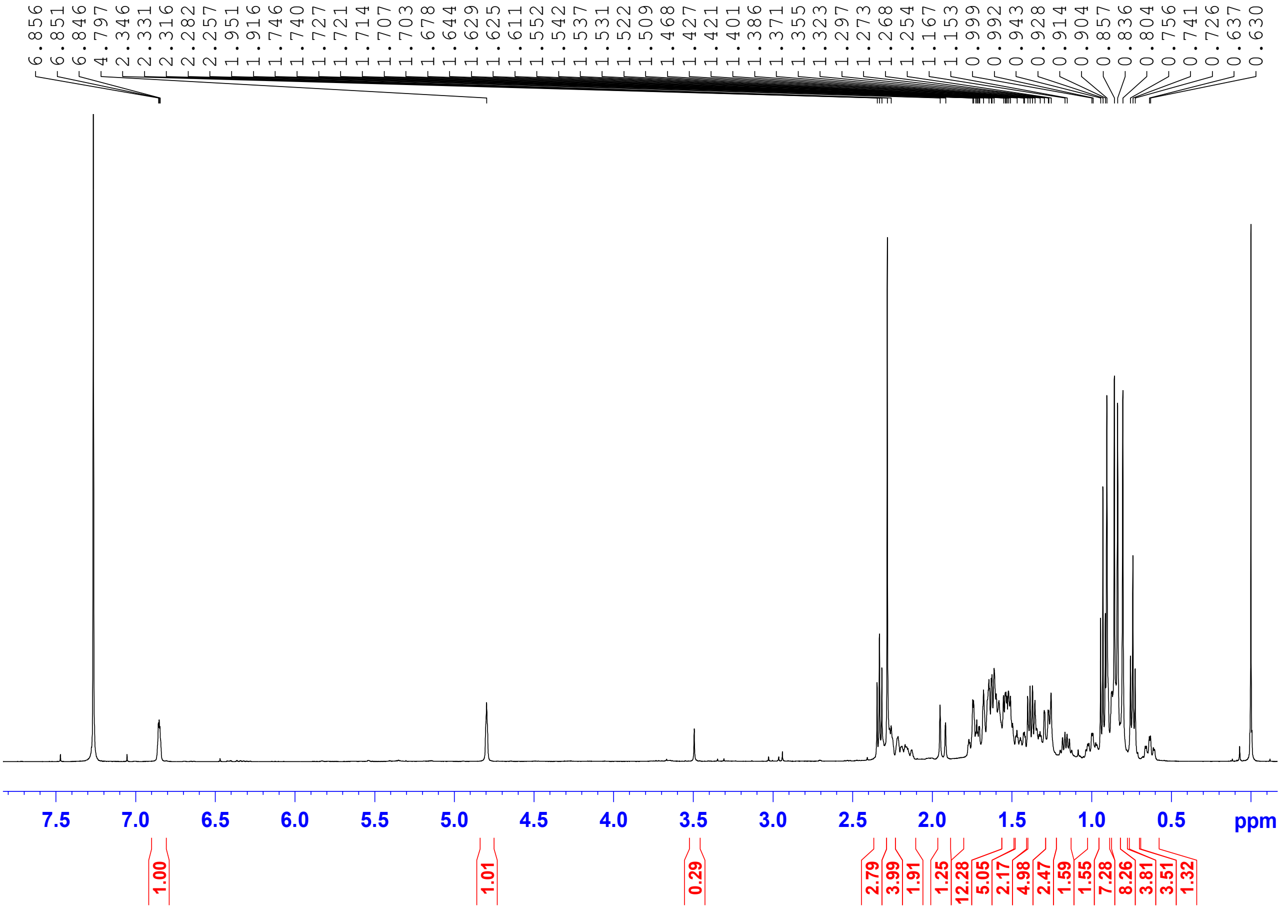

S6. ^{13}C NMR spectrum of phyllofenone F (**1**) in $CDCl_3$.

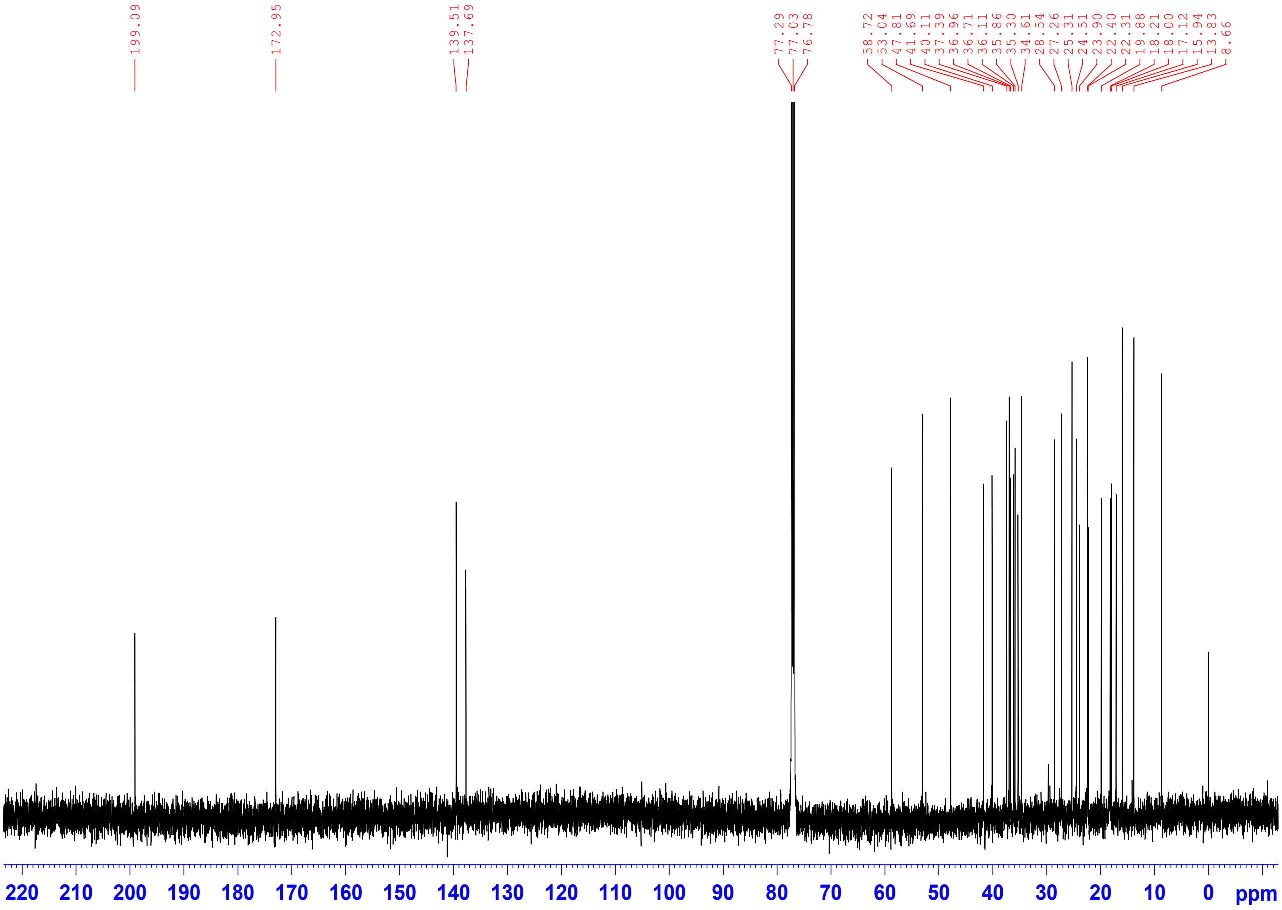

S7. DEPT135 spectrum of phyllofenone F (**1**) in $CDCl_3$.

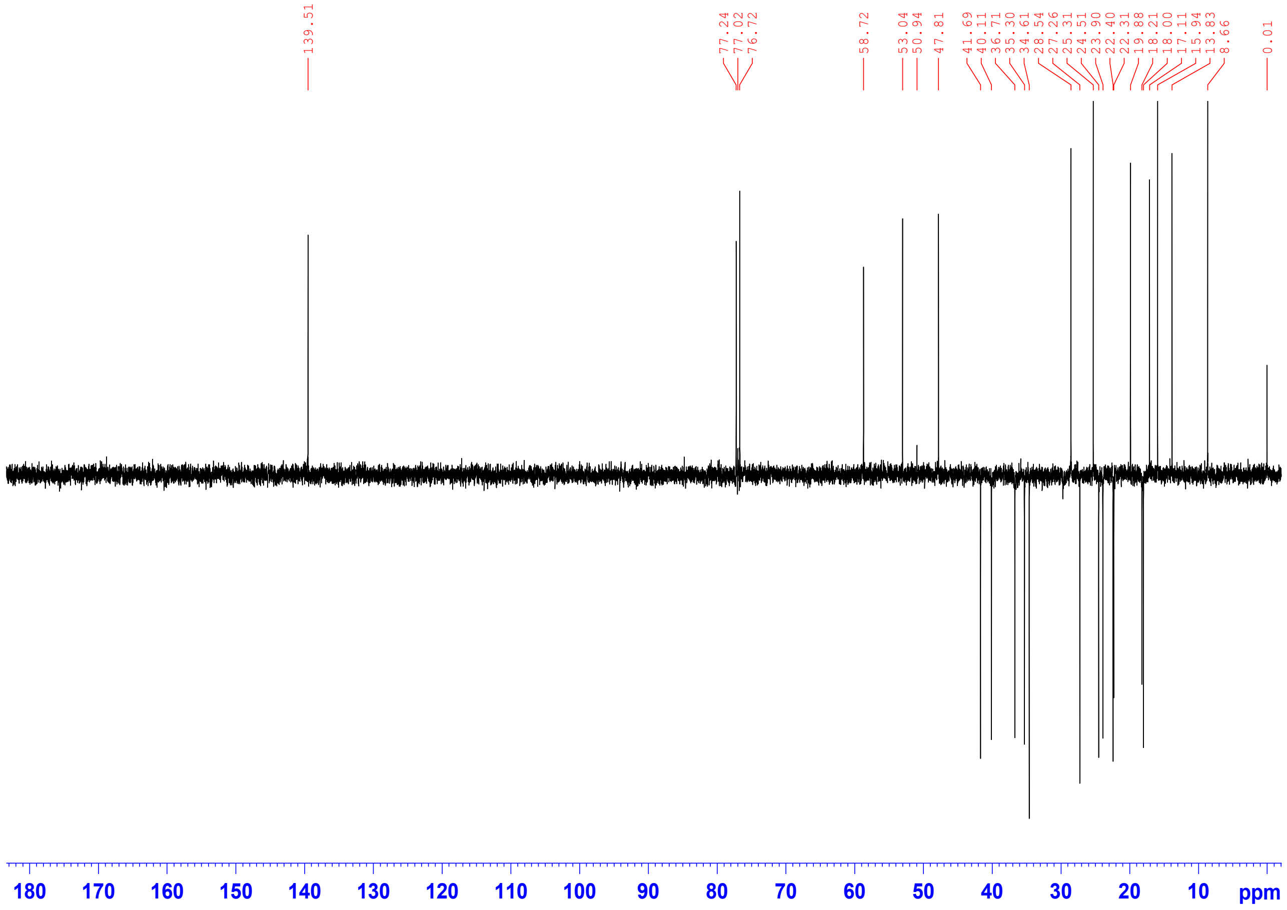

S8. HSQC spectrum of phyllofenone F (**1**) in $CDCl_3$.

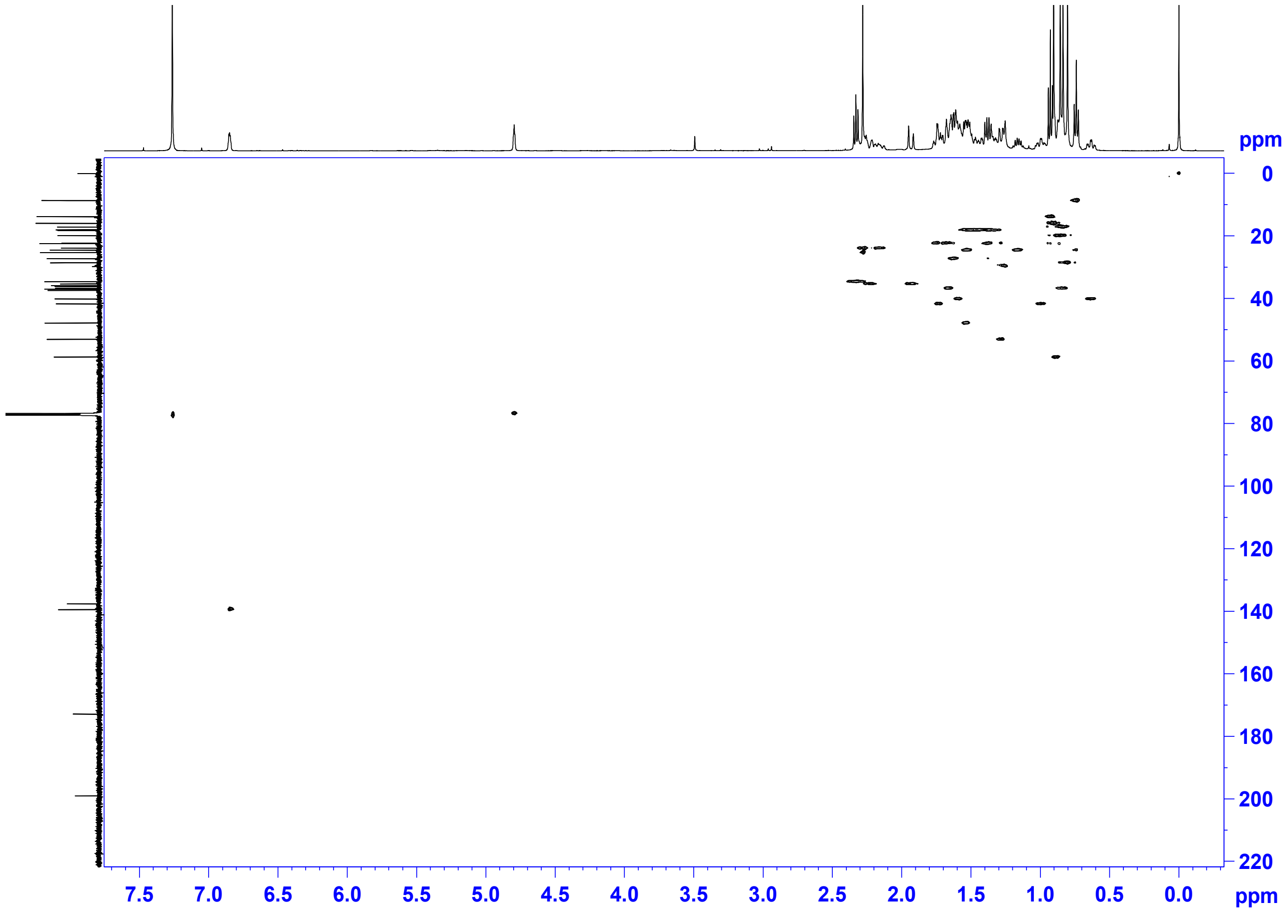

S9. COSY spectrum of phyllofenone F (**1**) in $CDCl_3$.

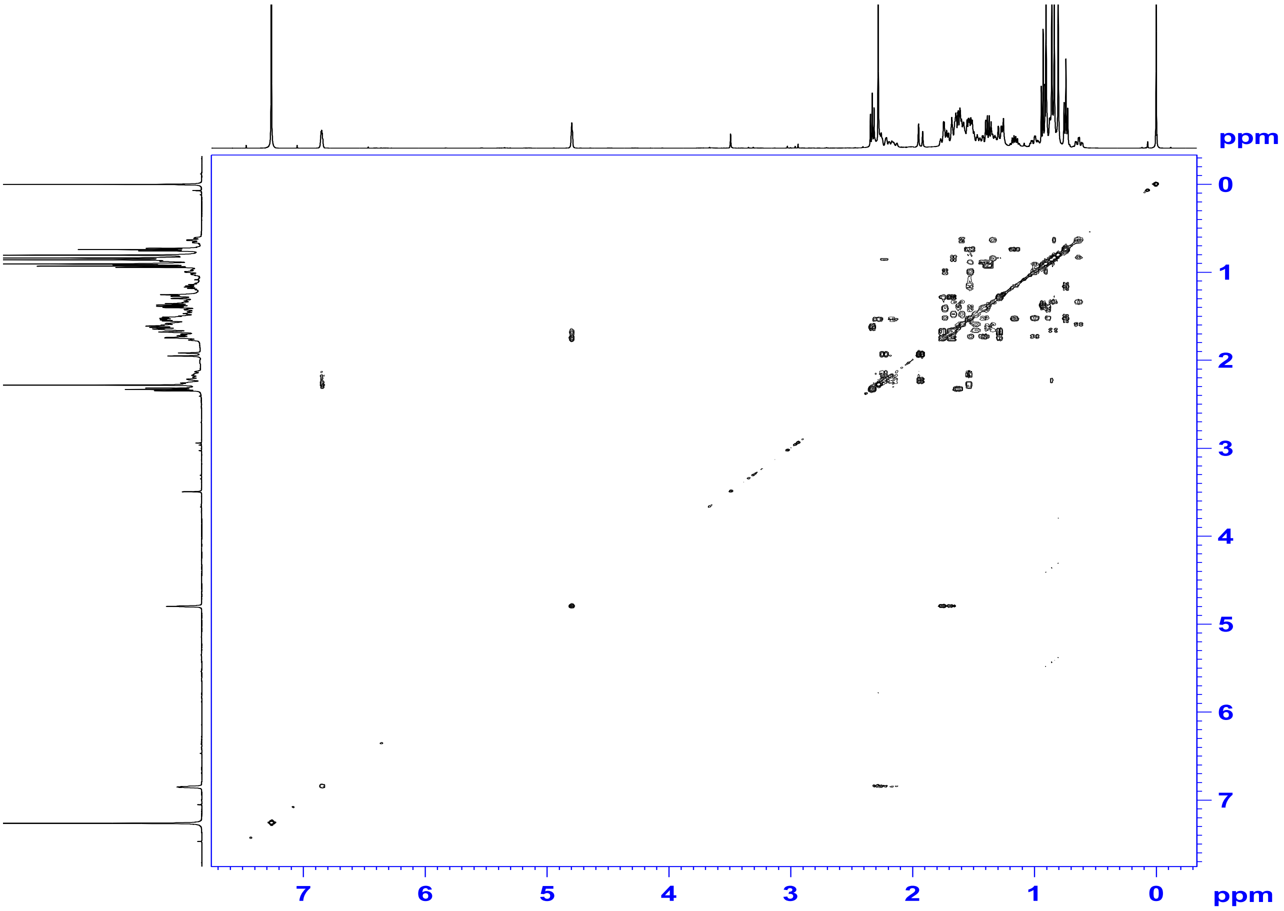

S10. HMBC spectrum of phyllofenone F (**1**) in $CDCl_3$.

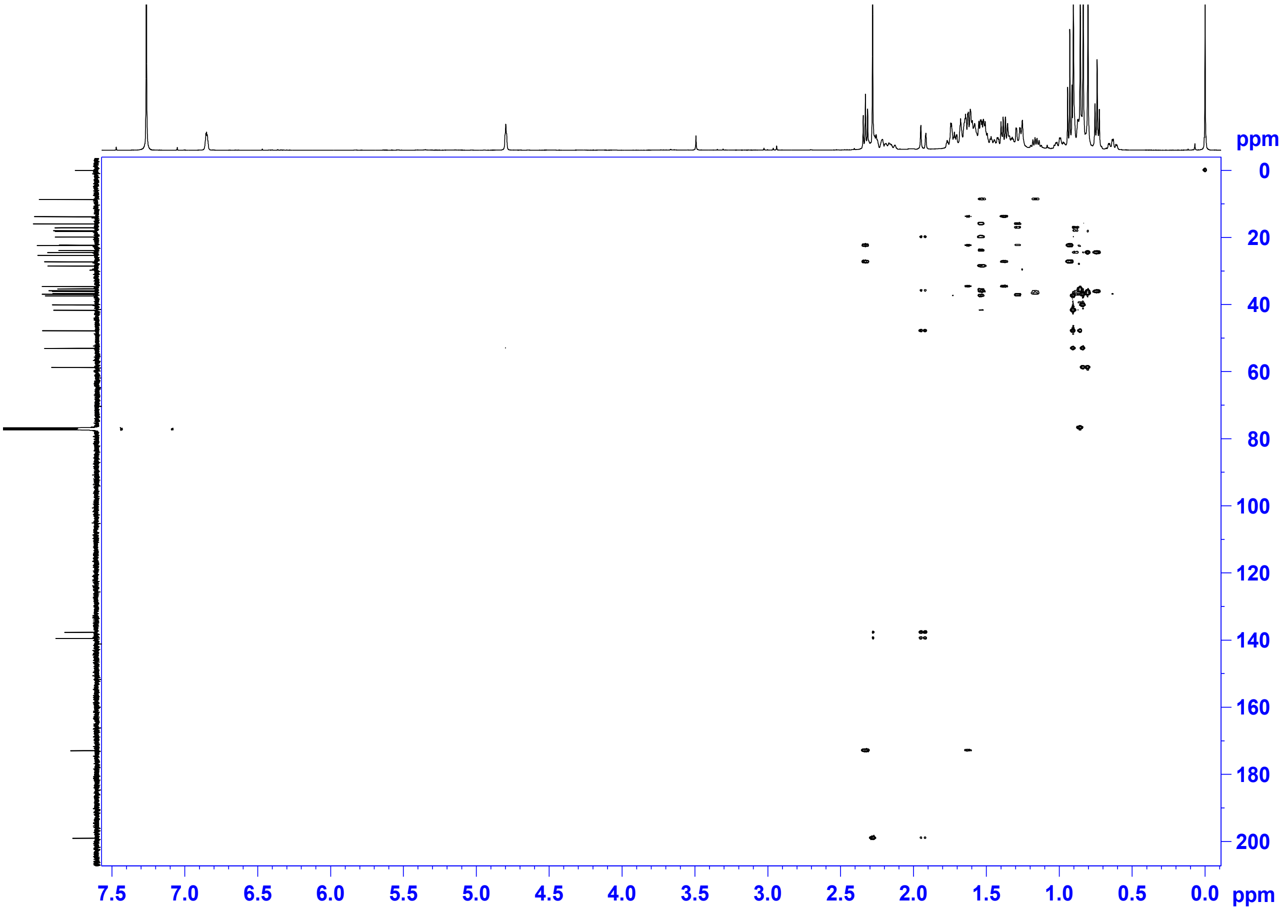

S11. NOESY spectrum of phyllofenone F (**1**) in $CDCl_3$.

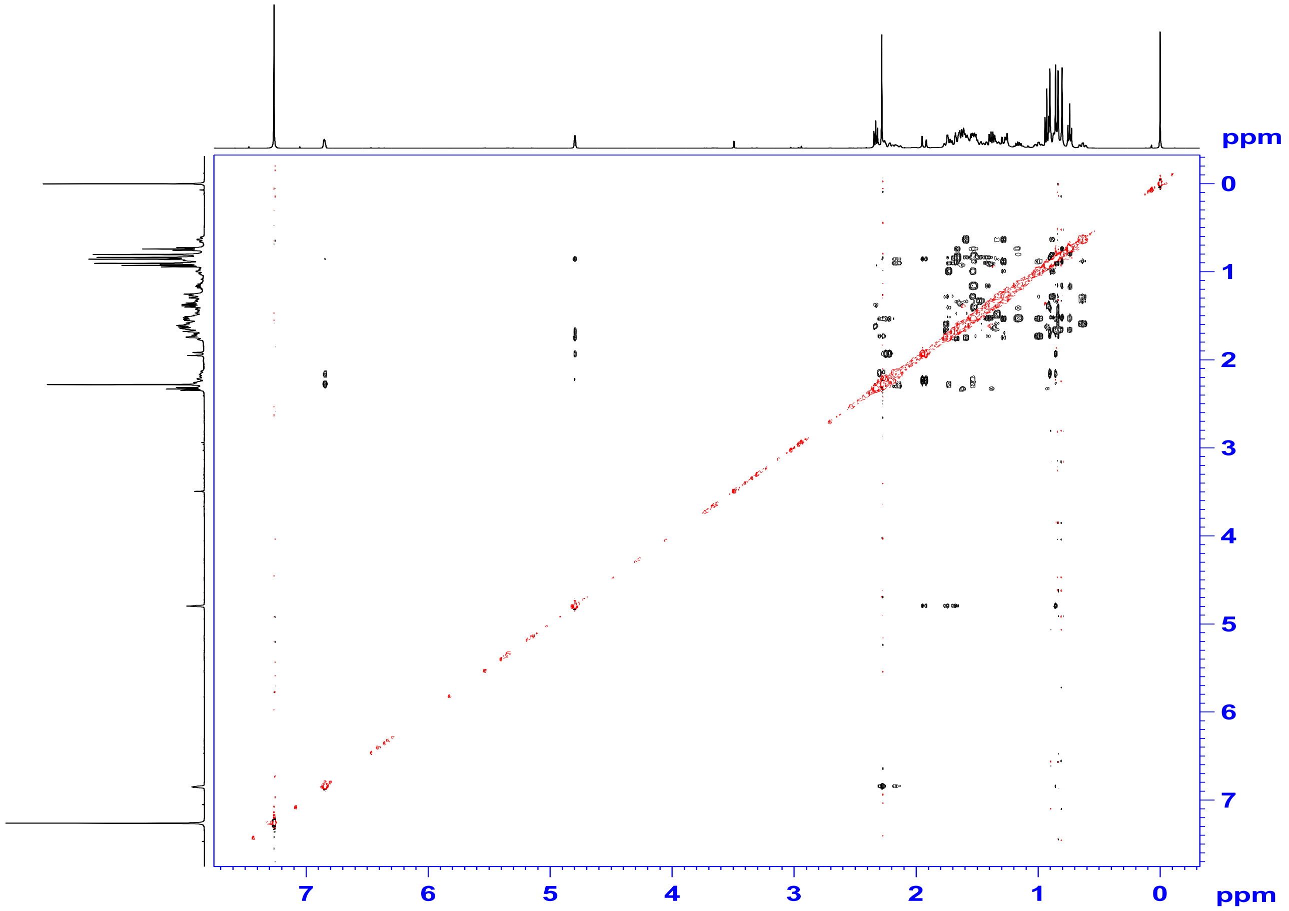

S12. HRESIMS of phyllofenone F (**1**).

Spectrum

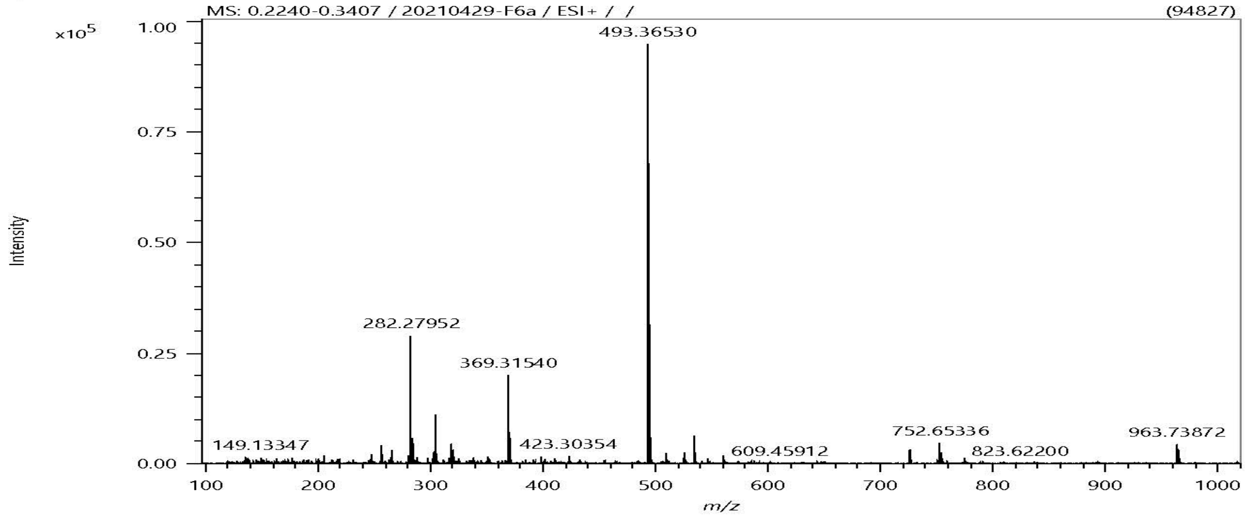

Elemental Composition

Parameters	
Tolerance:	±5.00 ppm
Electron:	Odd/Even
Charge:	+1
DBE:	-1.5 - 200.0

Elements Set 1:

Symbol	C	H	N	O	Na	S	Cl	Br
Min	0	0	0	0	1	0	0	0
Max	200	200	0	8	1	0	0	0

Symbol	F	P	Si
Min	0	0	0
Max	0	0	0

Results

Mass	Intensity	Intensity [%]	Formula	Calculated Mass	Mass Difference [mDa]	Mass Difference [ppm]	DBE
493.36530	94827.45	100.00	C31 H50 O3 Na	493.36522	0.08	0.17	6.5

S13. UV spectrum of phyllofenone F (**1**).

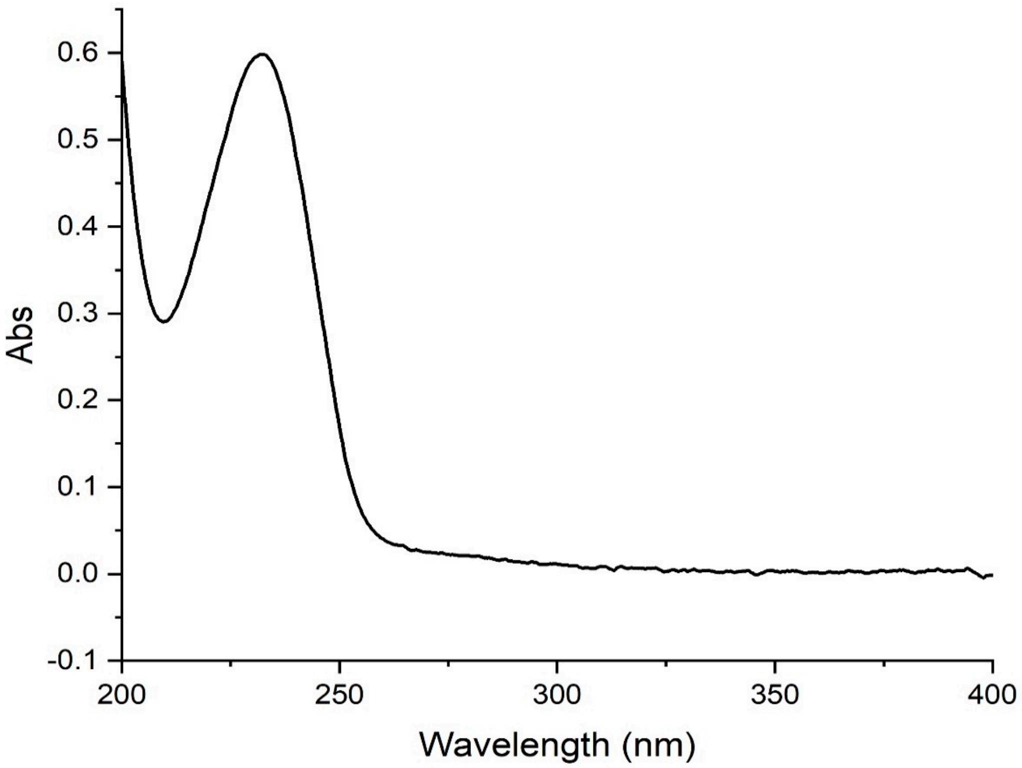

S24. IR spectrum of phyllofenone F (**1**) (KBr).

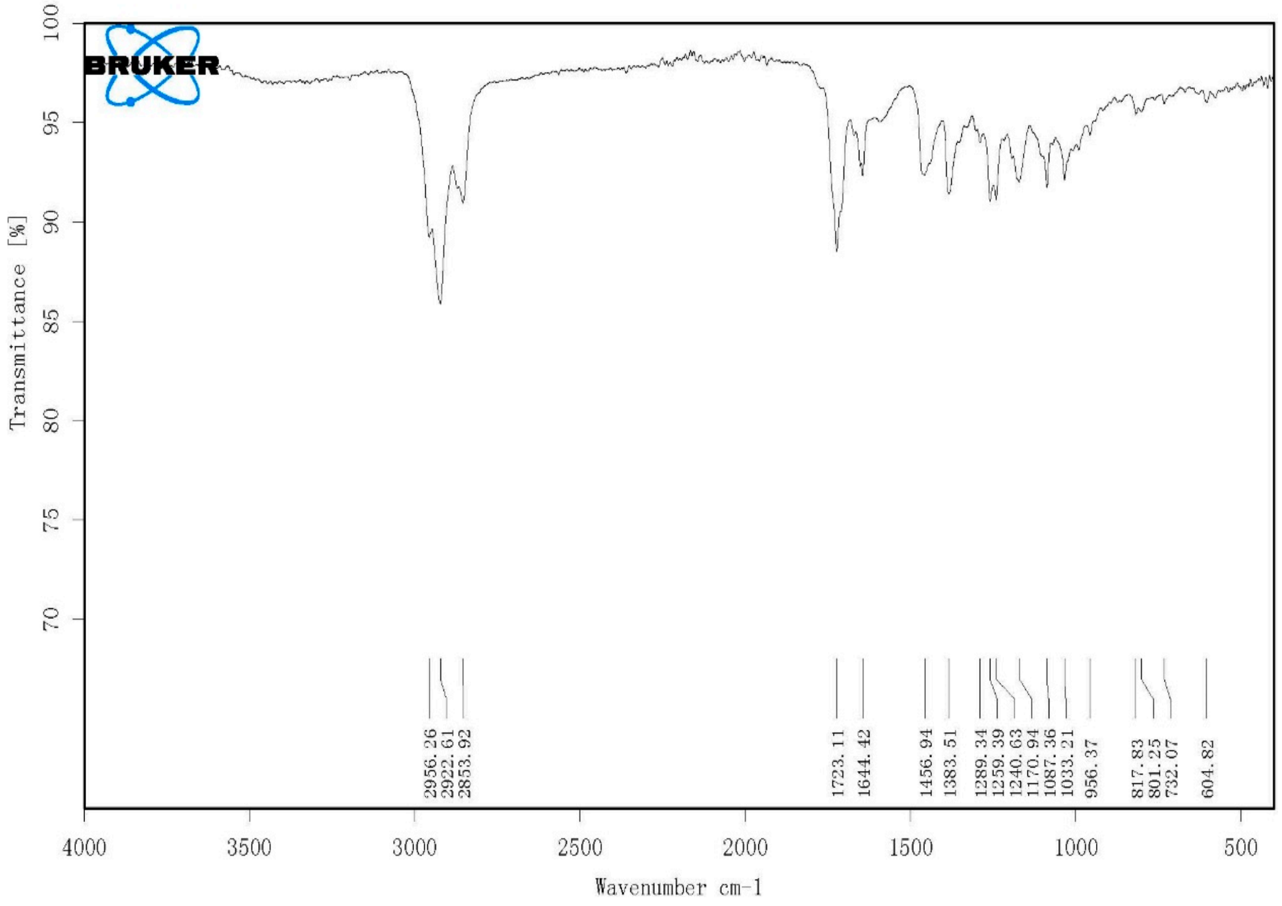

S15. ^{1}H NMR spectrum of phyllofenone G (**2**) in $CDCl_3$.

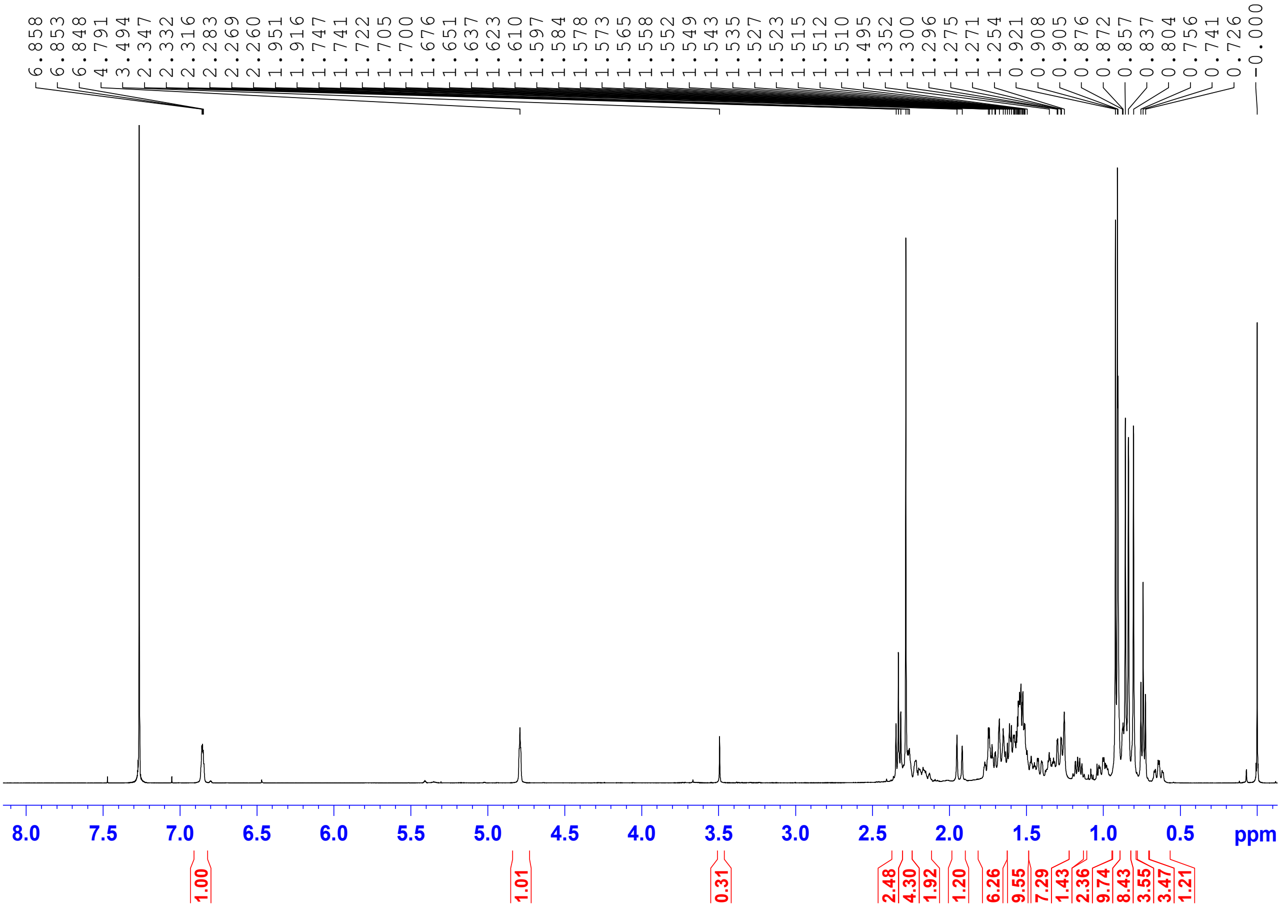

S16. ^{13}C NMR spectrum of phyllofenone G (**2**) in $CDCl_3$.

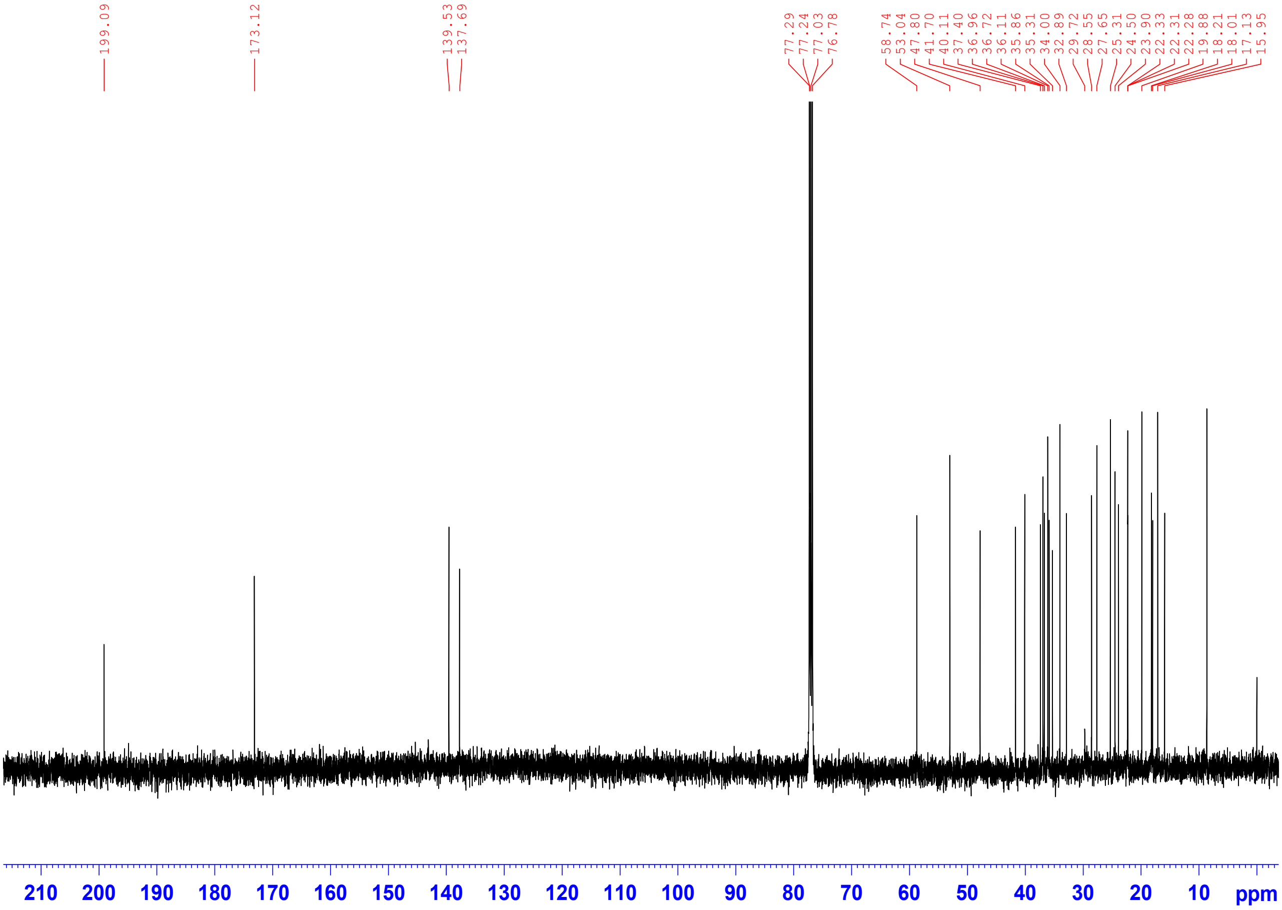

S17. DEPT135 spectrum of phyllofenone G (**2**) in $CDCl_3$.

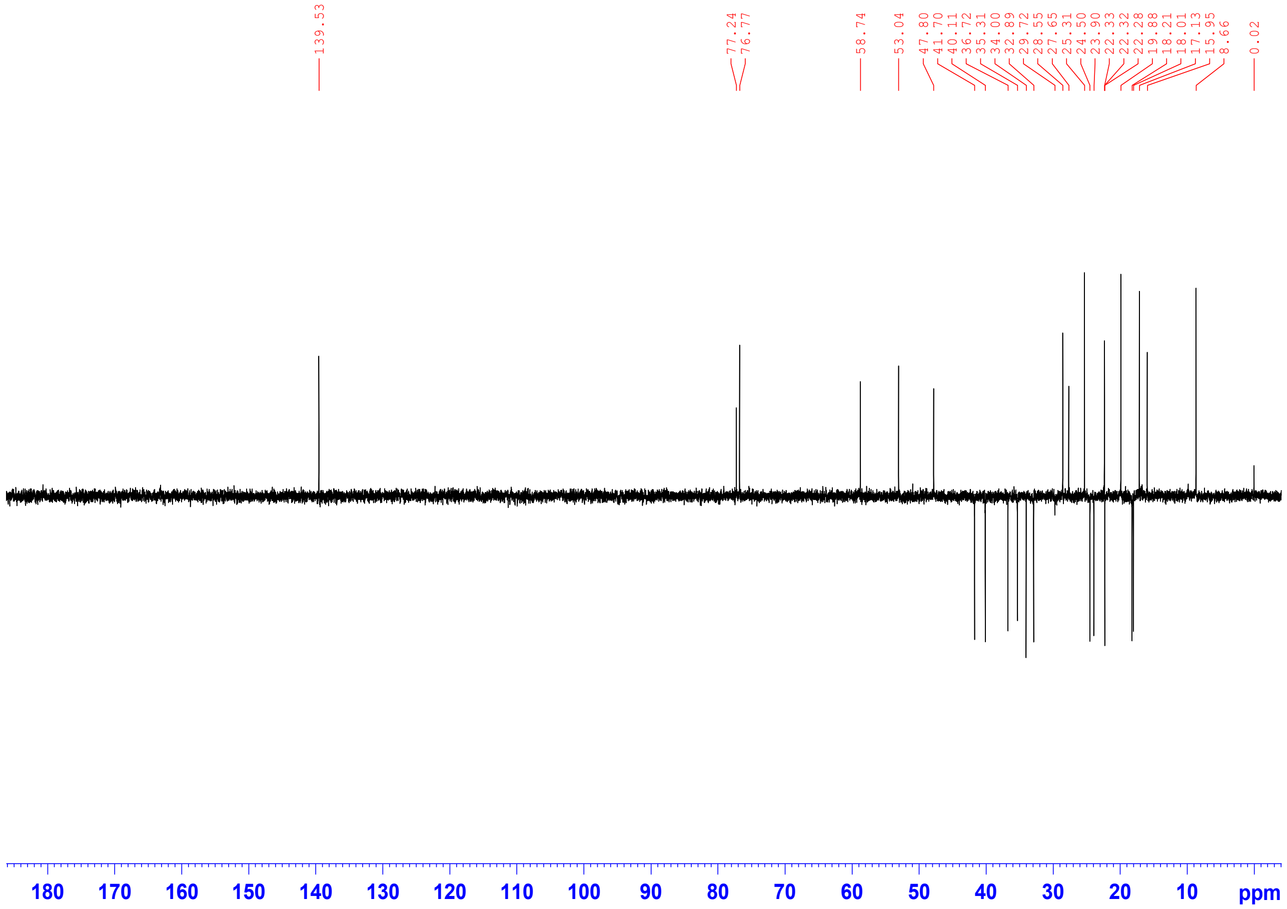

S18. HSQC spectrum of phyllofenone G (**2**) in $CDCl_3$.

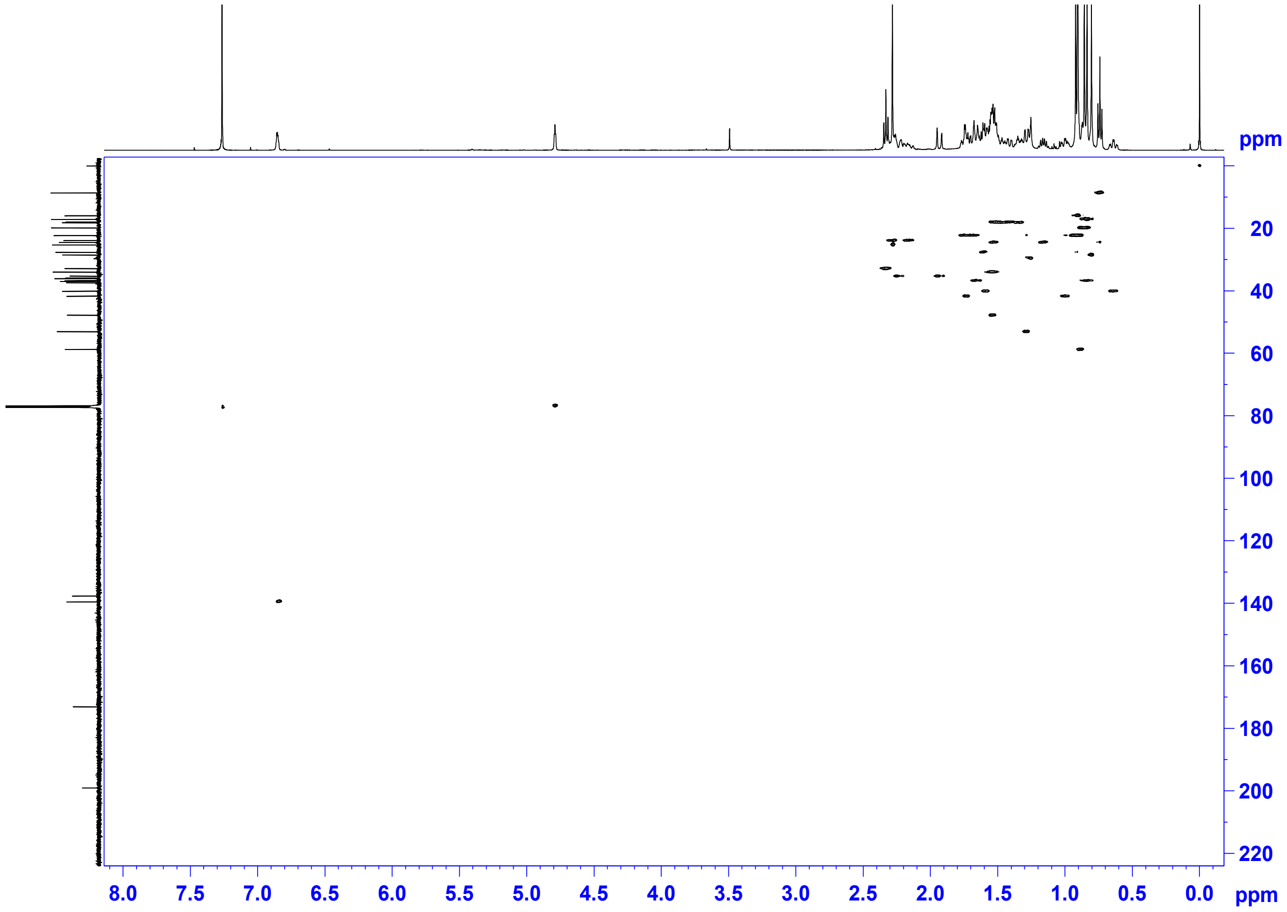

S19. COSY spectrum of phyllofenone G (**2**) in $CDCl_3$.

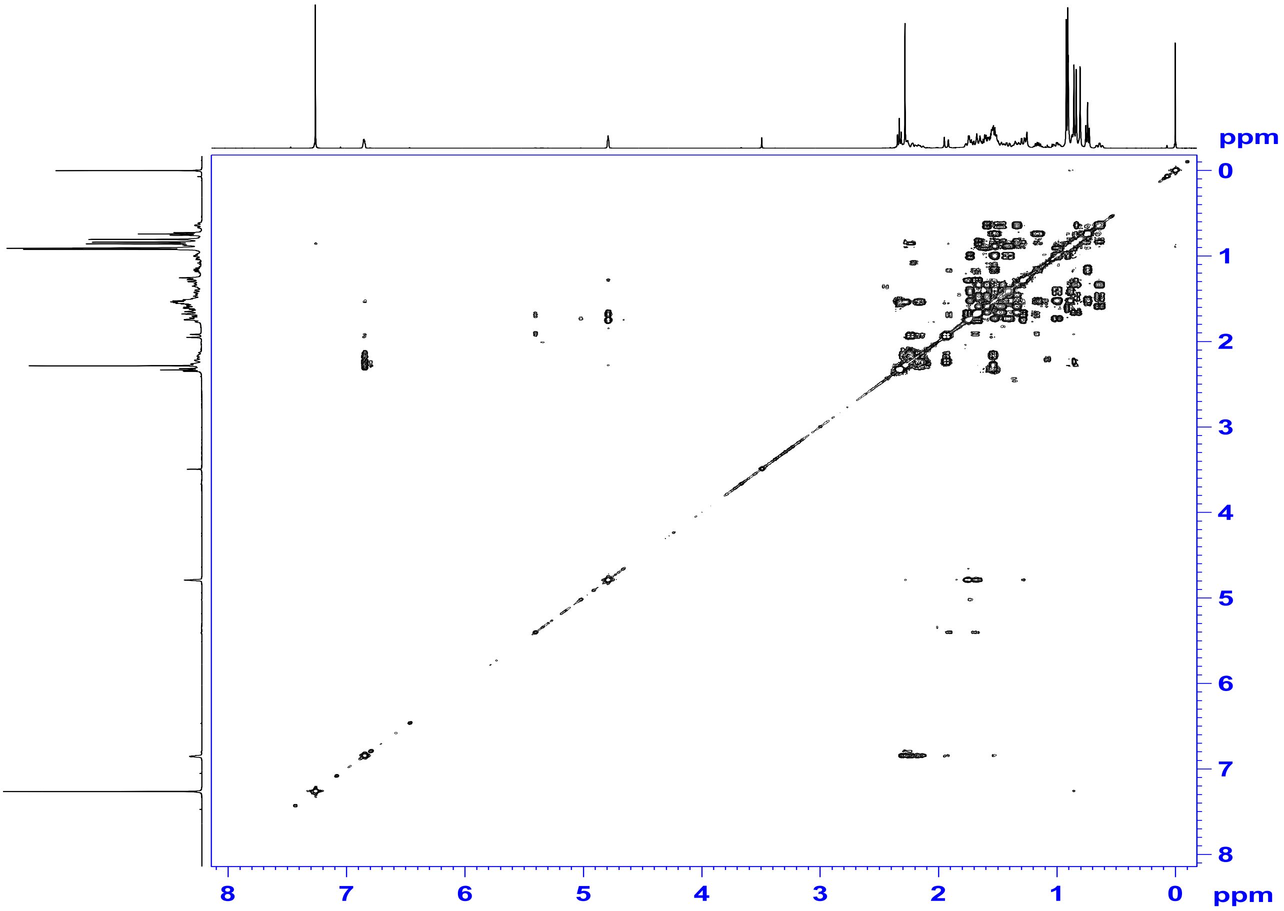

S20. HMBC spectrum of phyllofenone G (**2**) in $CDCl_3$.

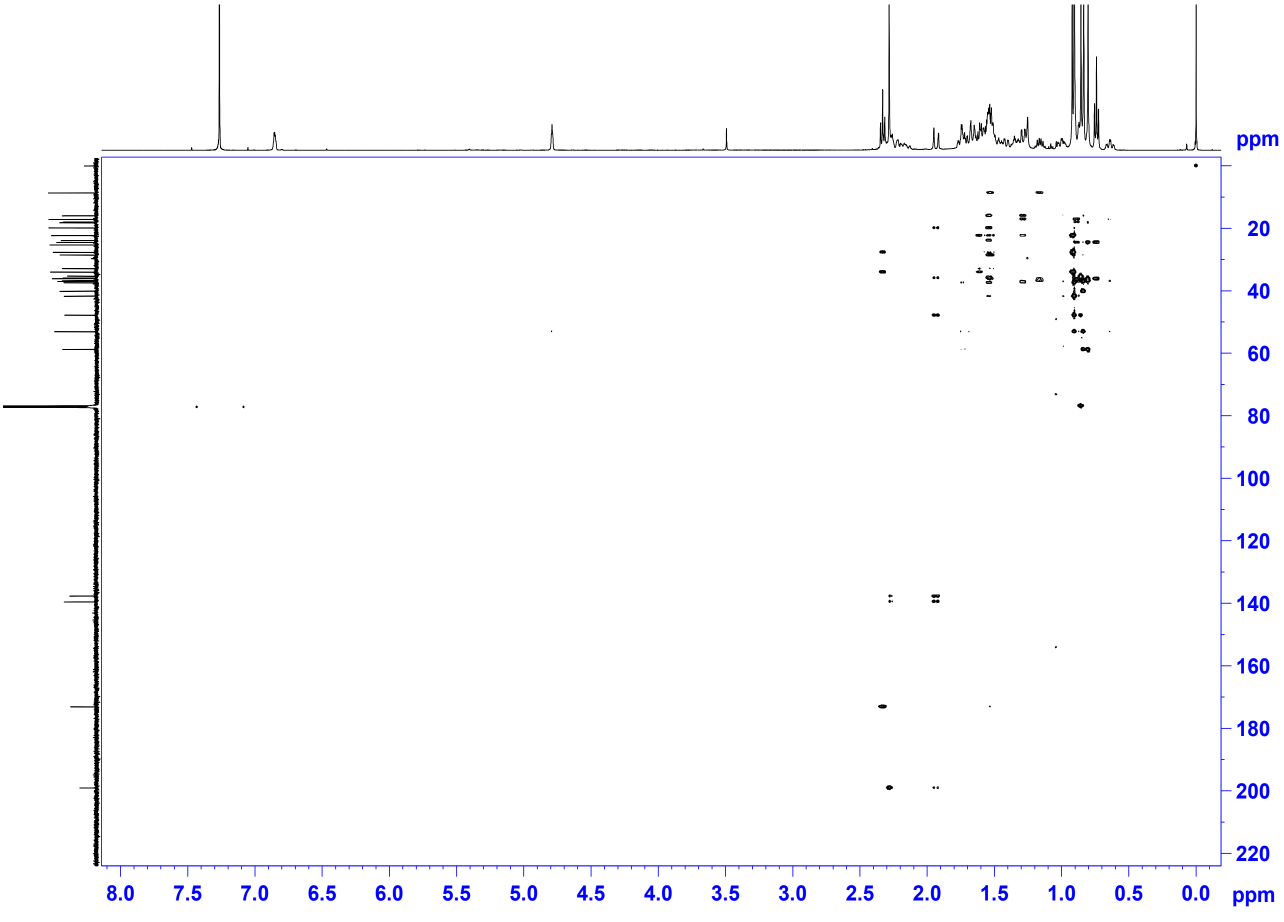

S21. NOESY spectrum of phyllofenone G (**2**) in $CDCl_3$.

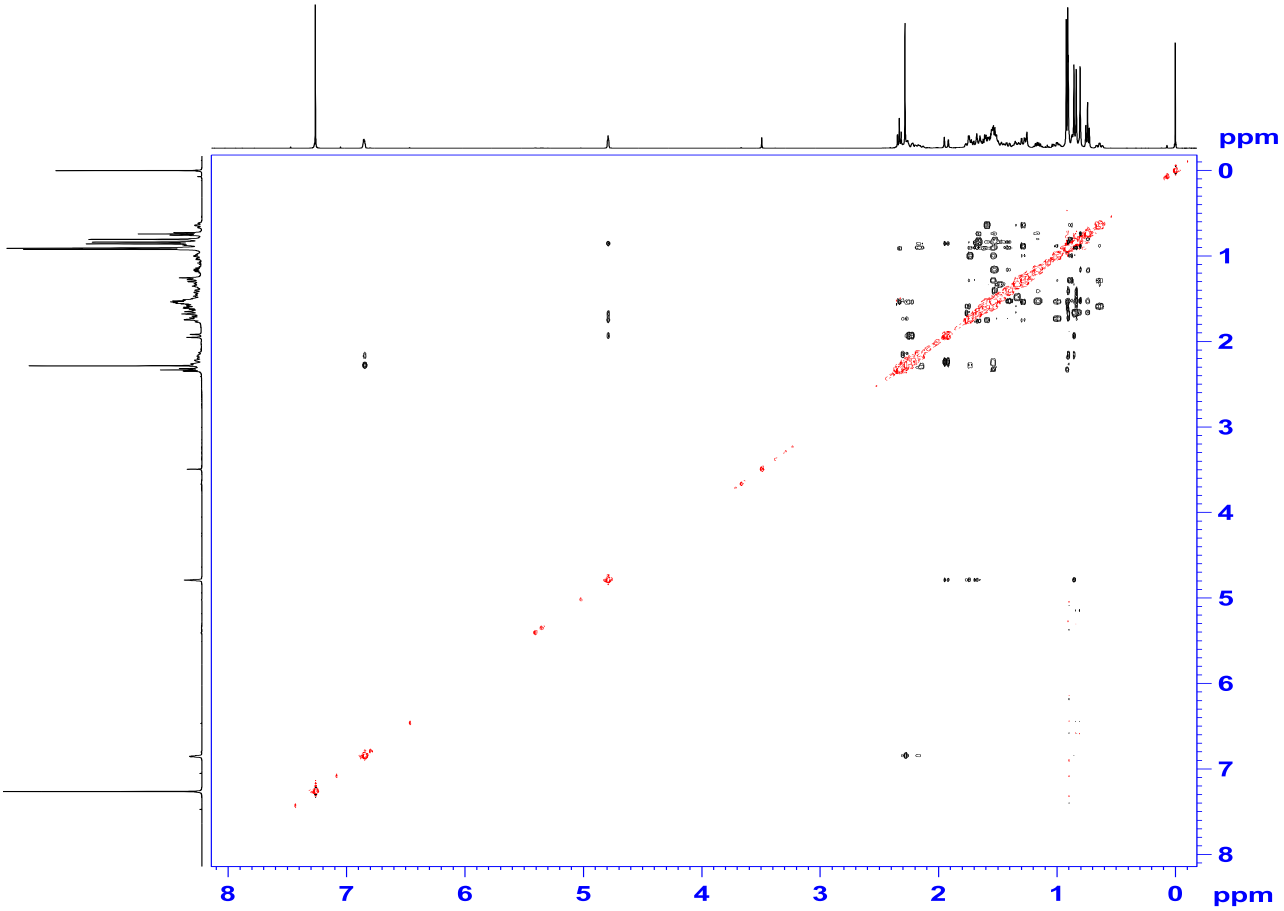

S22. HRESIMS of phyllofenone G (**2**).

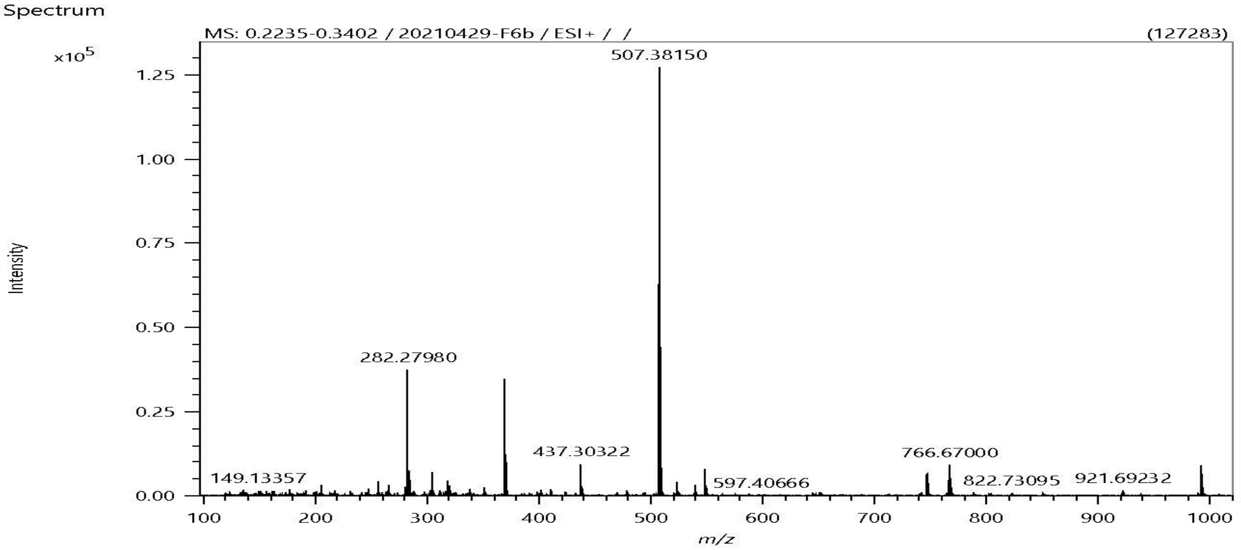

Elemental Composition

Parameters

Tolerance:	±5.00 ppm
Electron:	Odd/Even
Charge:	+1
DBE:	-1.5 - 200.0

Elements Set 1:

Symbol	C	H	N	O	Na	S	Cl	Br
Min	0	0	0	0	1	0	0	0
Max	200	200	0	8	1	0	0	0

Symbol	F	P	Si
Min	0	0	0
Max	0	0	0

Results

Mass	Intensity	Intensity [%]	Formula	Calculated Mass	Mass Difference [mDa]	Mass Difference [ppm]	DBE
507.38150	127282.54	100.00	C32 H52 O3 Na	507.38087	0.63	1.24	6.5

S23. UV spectrum of phyllofenone G (**2**) in MeOH.

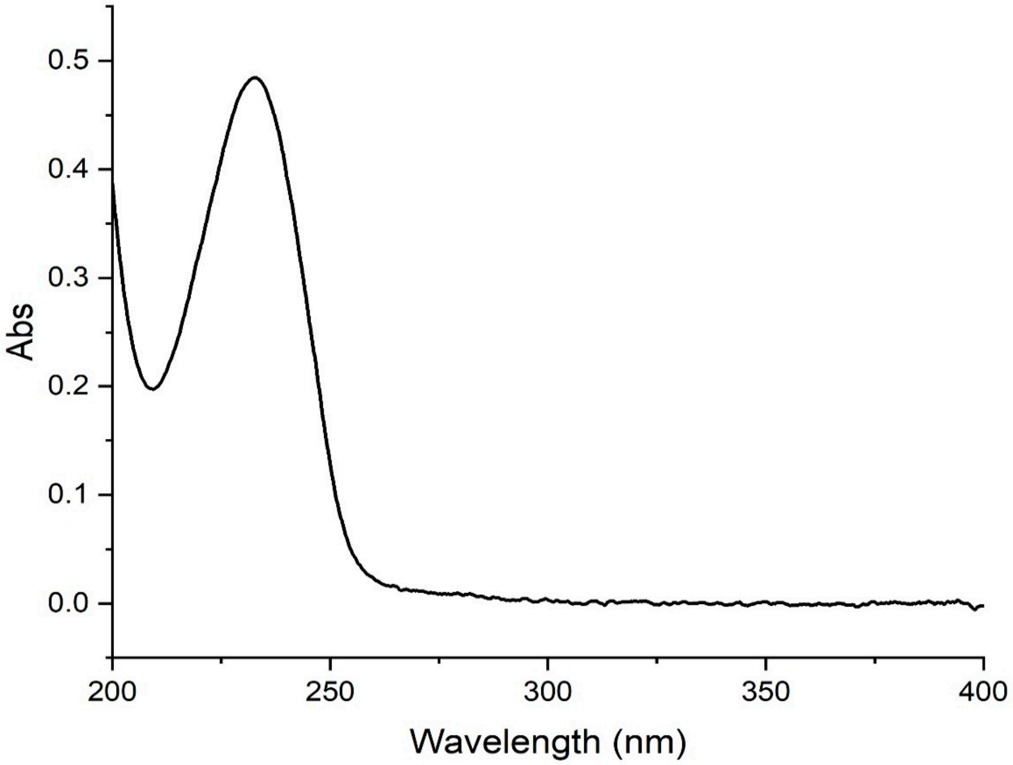

S24 IR spectrum of phyllofenone G (**2**) (KBr).

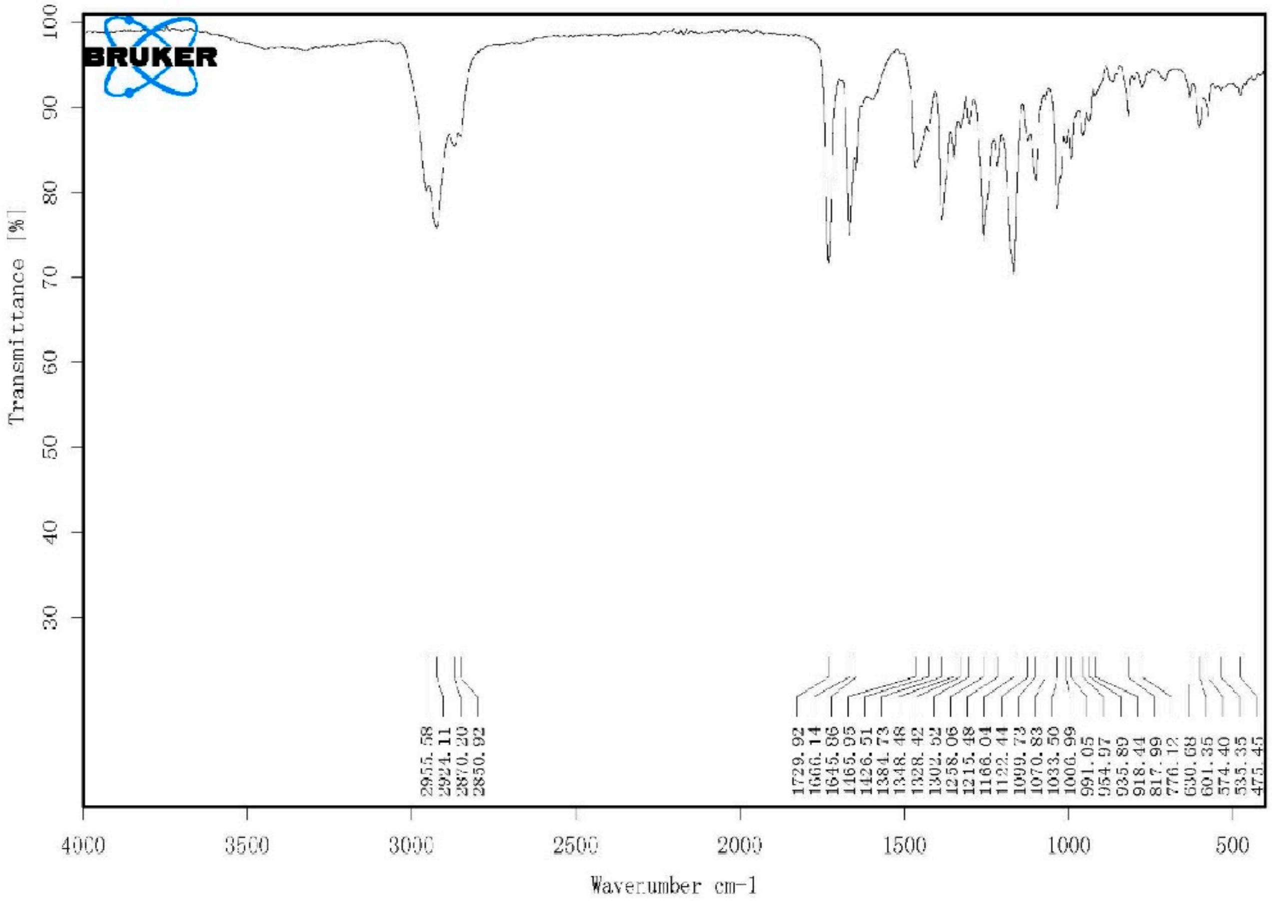

S25. ^{1}H NMR spectrum of phyllofenone H (**3**) in $CDCl_3$.

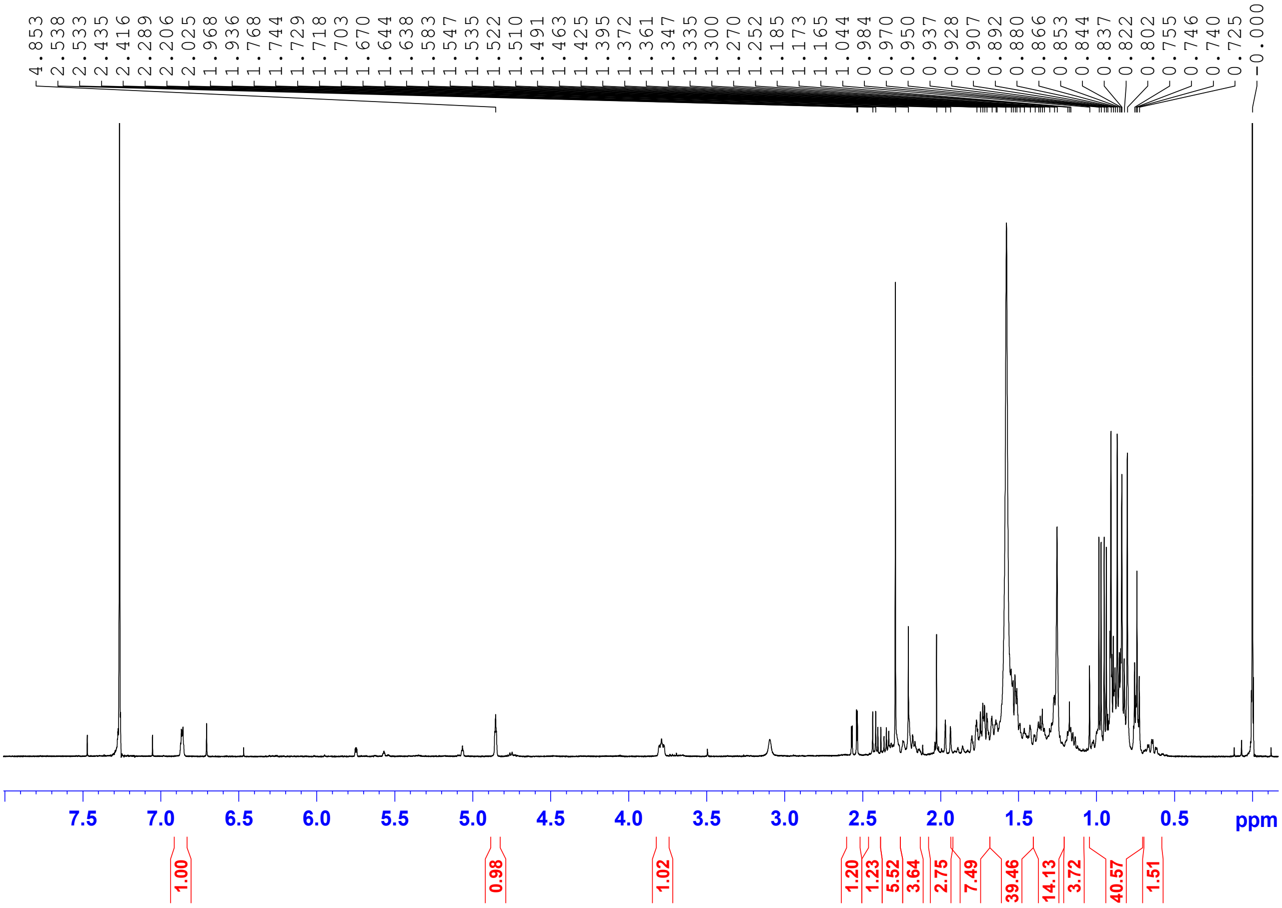

S26. ^{13}C NMR spectrum of phyllofenone H (**3**) in $CDCl_3$.

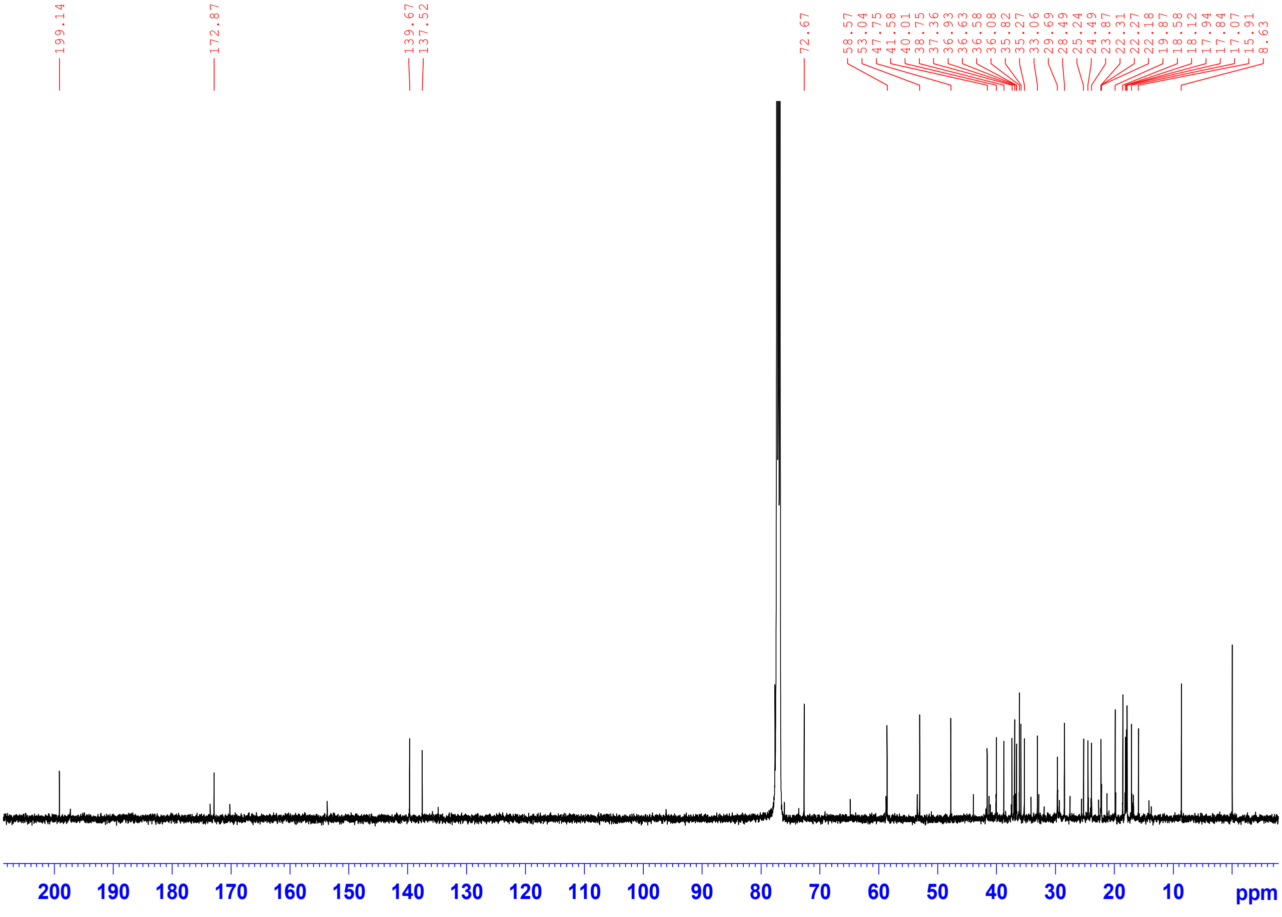

S27. DEPT135 spectrum of phyllofenone H (**3**) in CDCl3.

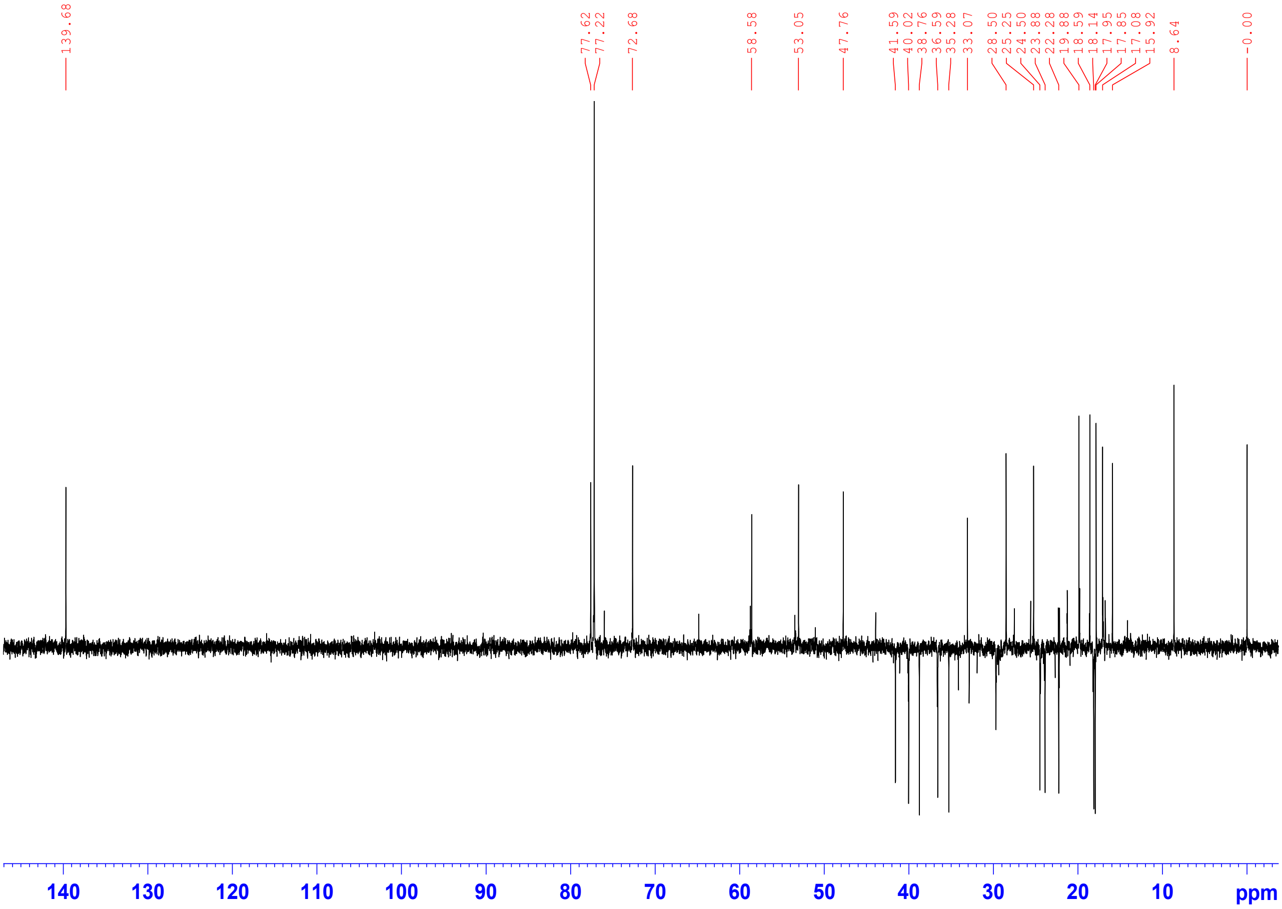

S28. HSQC spectrum of phyllofenone H (**3**) in CDCl3.

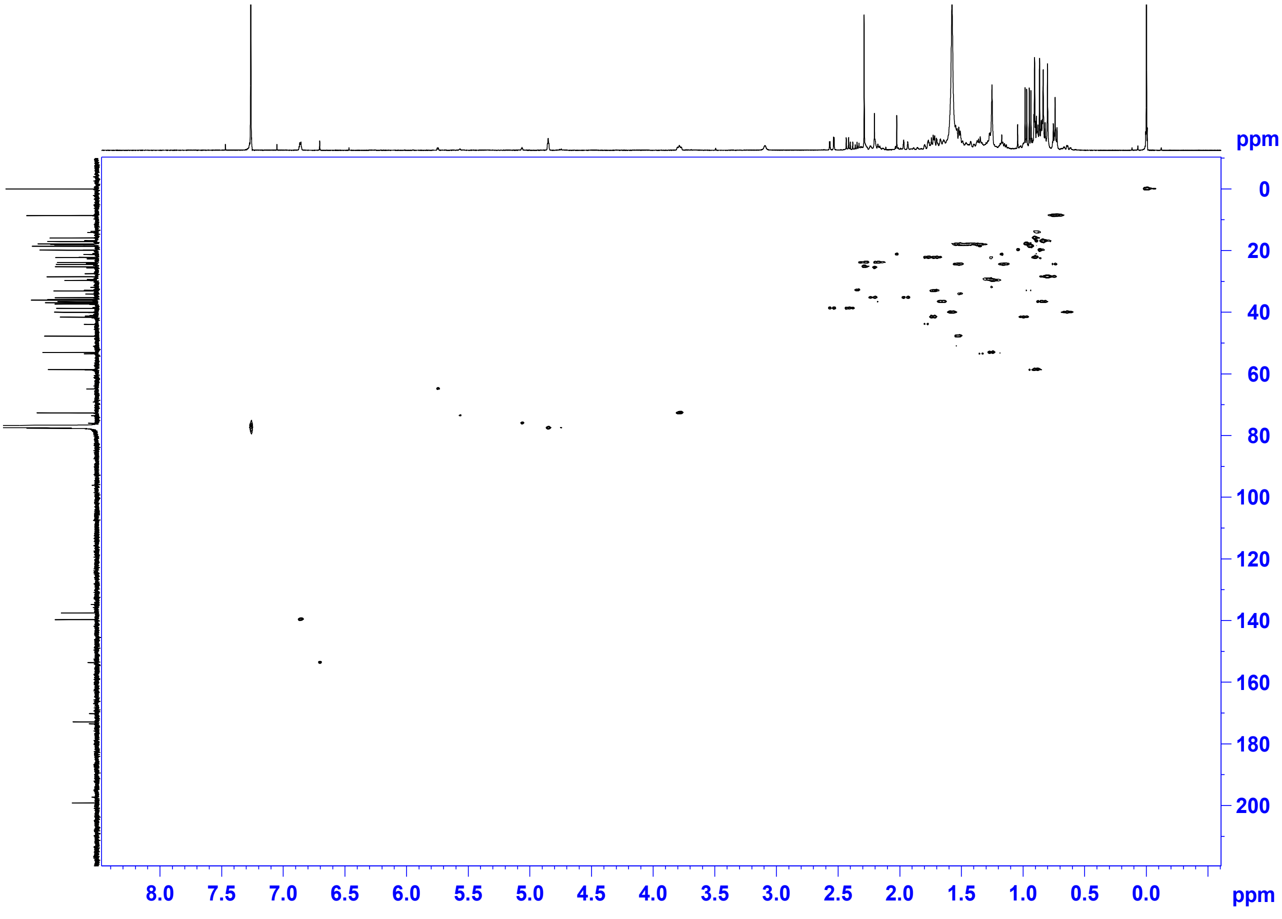

S29. COSY spectrum of phyllofenone H (**3**) in CDCl3.

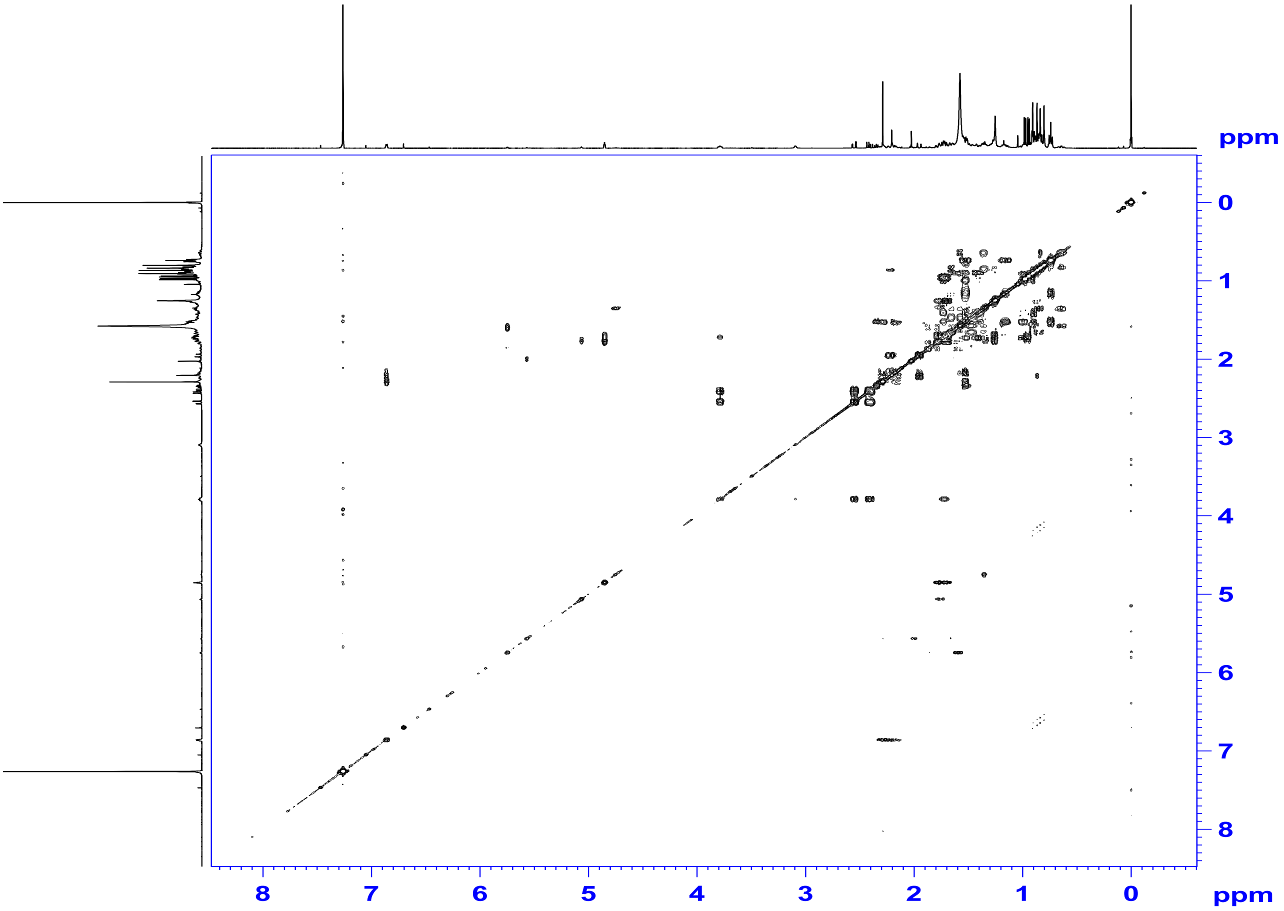

S30. HMBC spectrum of phyllofenone H (**3**) in CDCl3.

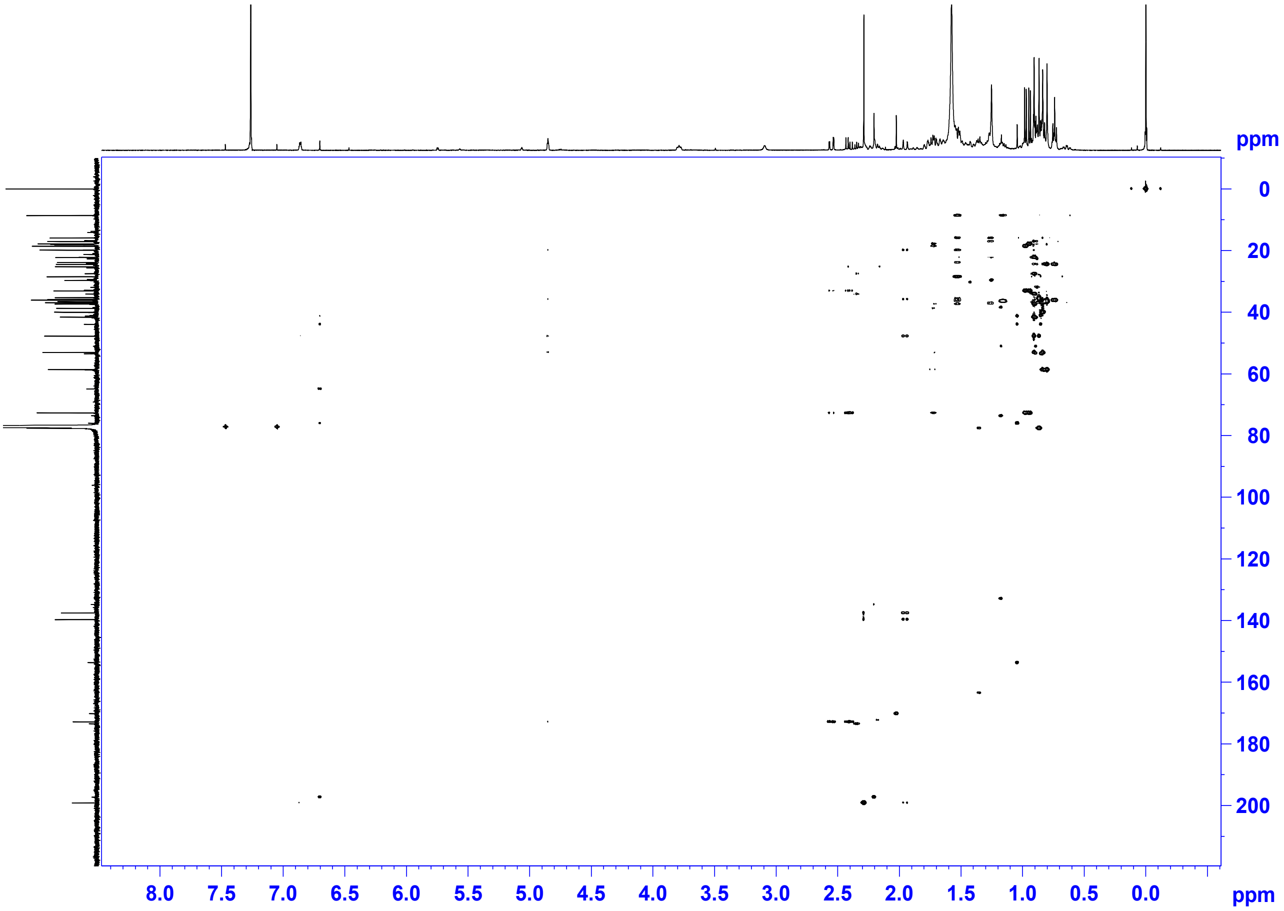

S31. NOESY spectrum of phyllofenone H (**3**) in $CDCl_3$.

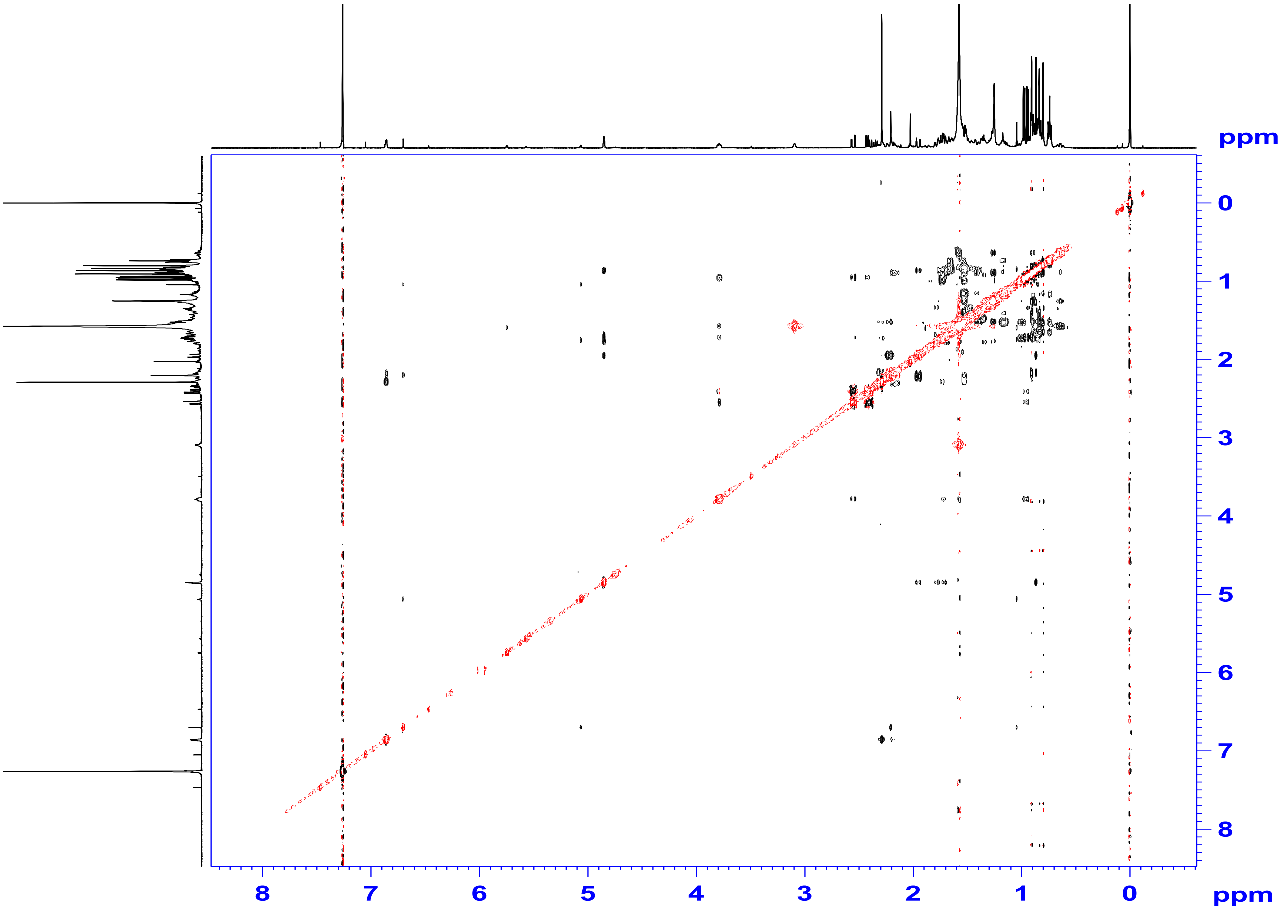

S32. HRESIMS of phyllofenone H (**3**).

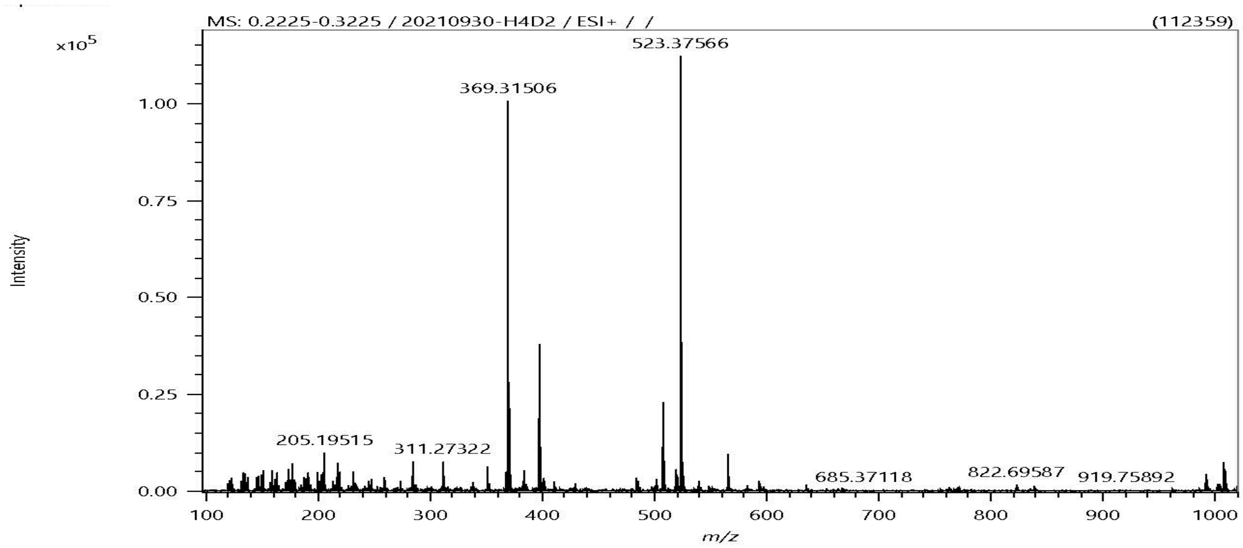

Elemental Composition

Parameters

Tolerance:	±5.00 ppm
Electron:	Odd/Even
Charge:	+1
DBE:	-1.5 - 200.0

Elements Set 1:

Symbol	C	H	N	O	Na	S	Cl	Br
Min	0	0	0	0	1	0	0	0
Max	200	200	0	8	1	0	0	0

Symbol	P	Si	F	I
Min	0	0	0	0
Max	0	0	0	0

Results

Mass	Intensity	Intensity [%]	Formula	Calculated Mass	Mass Difference [mDa]	Mass Difference [ppm]	DBE
523.37566	112359.39	100.00	C32 H52 O4 Na	523.37578	-0.12	-0.23	6.5

S33. UV spectrum of phyllofenone H (**3**).

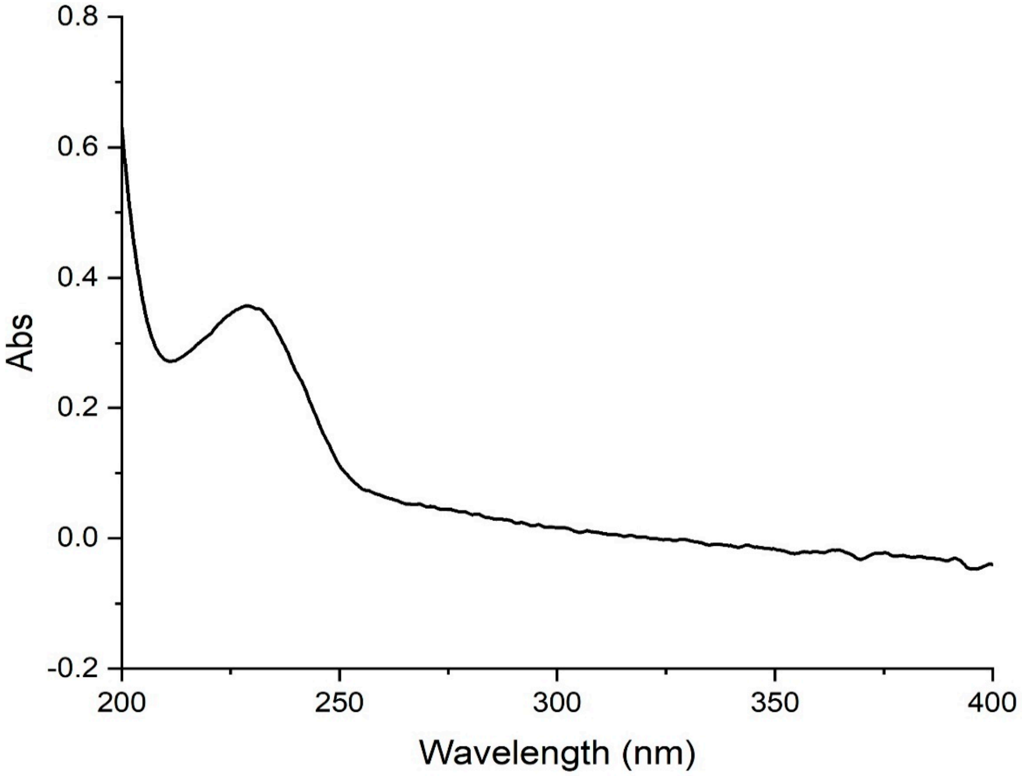

S34. IR spectrum of phyllofenone H (**3**) (KBr).

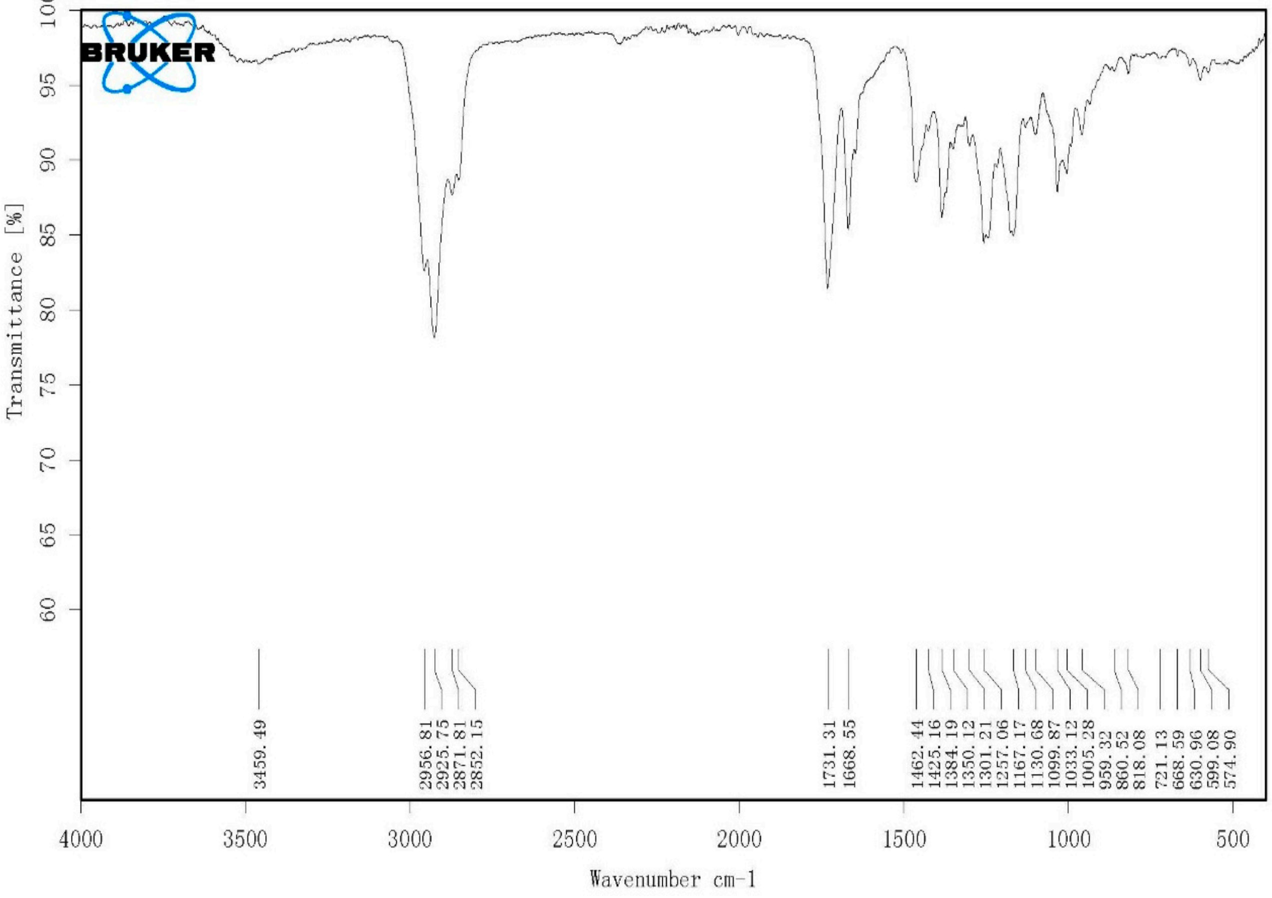

S35. ^{1}H NMR spectrum of phyllofenone I (**4**) in $CDCl_3$.

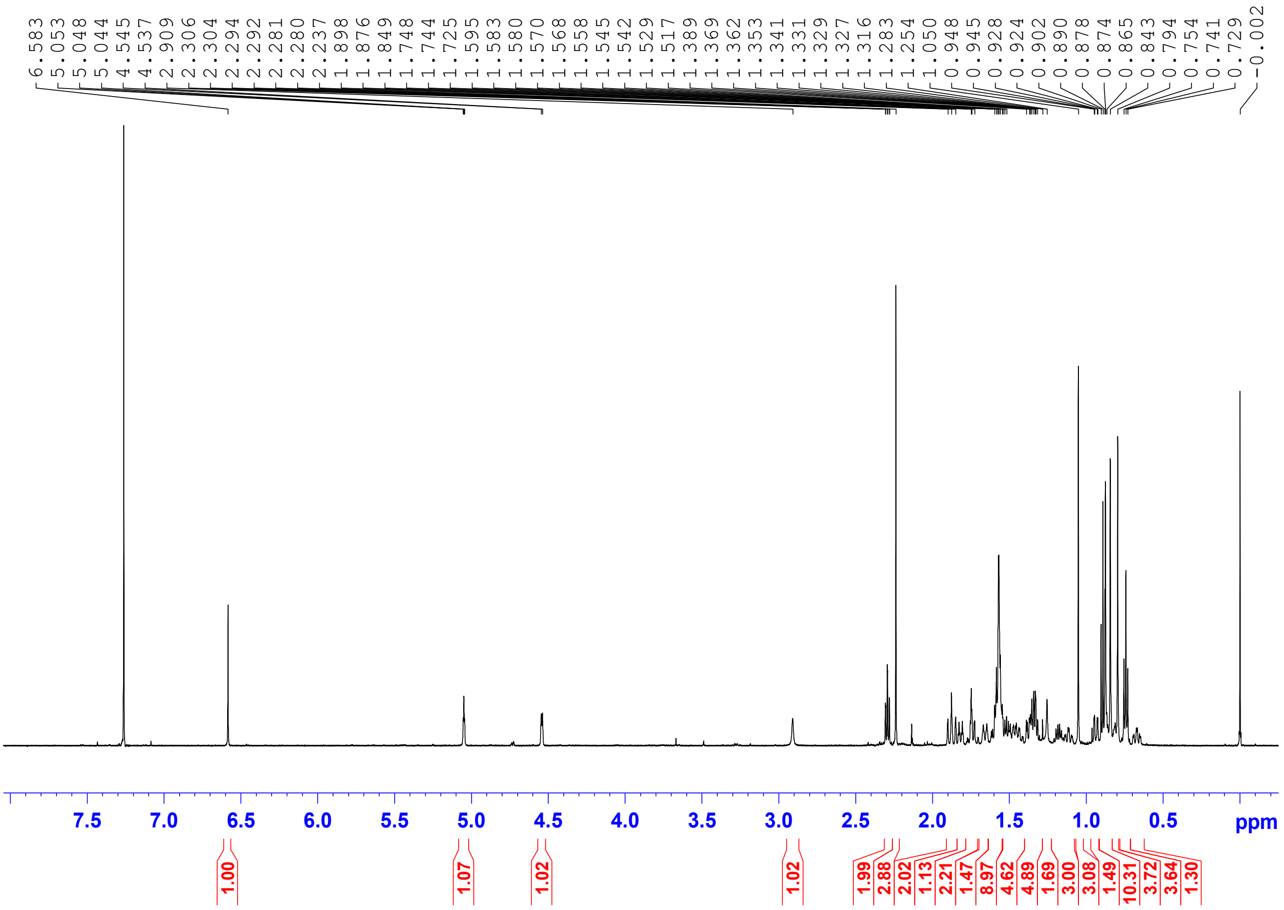

S36. ^{13}C NMR spectrum of phyllofenone I (**4**) in $CDCl_3$.

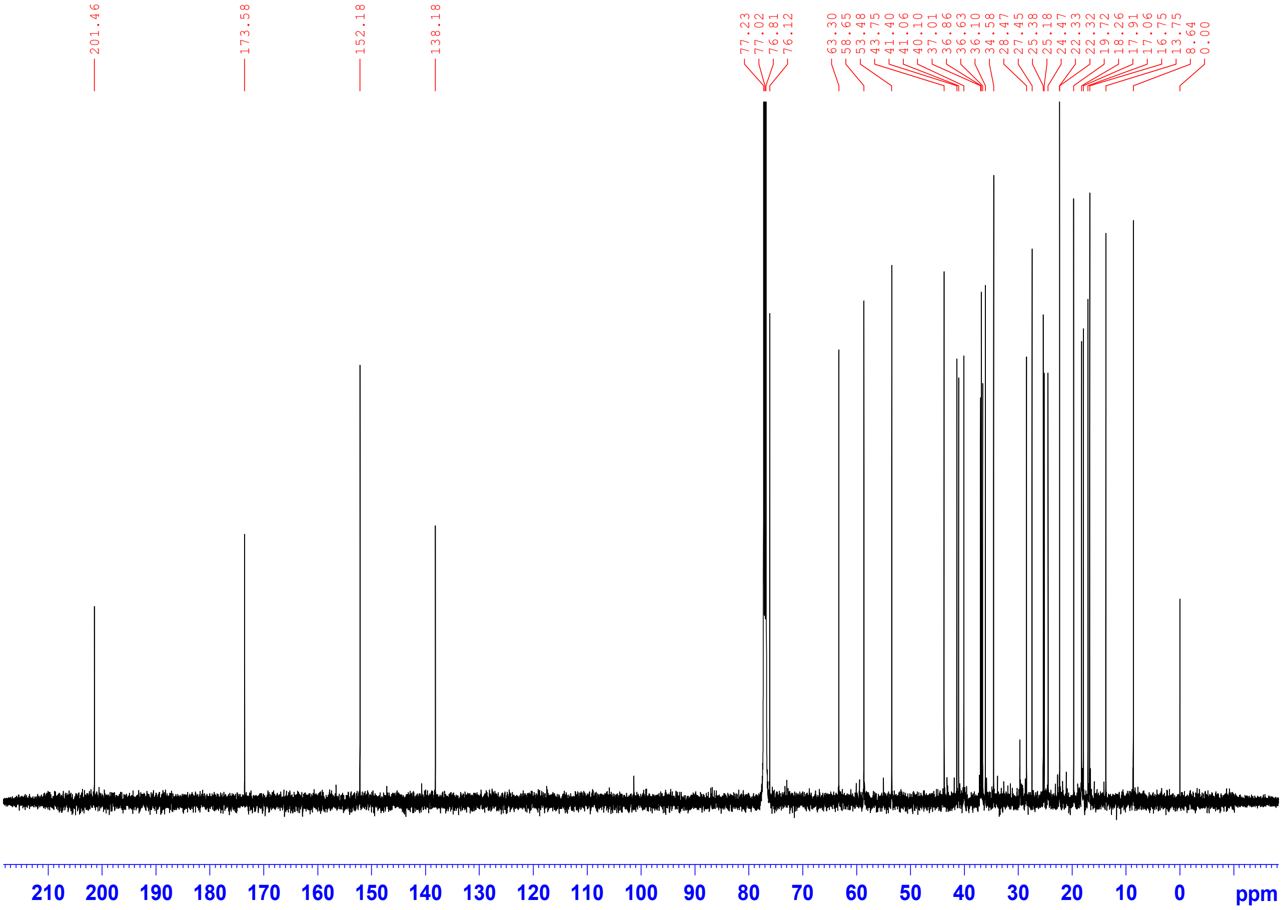

S37. DEPT135 spectrum of phyllofenone I (**4**) in $CDCl_3$.

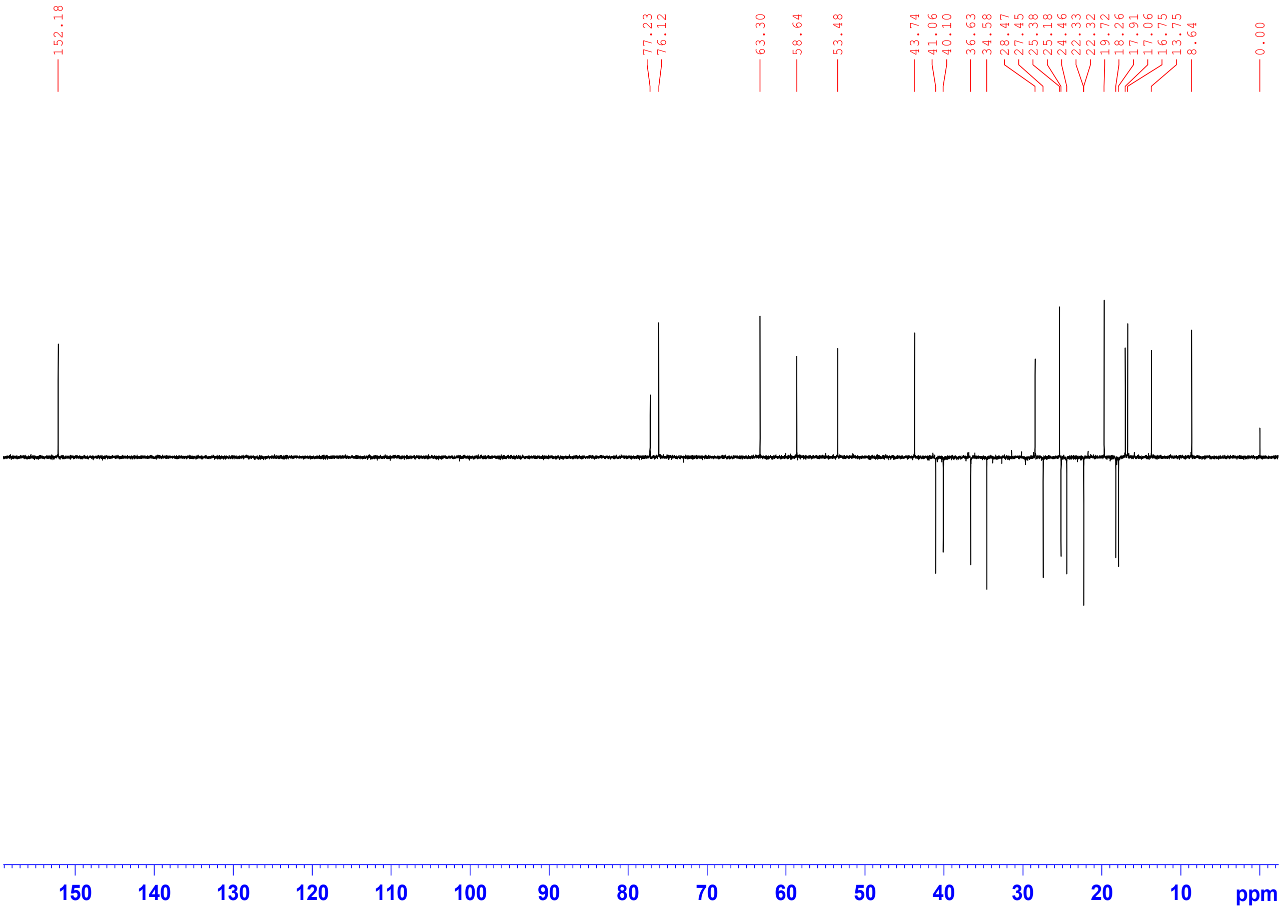

S38. HSQC spectrum of phyllofenone I (**4**) in $CDCl_3$.

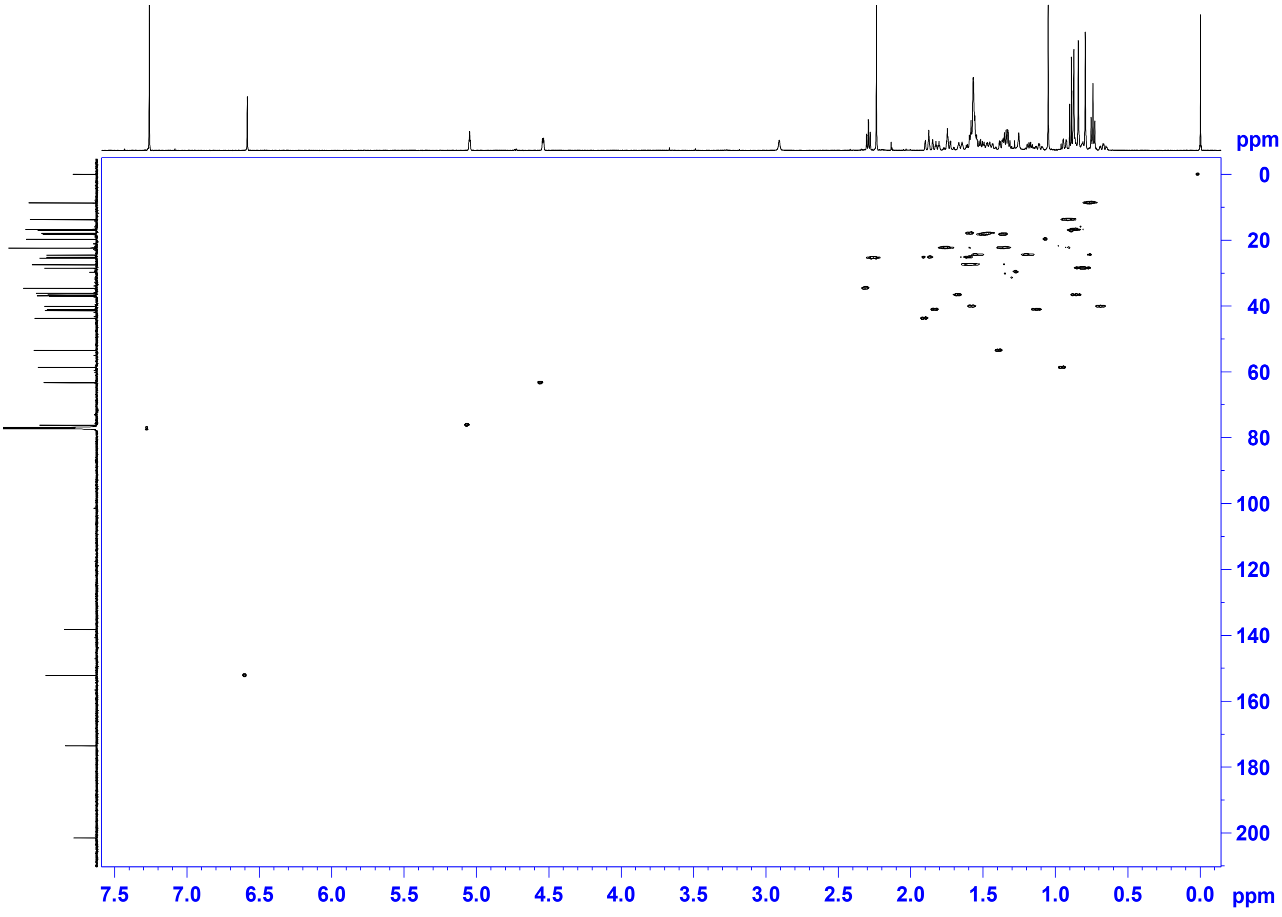

S39. COSY spectrum of phyllofenone I (**4**) in $CDCl_3$.

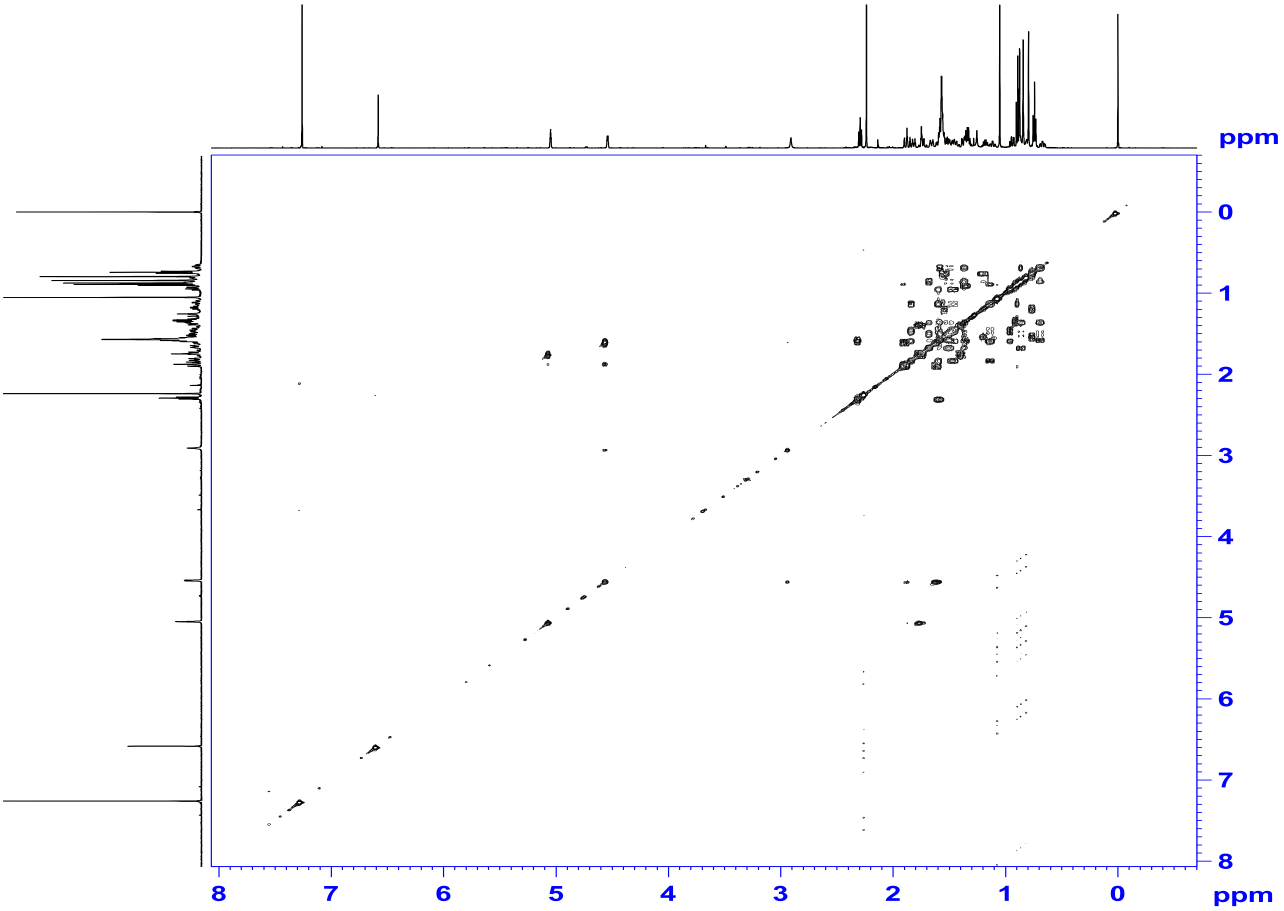

S40. HMBC spectrum of phyllofenone I (**4**) in $CDCl_3$.

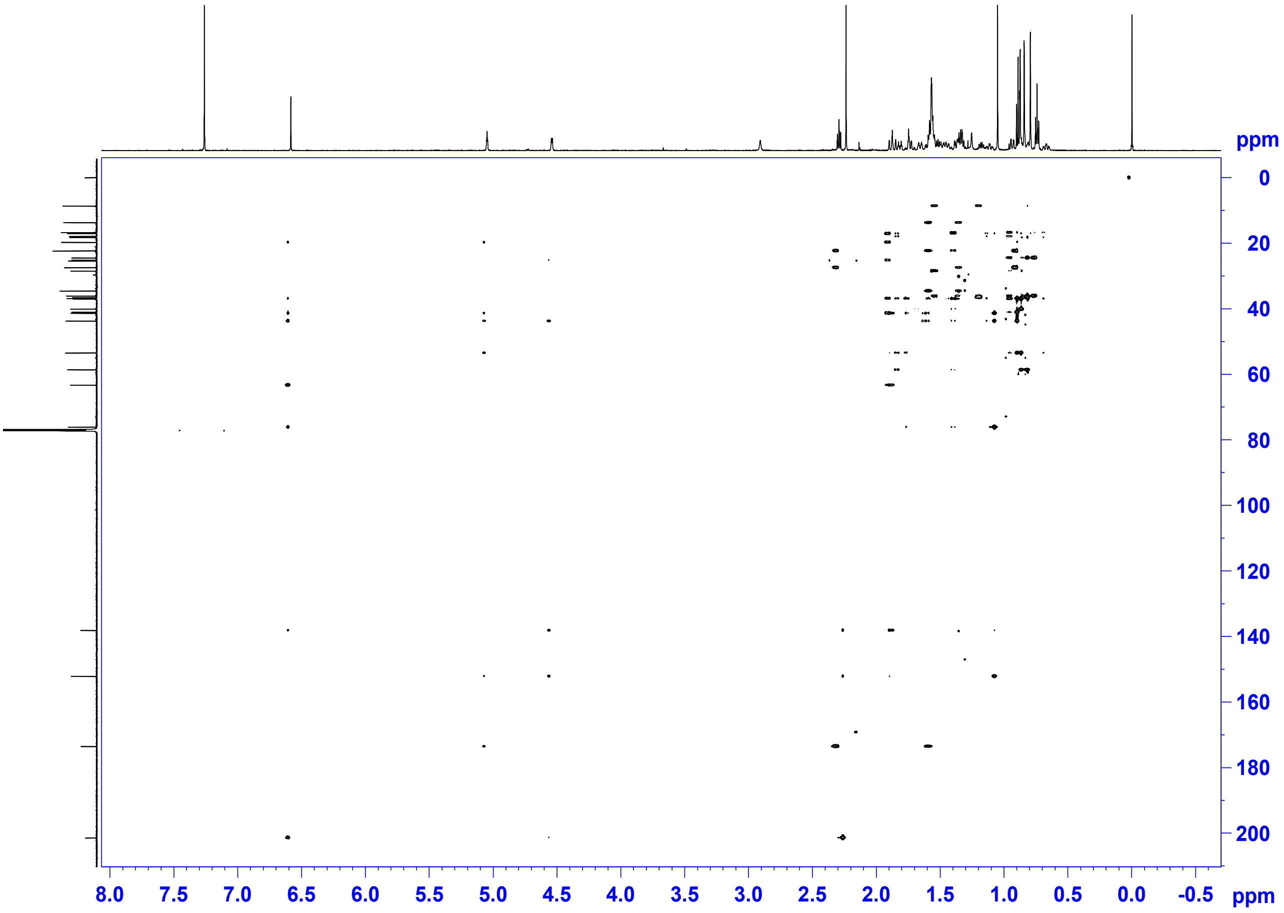

S41. NOESY spectrum of phyllofenone I (**4**) in $CDCl_3$.

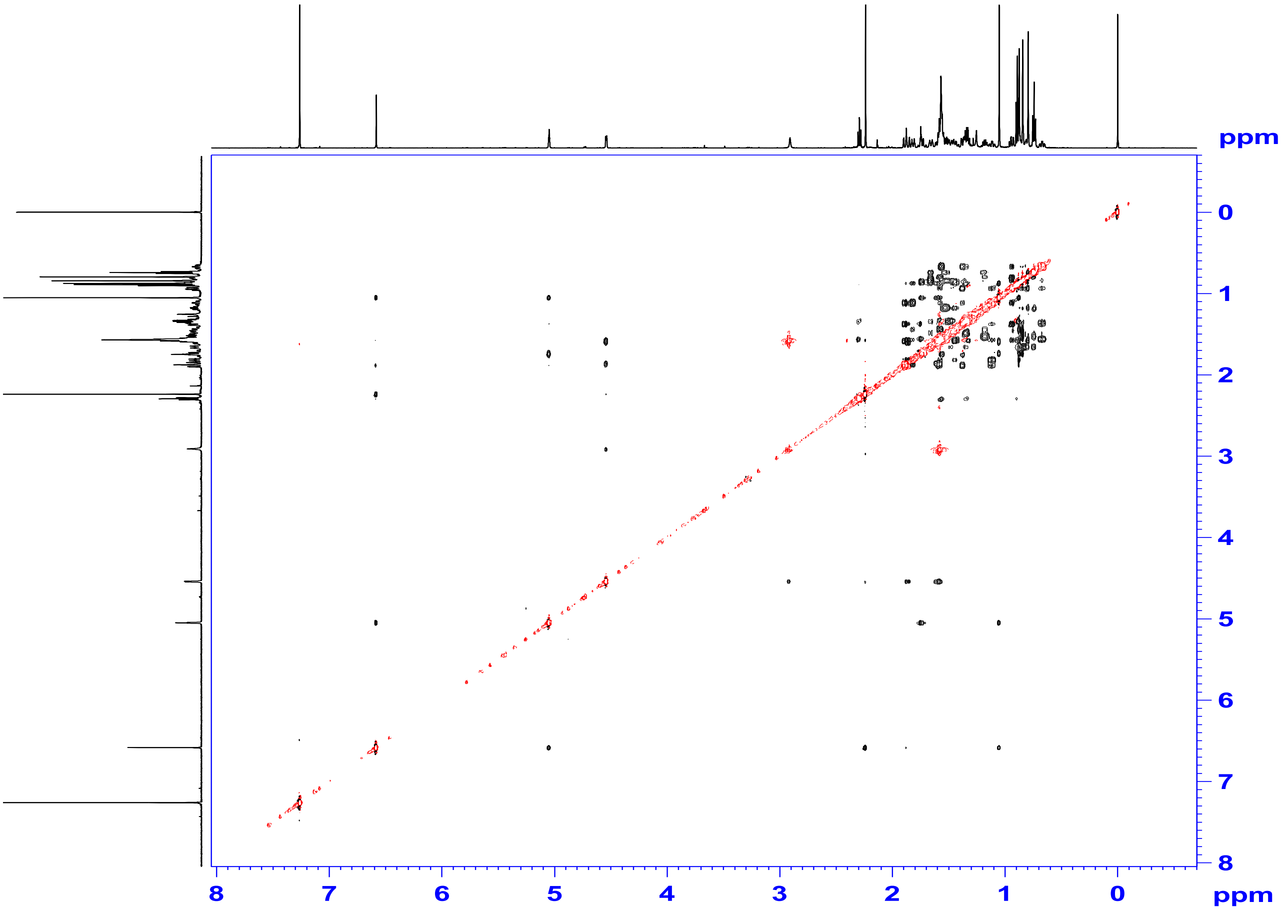

S42. HRESIMS of phyllofenone I (**4**).

Spectrum

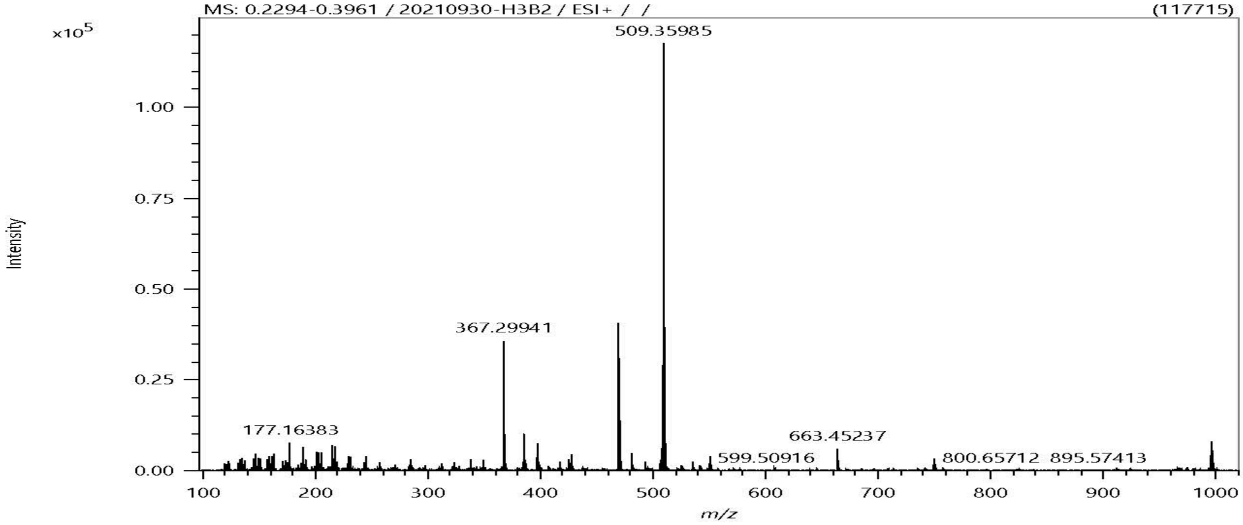

Elemental Composition

Parameters

Tolerance:	±5.00 ppm
Electron:	Odd/Even
Charge:	+1
DBE:	-1.5 - 200.0

Elements Set 1:

Symbol	C	H	N	O	Na	S	Cl	Br
Min	0	0	0	0	1	0	0	0
Max	200	200	0	8	1	0	0	0

Symbol	P	Si	F	I
Min	0	0	0	0
Max	0	0	0	0

Results

Mass	Intensity	Intensity [%]	Formula	Calculated Mass	Mass Difference [mDa]	Mass Difference [ppm]	DBE
509.35985	117715.40	100.00	C31 H50 O4 Na	509.36013	-0.28	-0.55	6.5

S43. UV spectrum of phyllofenone I (**4**).

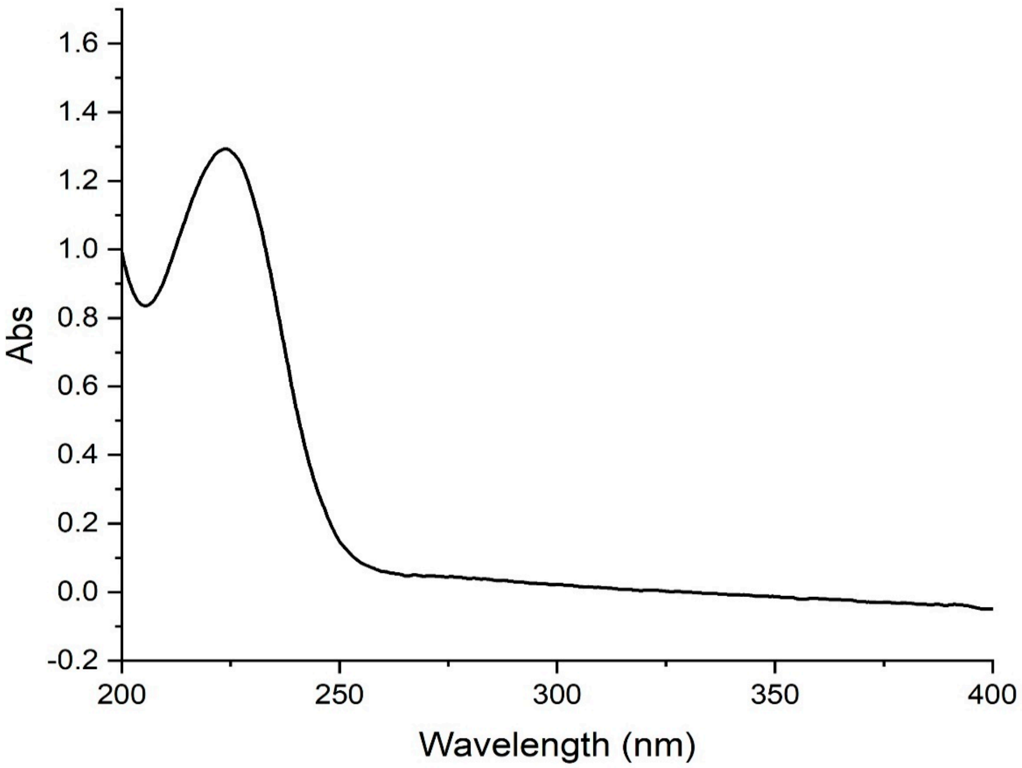

S44. IR spectrum of phyllofenone I (**4**) (KBr).

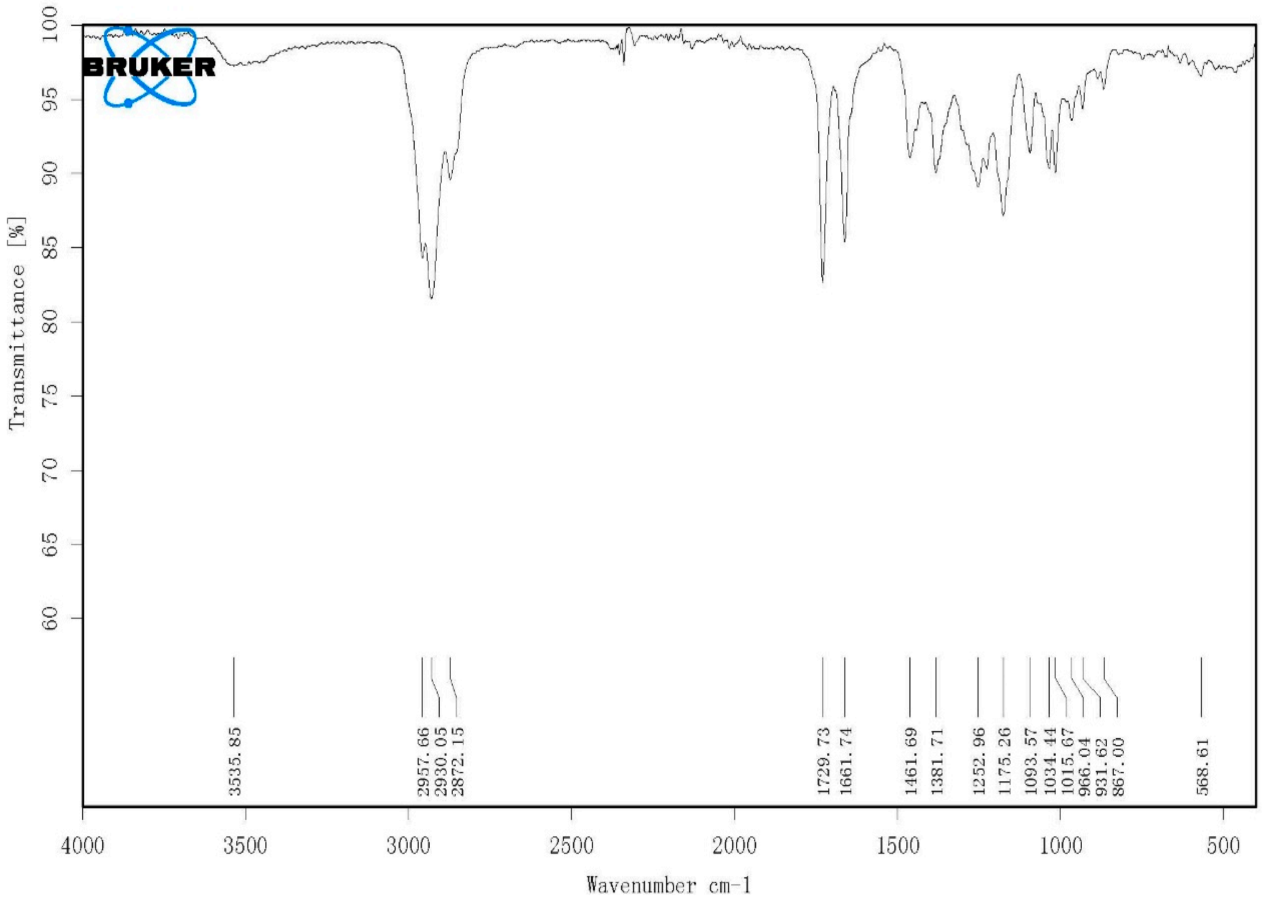

S45. ^{1}H NMR spectrum of phyllofenone J (**5**) in $CDCl_3$.

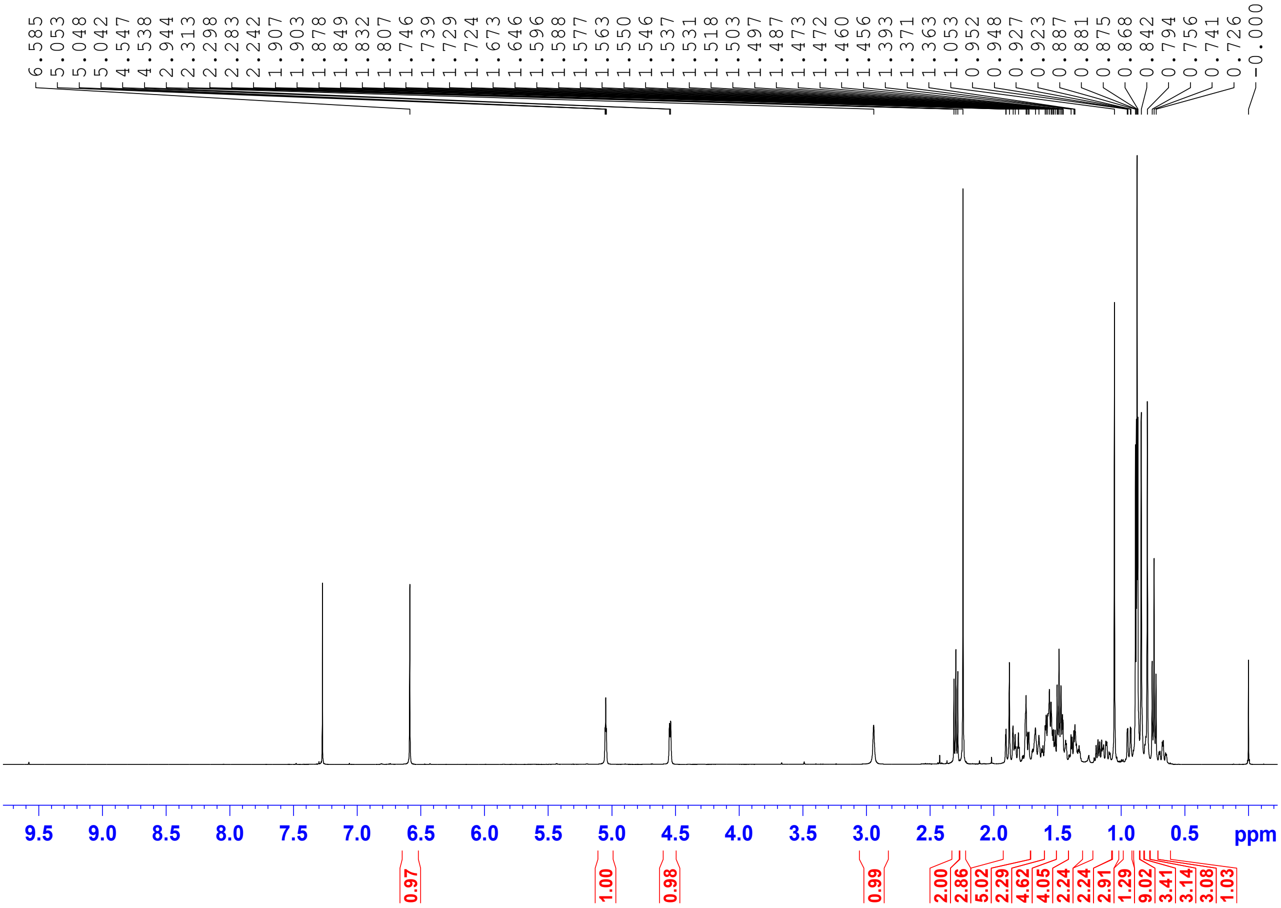

S46. ^{13}C NMR spectrum of phyllofenone J (**5**) in $CDCl_3$.

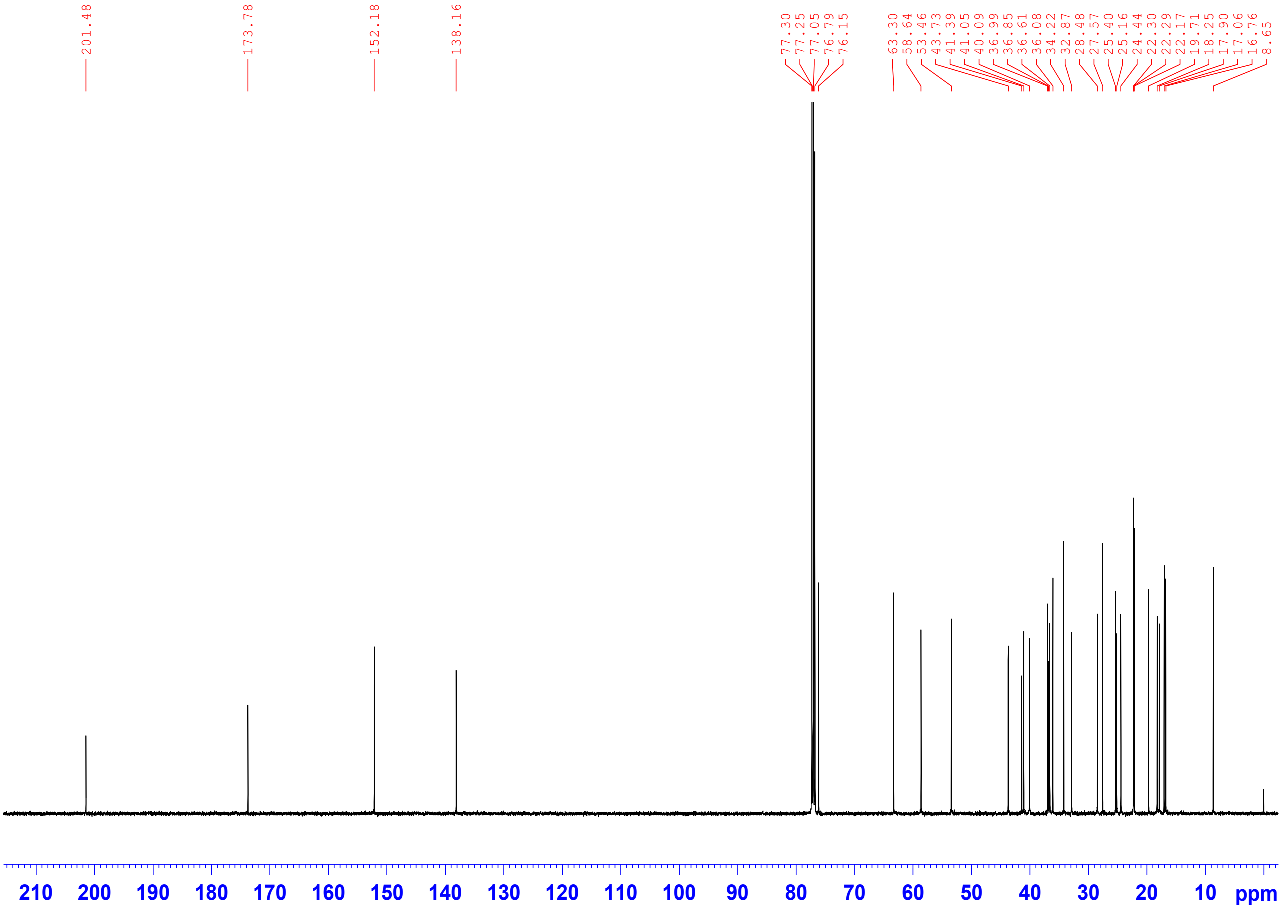

S47. DEPT135 spectrum of phyllofenone J (**5**) in $CDCl_3$.

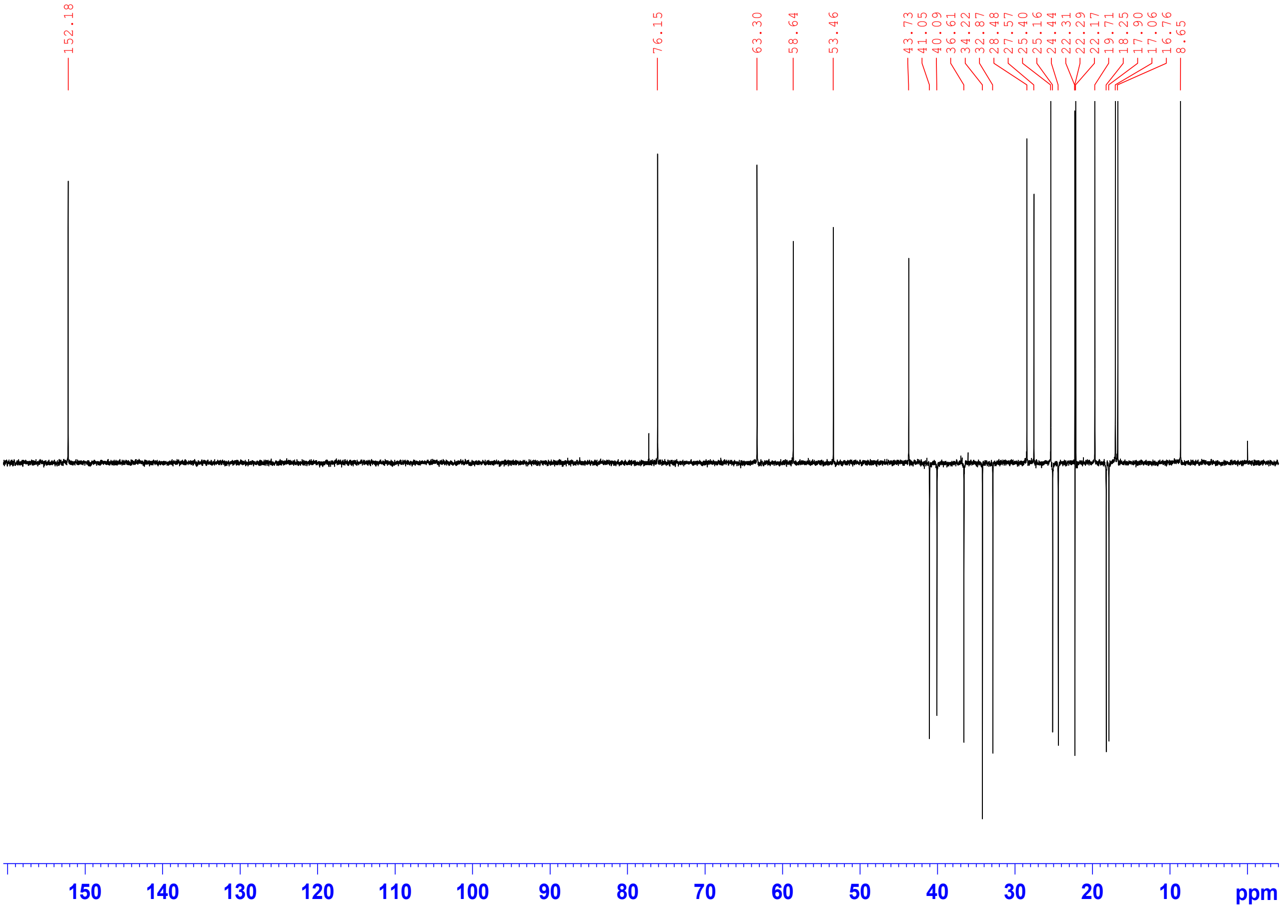

S48. HSQC spectrum of phyllofenone J (**5**) in $CDCl_3$.

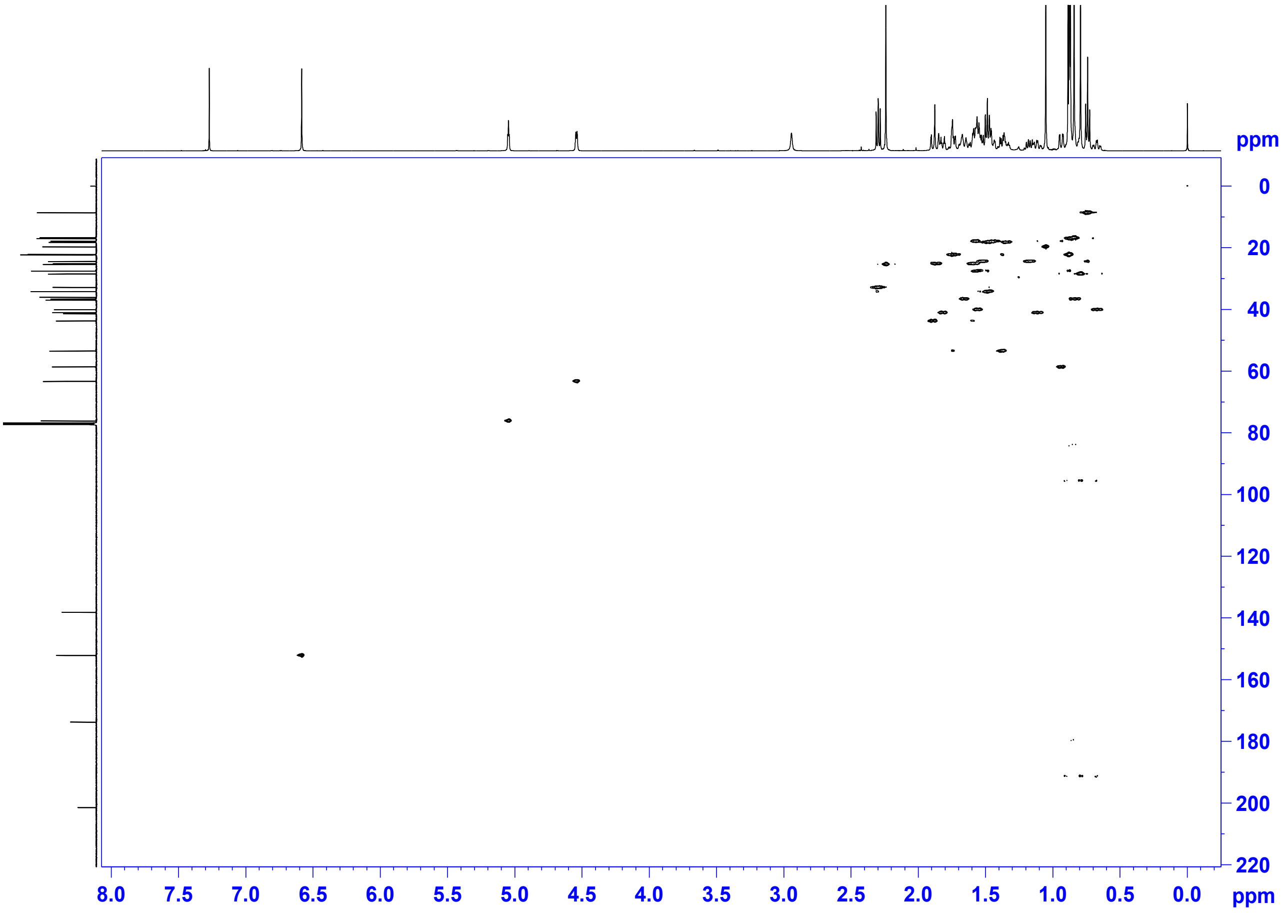

S49. COSY spectrum of phyllofenone J (**5**) in CDCl3.

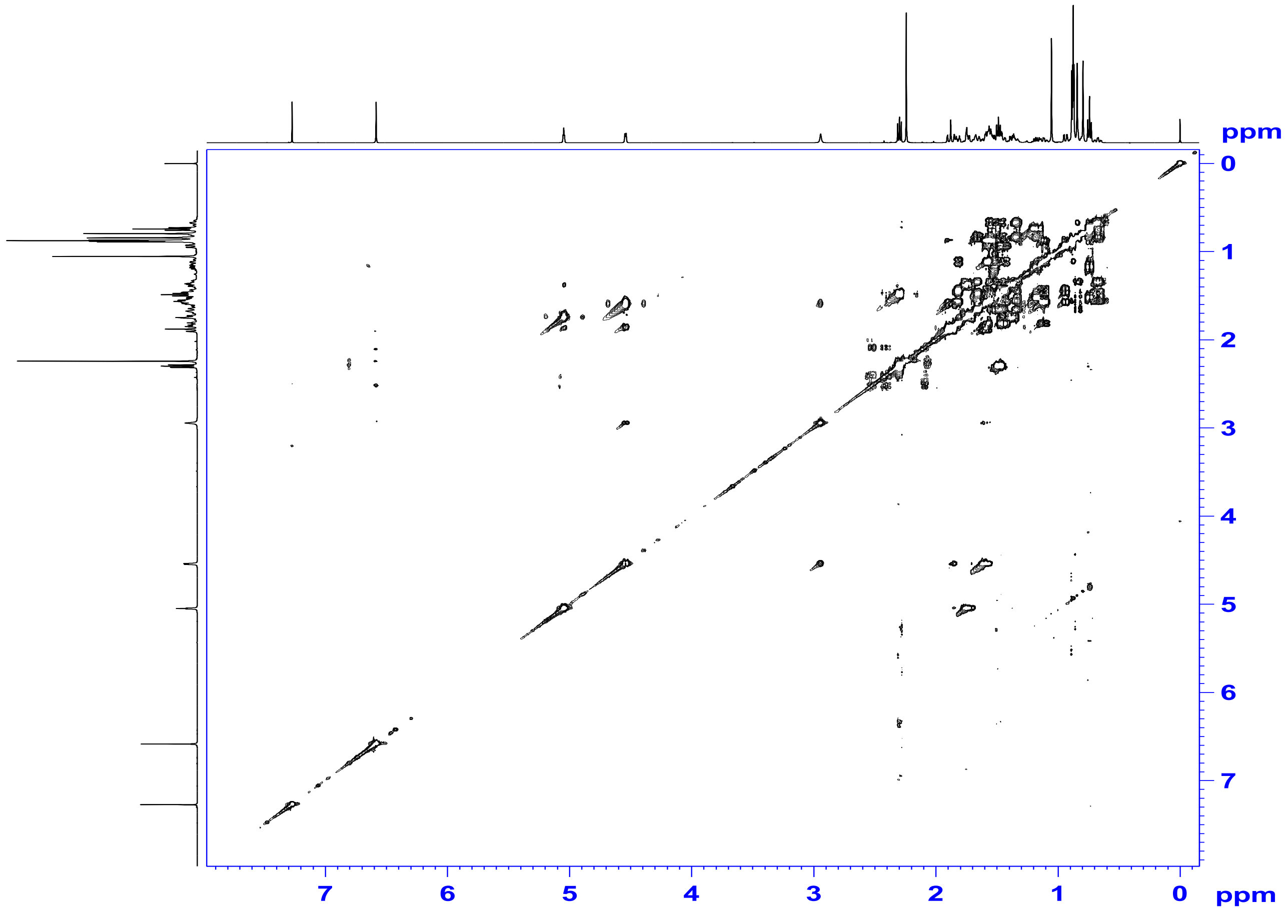

S50. HMBC spectrum of phyllofenone J (**5**) in CDCl3.

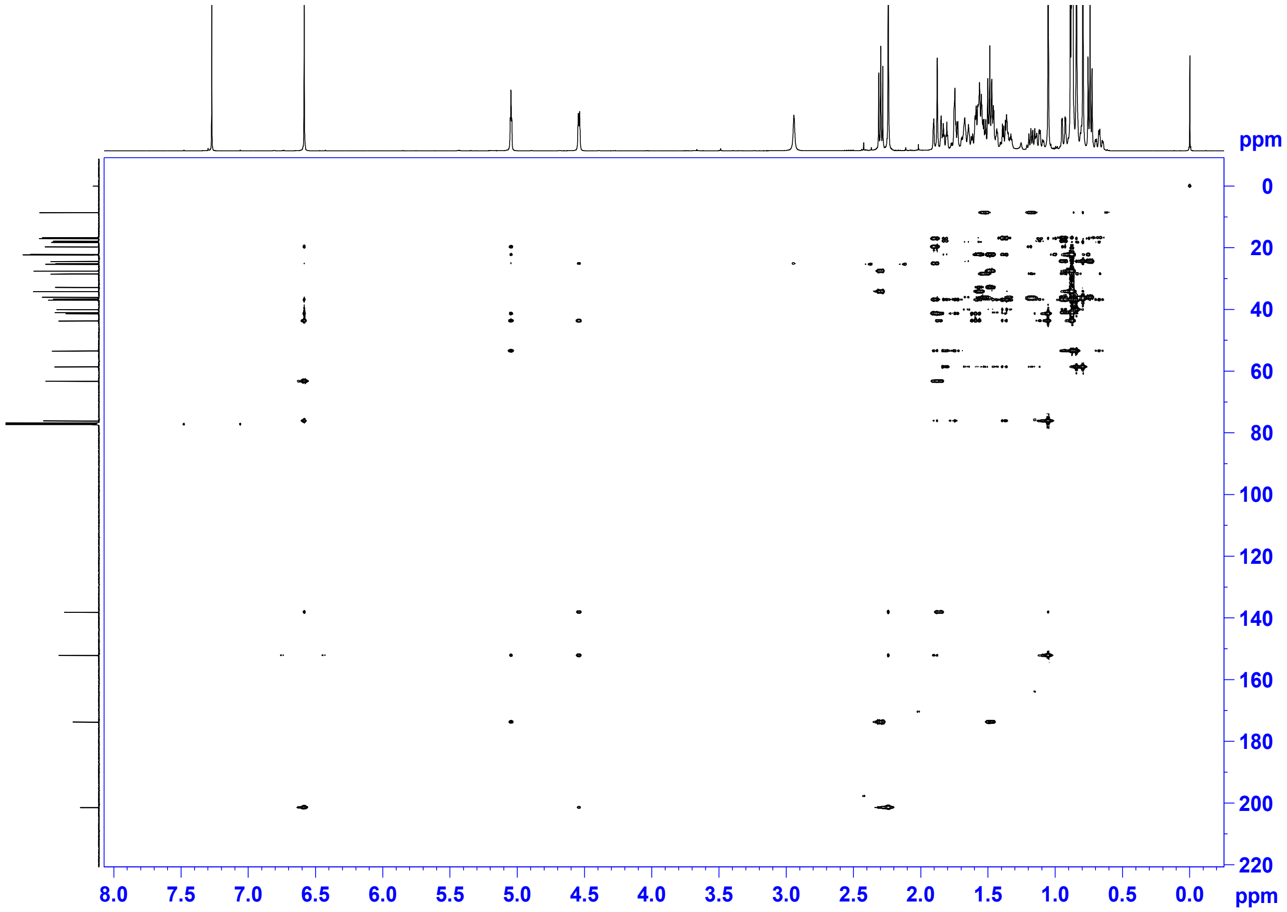

S51. NOESY spectrum of phyllofenone J (**5**) in $CDCl_3$.

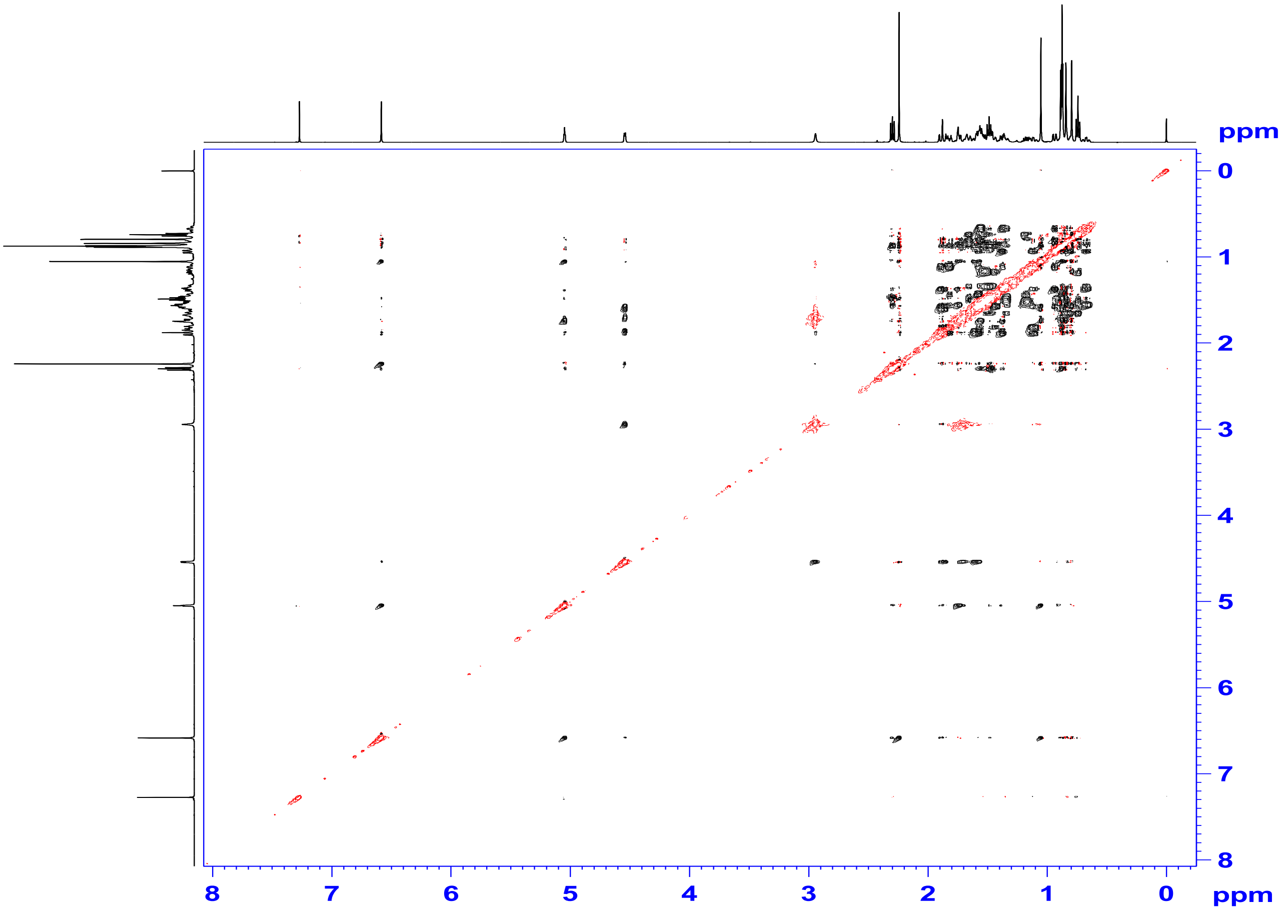

S54. HRESIMS of phyllofenone J (**5**).

Spectrum

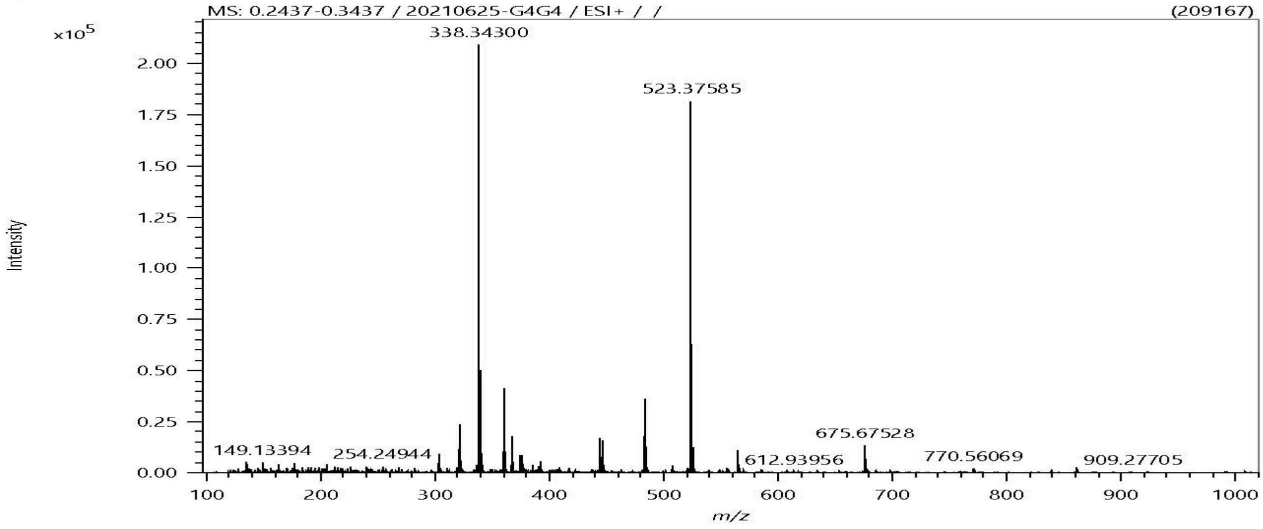

Elemental Composition

Parameters

Tolerance:	±5.00 ppm
Electron:	Odd/Even
Charge:	+1
DBE:	-1.5 - 200.0

Elements Set 1:

Symbol	C	H	N	O	Na	S	Cl	Br
Min	0	0	0	0	1	0	0	0
Max	200	200	0	8	1	0	0	0

Symbol	F	Si
Min	0	0
Max	0	0

Results

Mass	Intensity	Intensity [%]	Formula	Calculated Mass	Mass Difference [mDa]	Mass Difference [ppm]	DBE
523.37585	181504.85	86.78	C32 H52 O4 Na	523.37578	0.07	0.13	6.5

S53. UV spectrum of phyllofenone J (**5**).

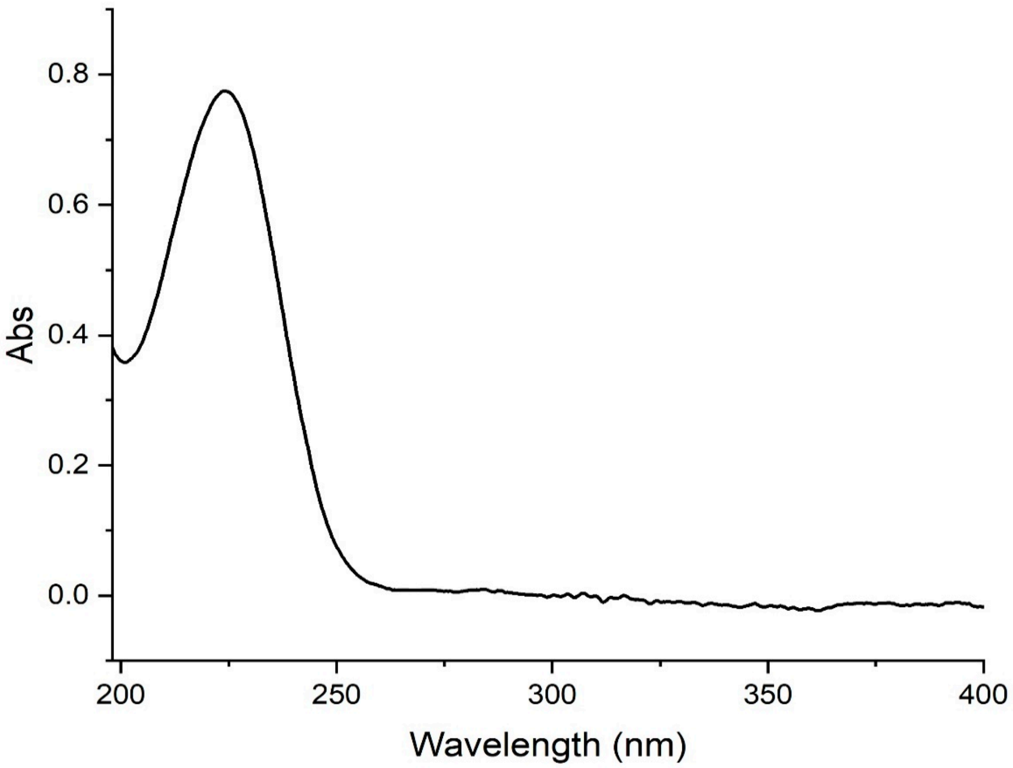

S54. IR spectrum of phyllofenone J (**5**) (KBr).

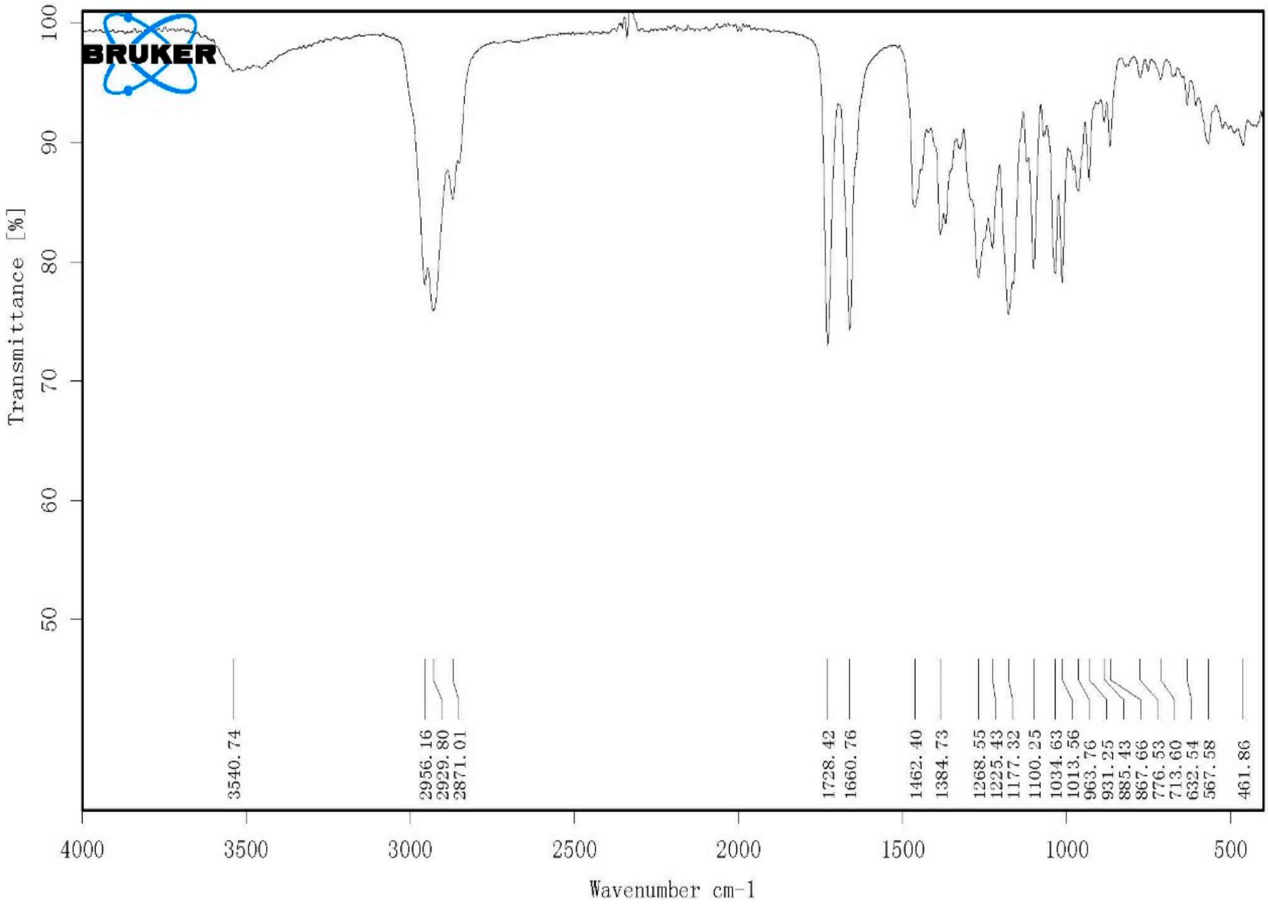

S55. ^{1}H NMR spectrum of phyllofenone K (**6**) in $CDCl_3$.

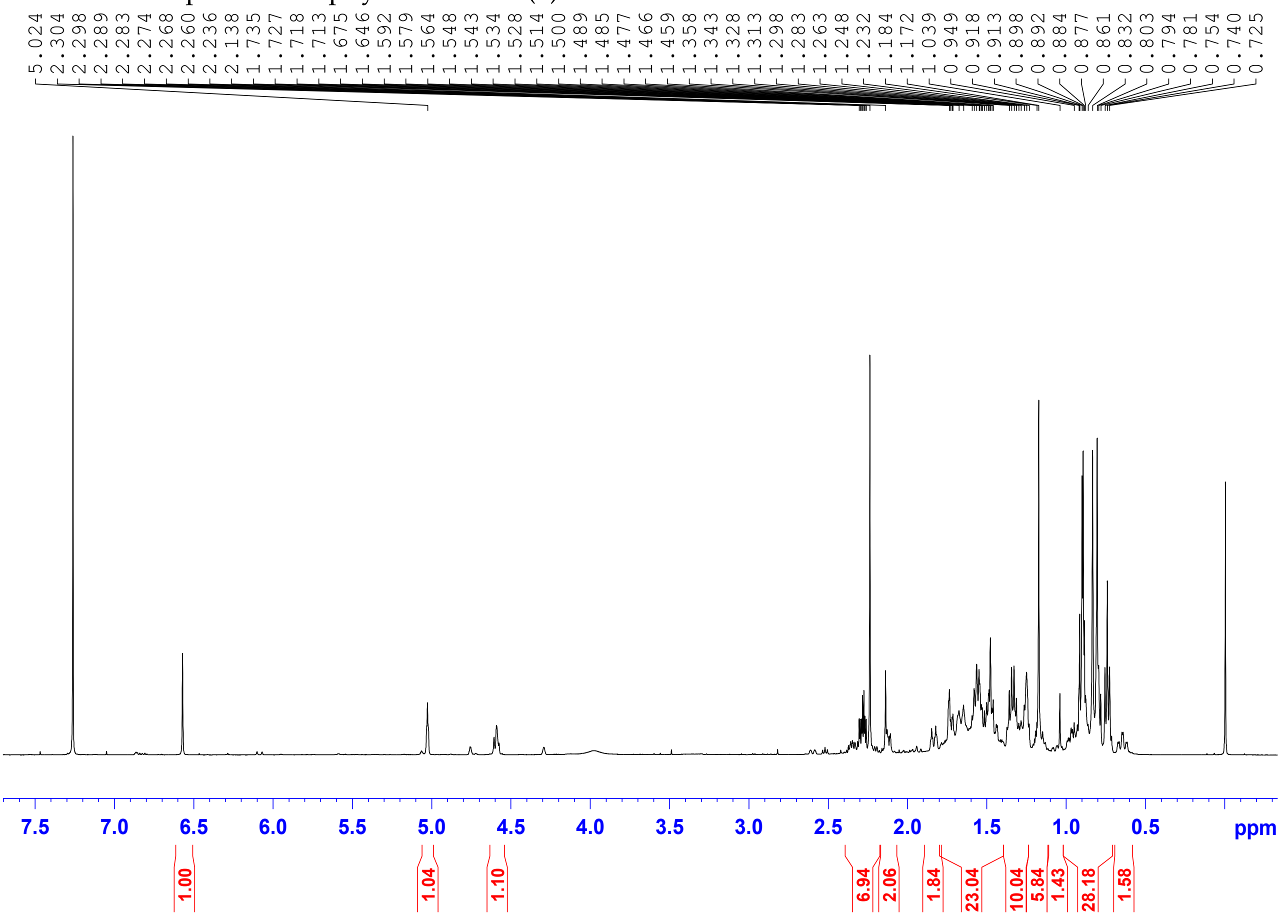

S56. ^{13}C NMR spectrum of phyllofenone K (**6**) in $CDCl_3$.

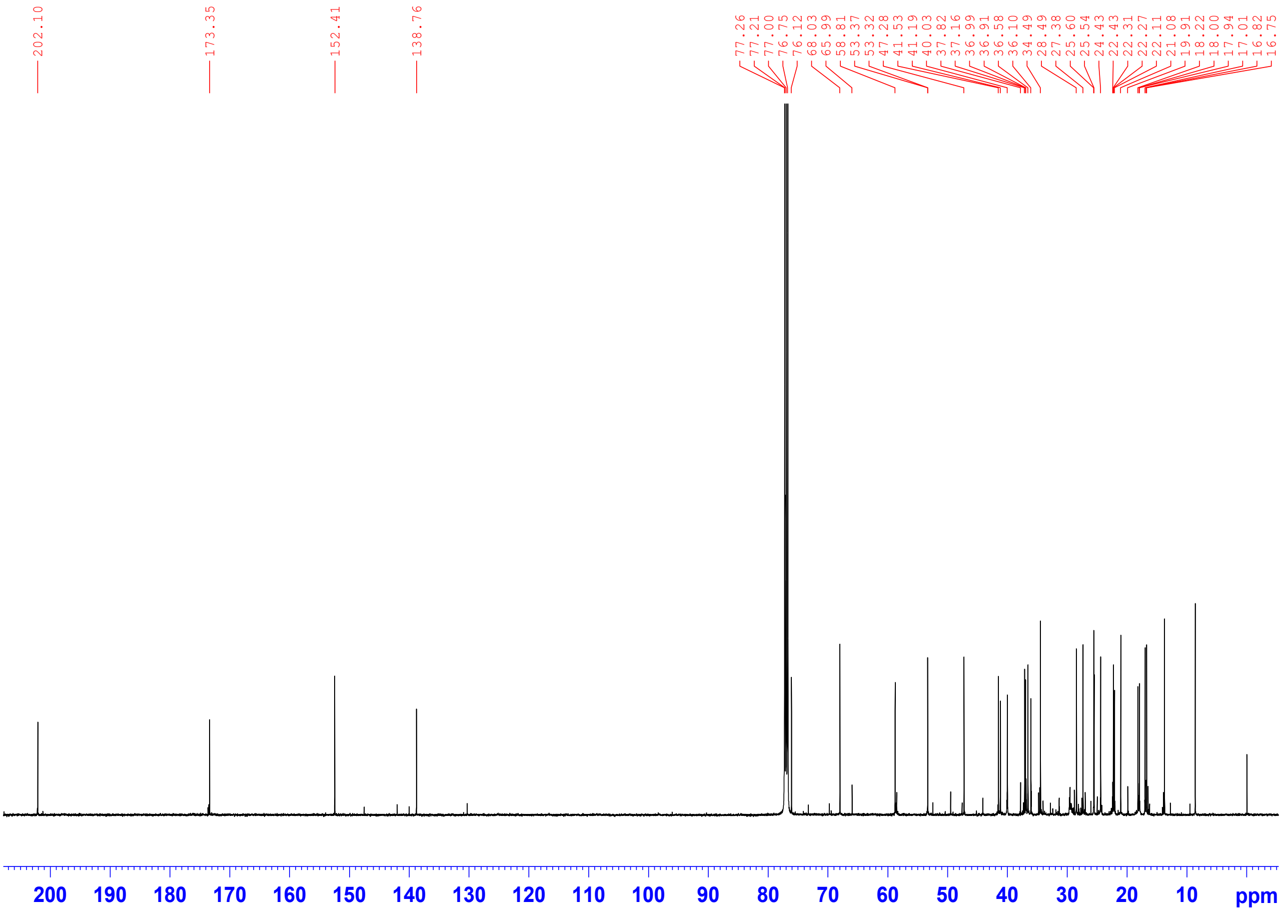

S57. DEPT135 spectrum of phyllofenone K (**6**) in $CDCl_3$.

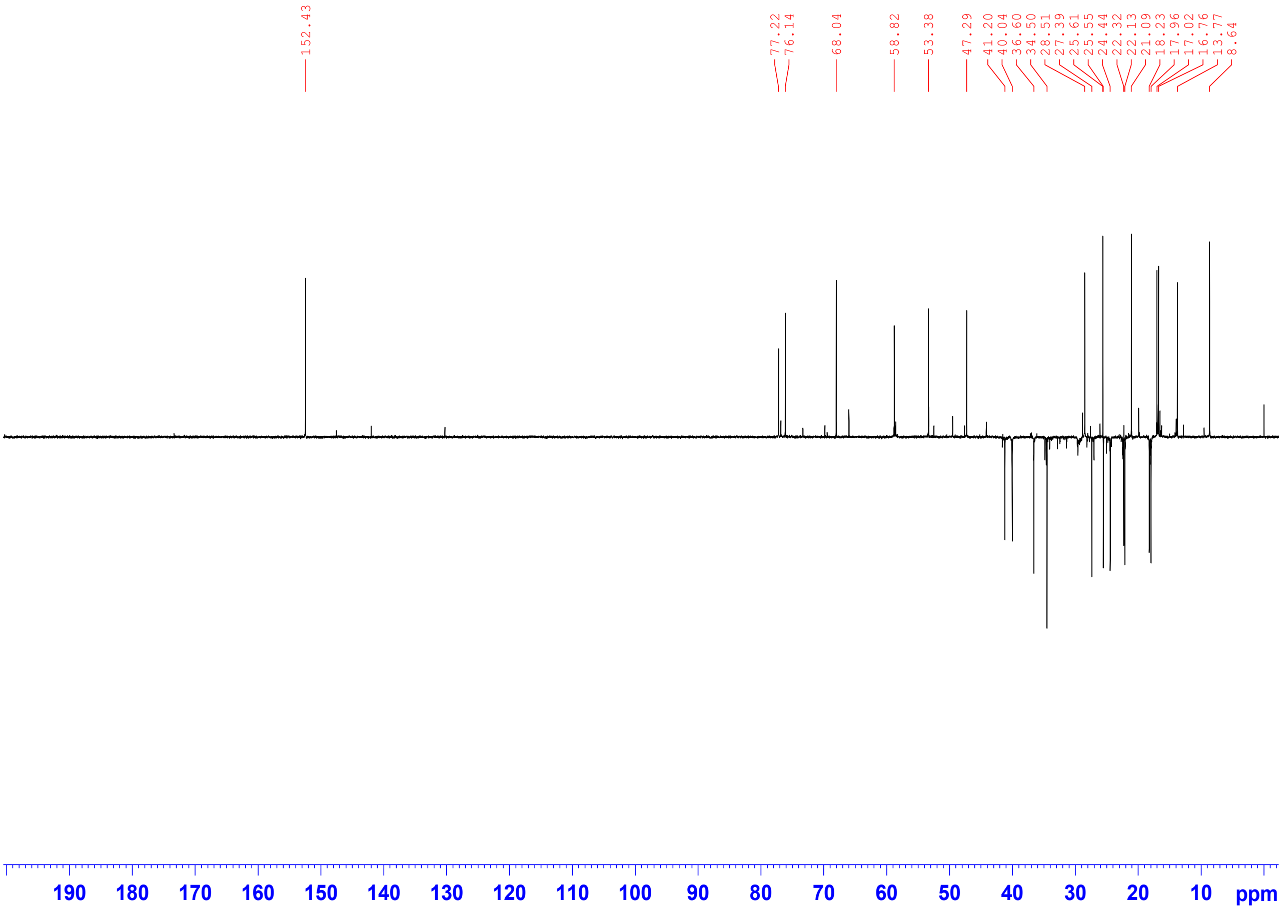

S58. HSQC spectrum of phyllofenone K (**6**) in $CDCl_3$.

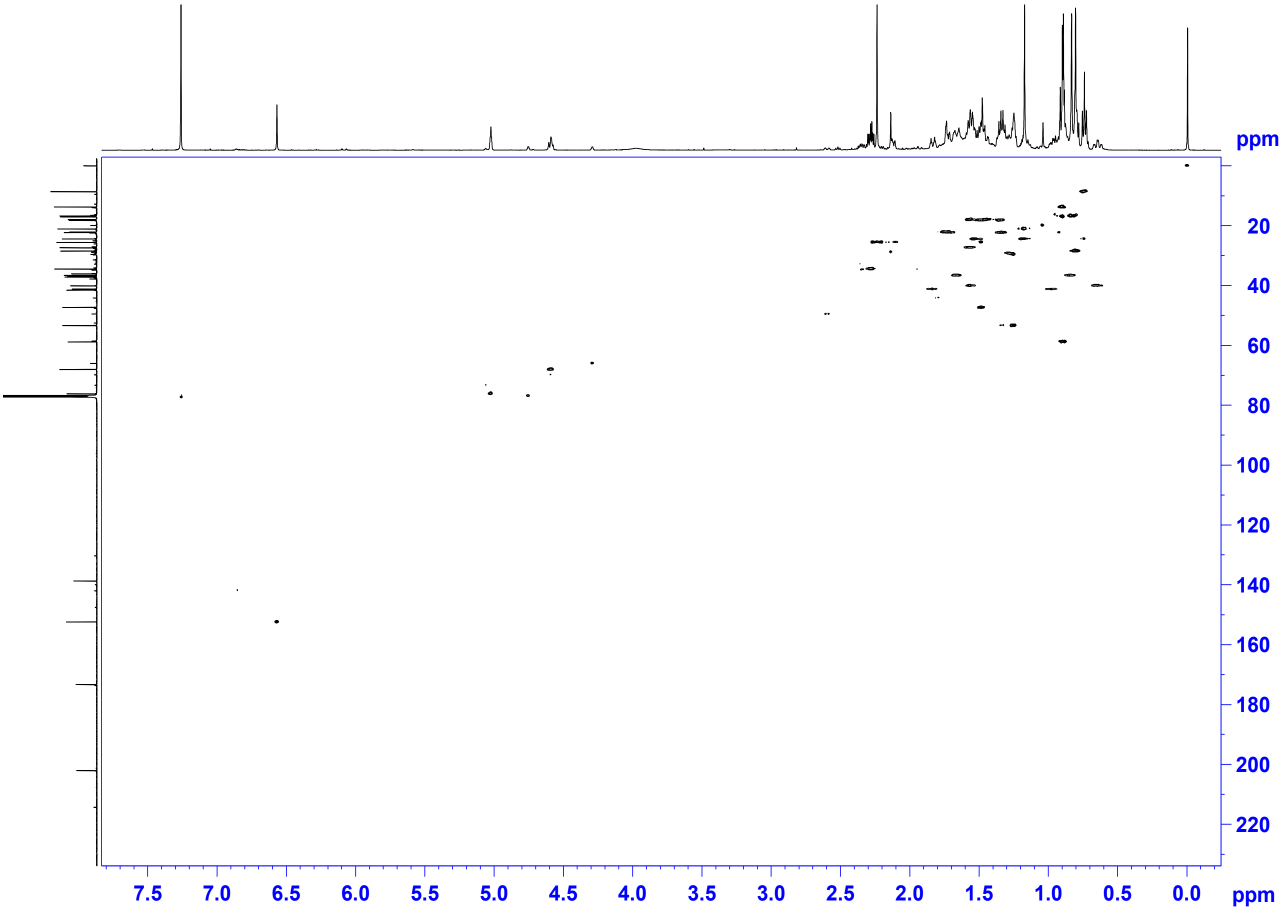

S59. COSY spectrum of phyllofenone K (**6**) in CDCl3.

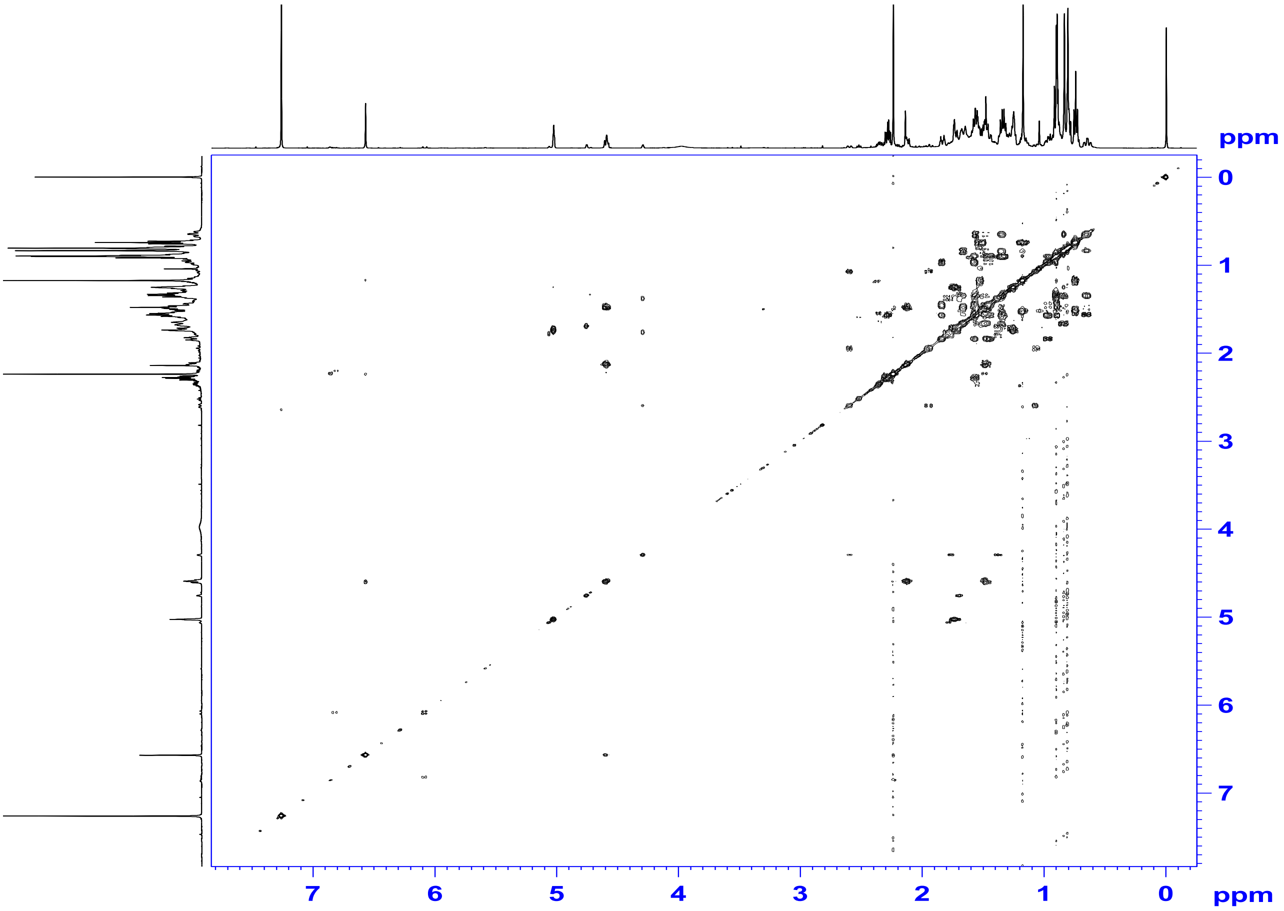

S60. HMBC spectrum of phyllofenone K (**6**) in CDCl3.

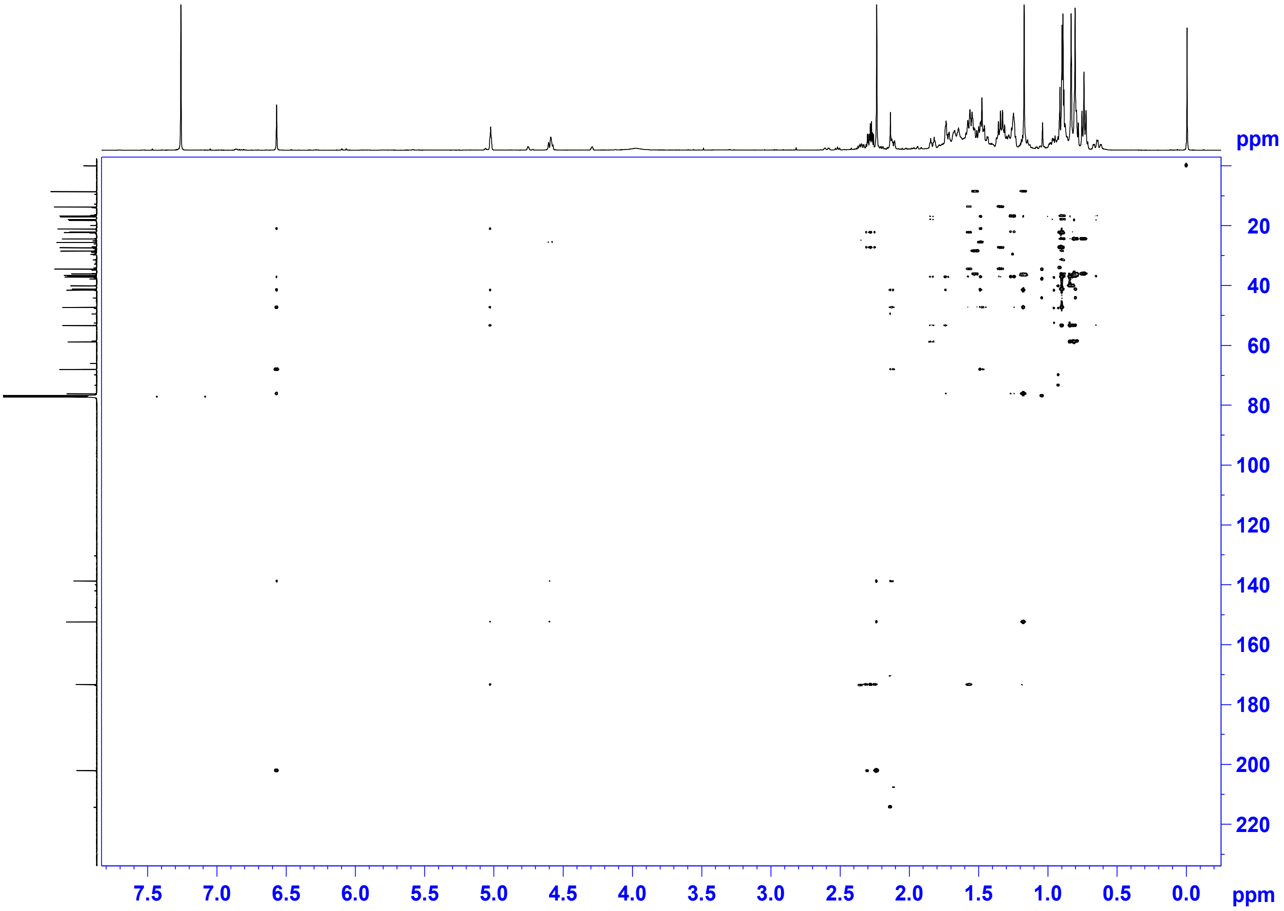

S61. NOESY spectrum of phyllofenone K (**6**) in $CDCl_3$.

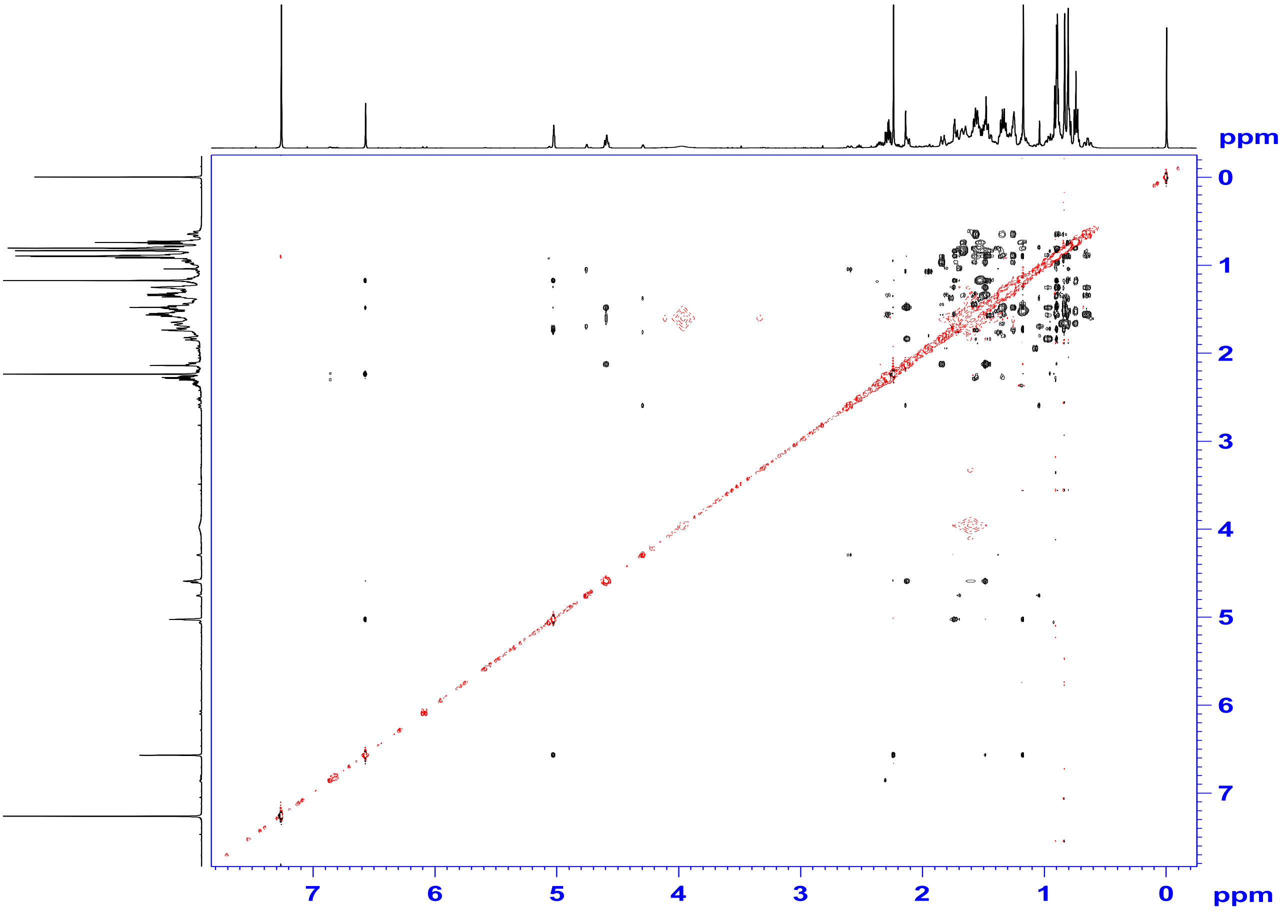

S62. HRESIMS of phyllofenone K (**6**).

Spectrum

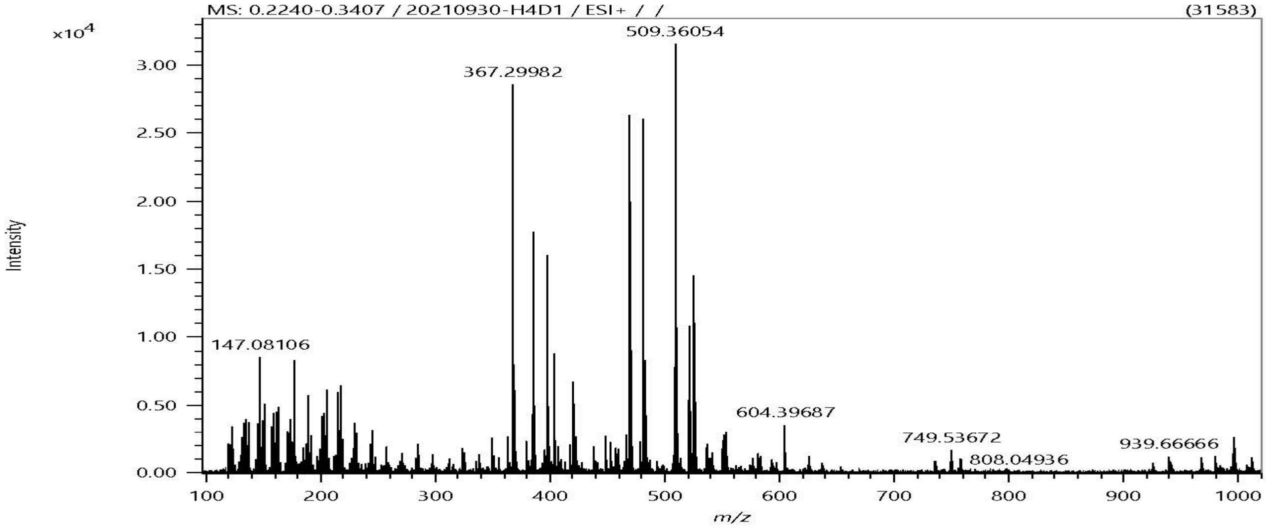

Elemental Composition

Parameters

Tolerance:	±5.00 ppm
Electron:	Odd/Even
Charge:	+1
DBE:	-1.5 - 200.0

Elements Set 1:

Symbol	C	H	N	O	Na	S	Cl	Br
Min	0	0	0	0	1	0	0	0
Max	200	200	0	8	1	0	0	0

Symbol	P	Si	F	I
Min	0	0	0	0
Max	0	0	0	0

Results

Mass	Intensity	Intensity [%]	Formula	Calculated Mass	Mass Difference [mDa]	Mass Difference [ppm]	DBE
509.36054	31582.55	100.00	C31 H50 O4 Na	509.36013	0.41	0.81	6.5

S63. UV spectrum of phyllofenone K (**6**).

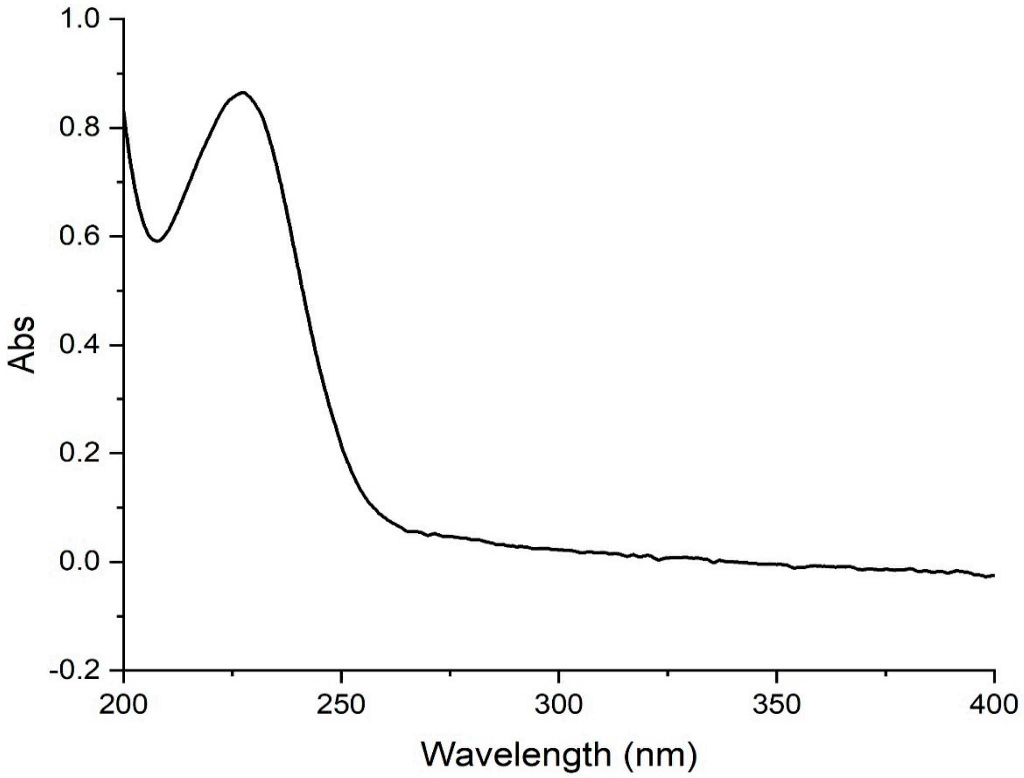

S64. IR spectrum of phyllofenone K (**6**) (KBr).

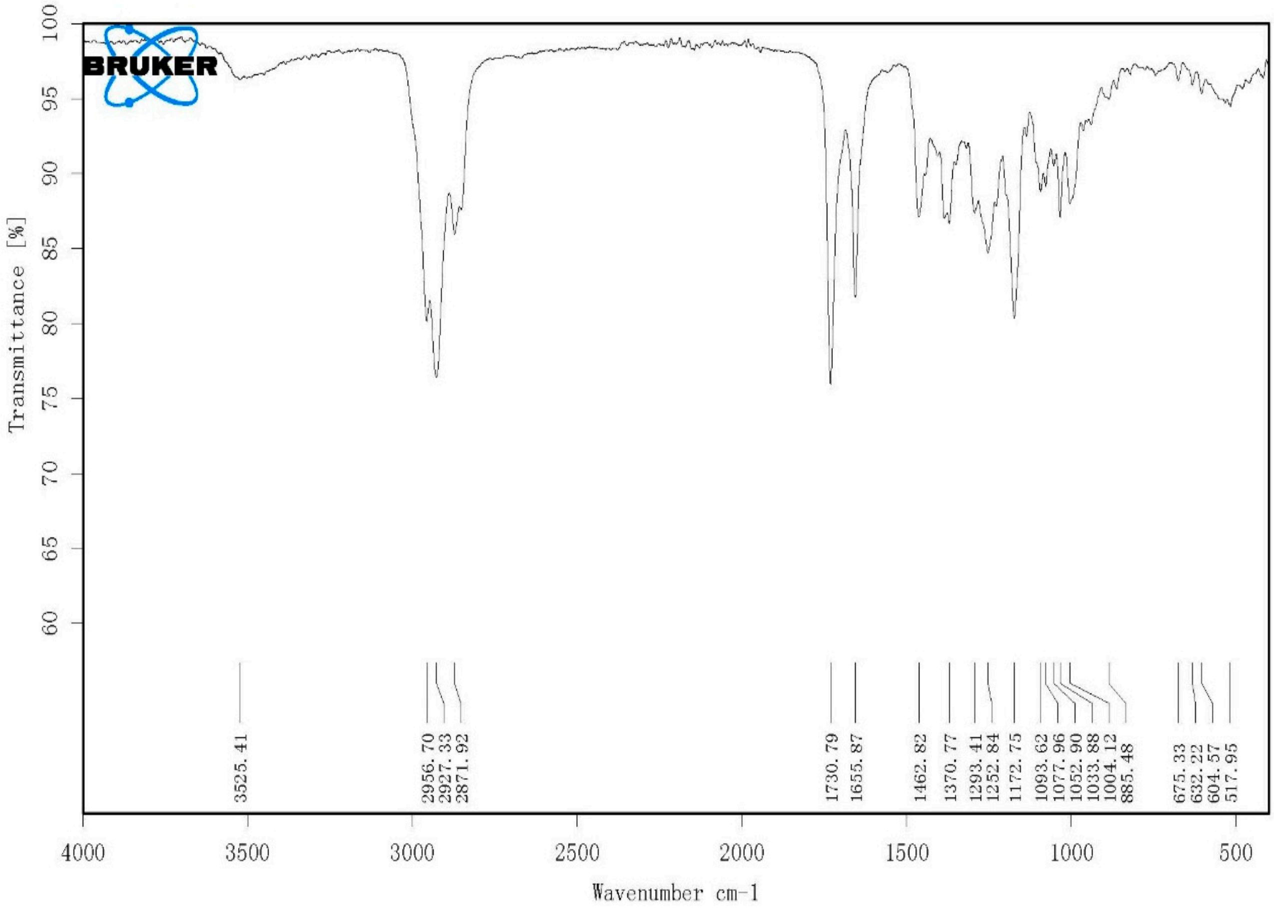

S65. ^{1}H NMR spectrum of phyllofenone L (**7**) in $CDCl_3$.

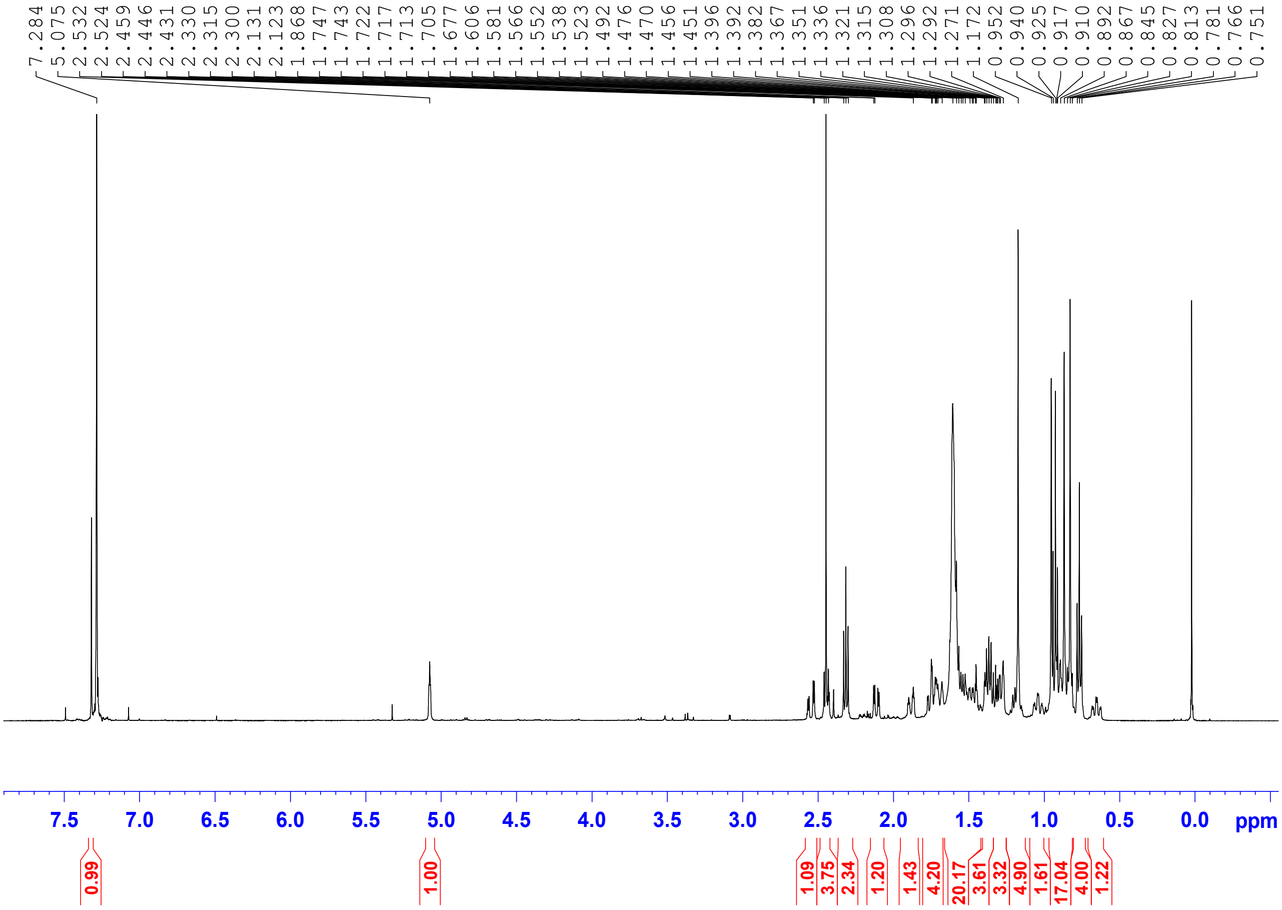

S66. ^{13}C NMR spectrum of phyllofenone L (**7**) in $CDCl_3$.

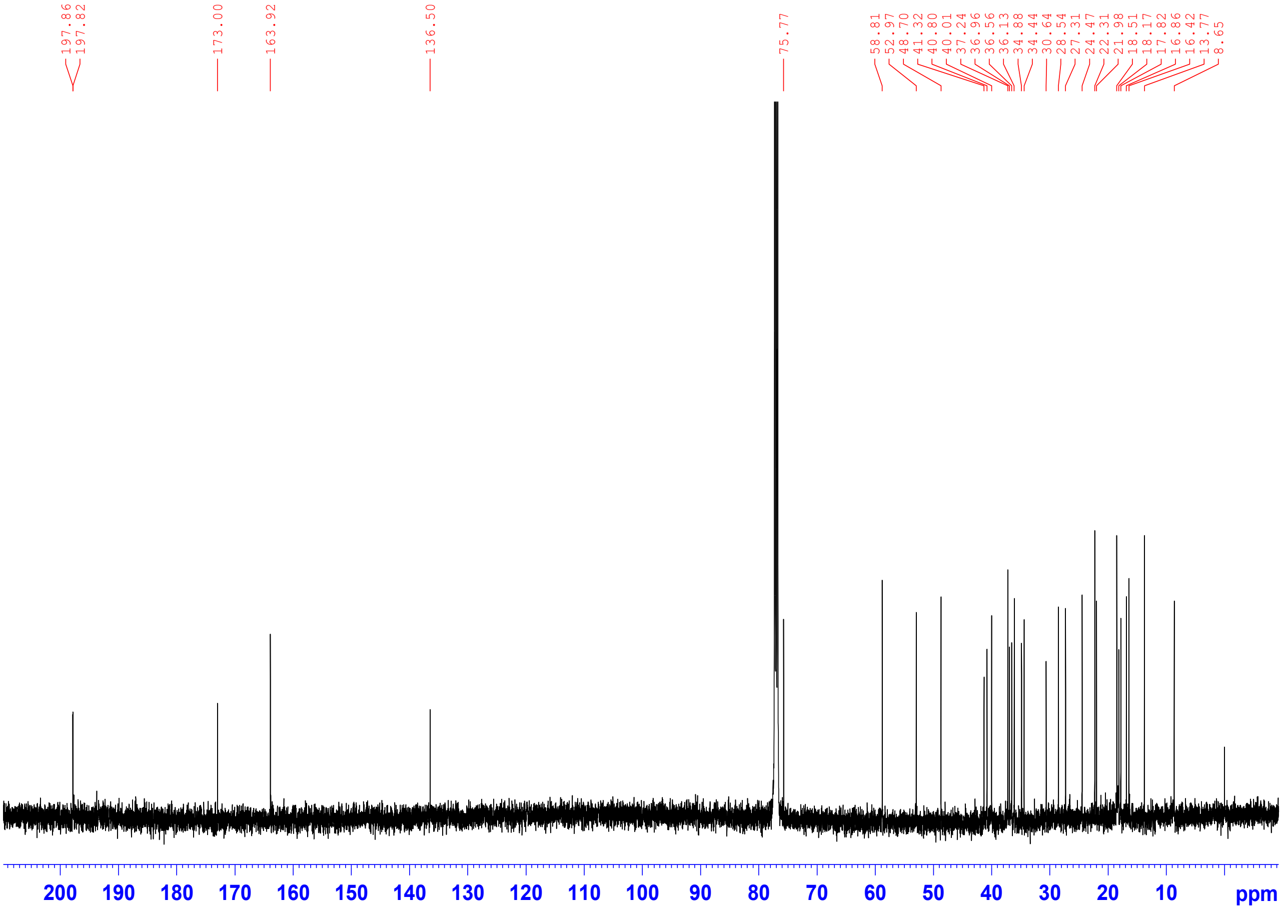

S67. DEPT135 spectrum of phyllofenone L (**7**) in $CDCl_3$.

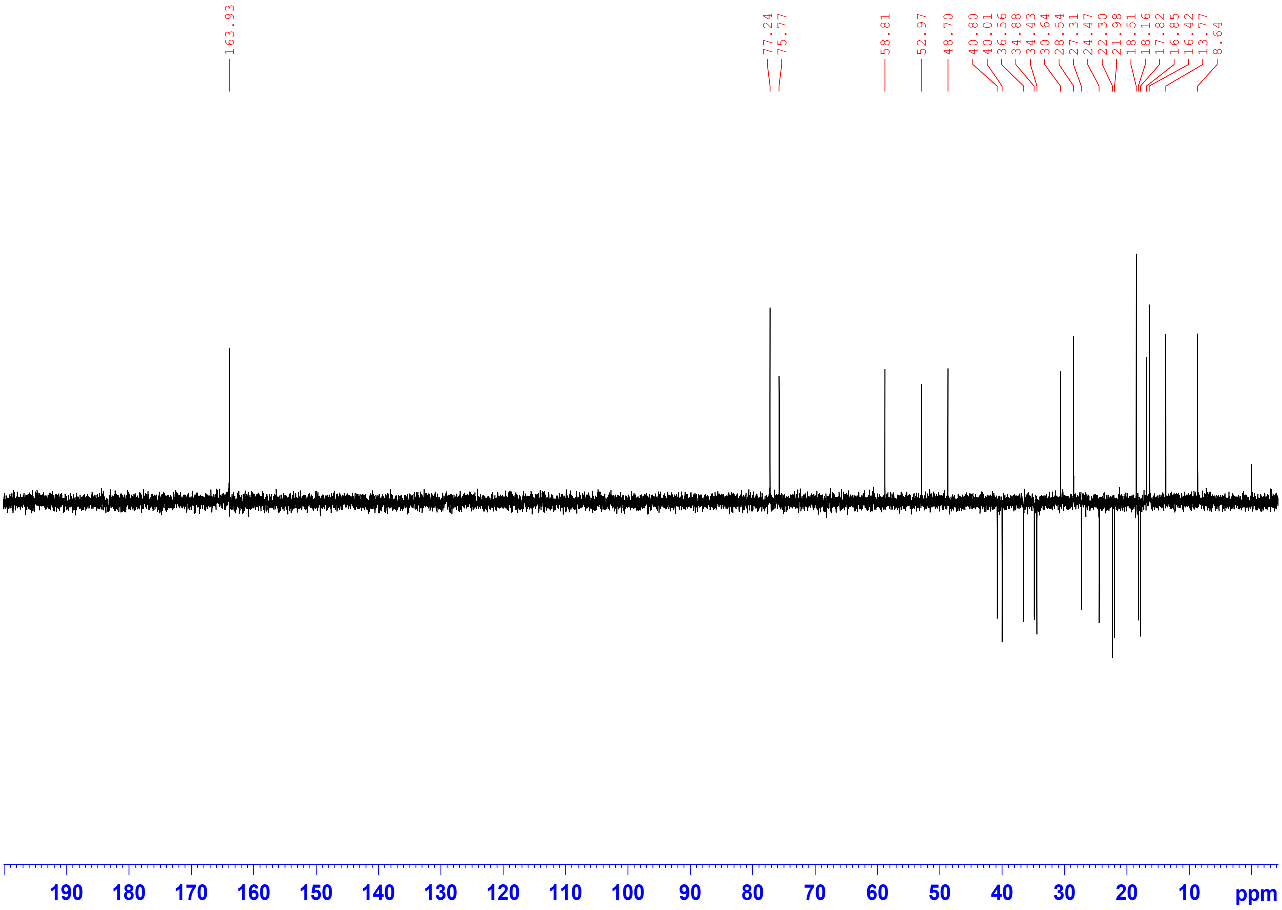

S68. HSQC spectrum of phyllofenone L (**7**) in $CDCl_3$.

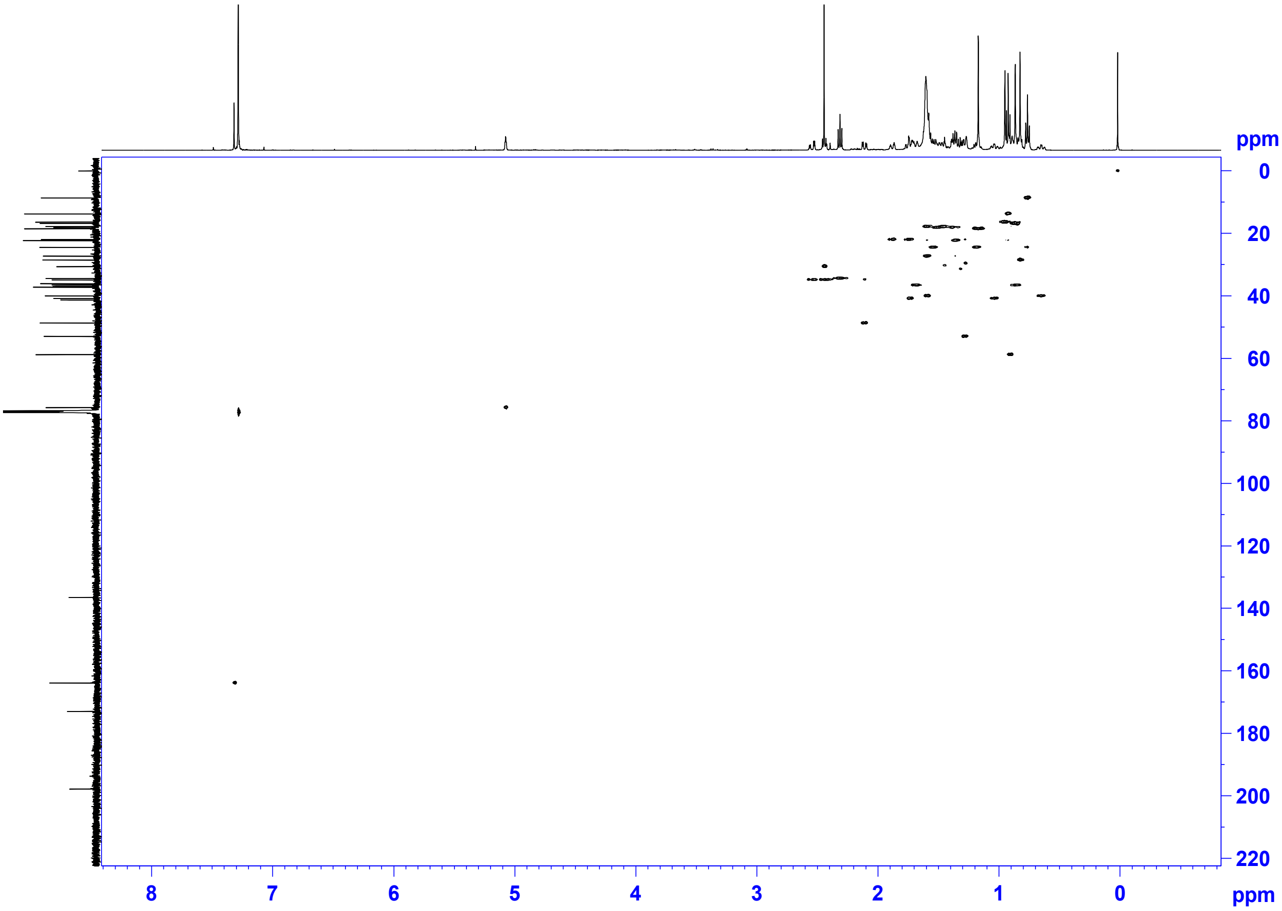

S69. COSY spectrum of phyllofenone L (7) in $CDCl_3$.

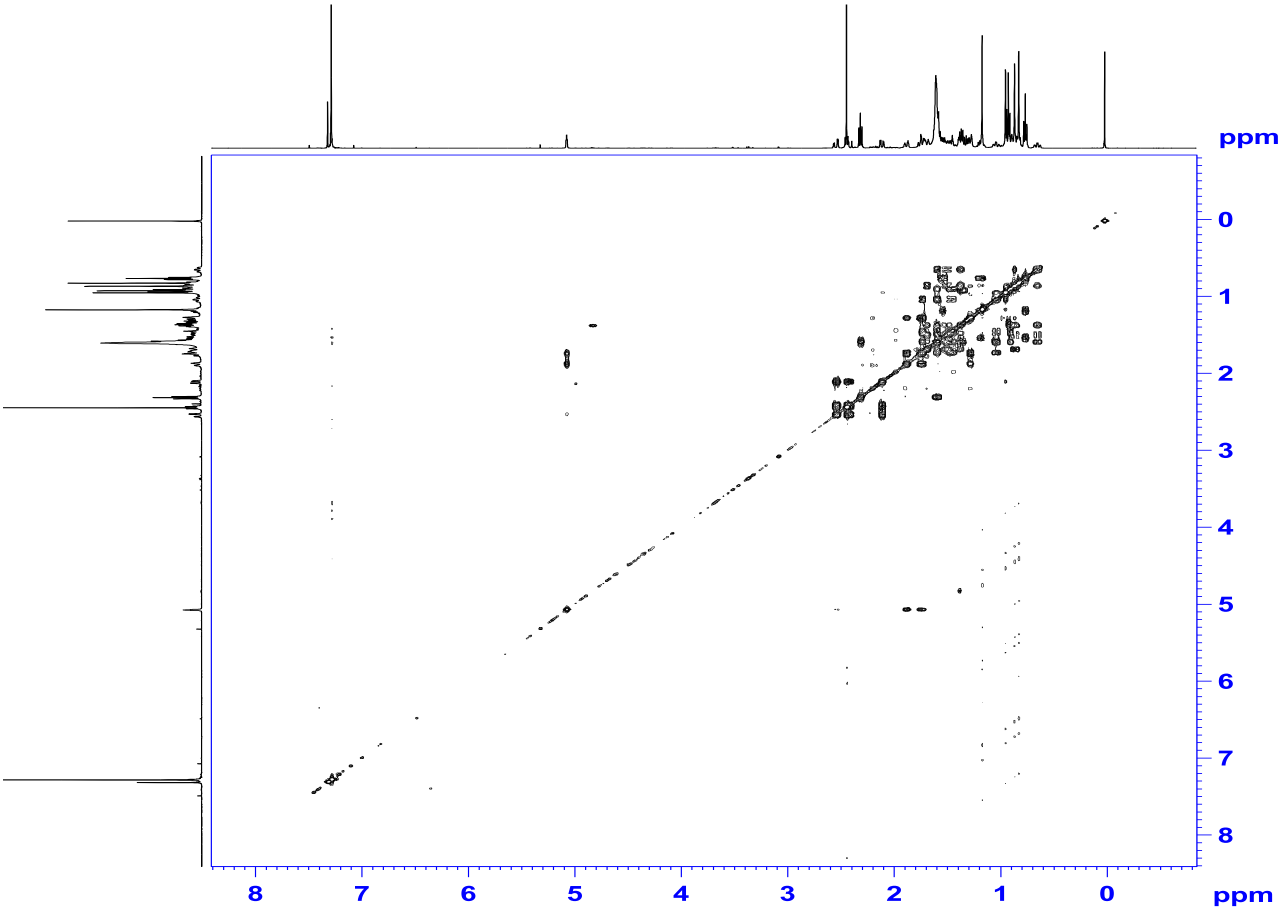

S70. HMBC spectrum of phyllofenone L (7) in $CDCl_3$.

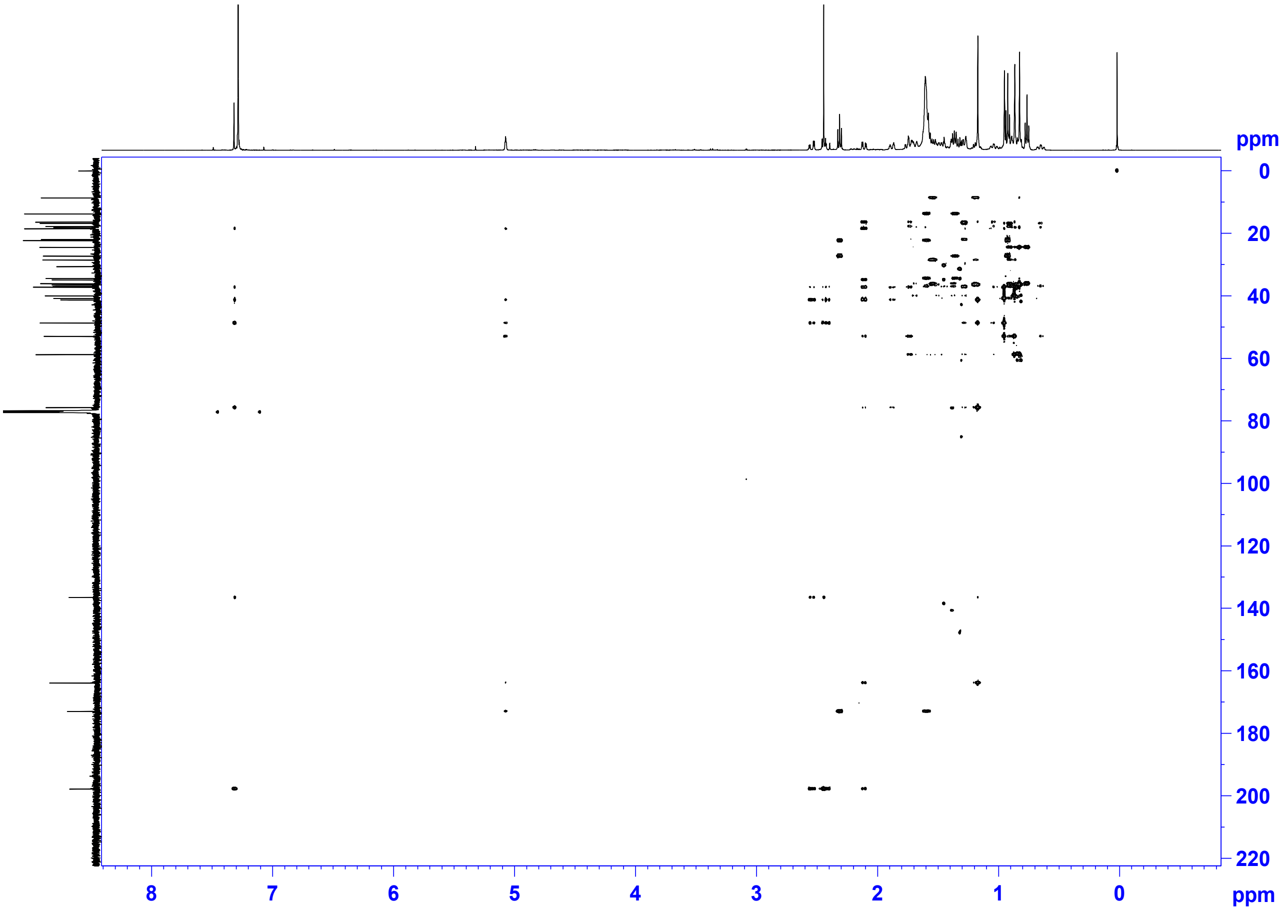

S71. NOESY spectrum of phyllofenone L (**7**) in $CDCl_3$.

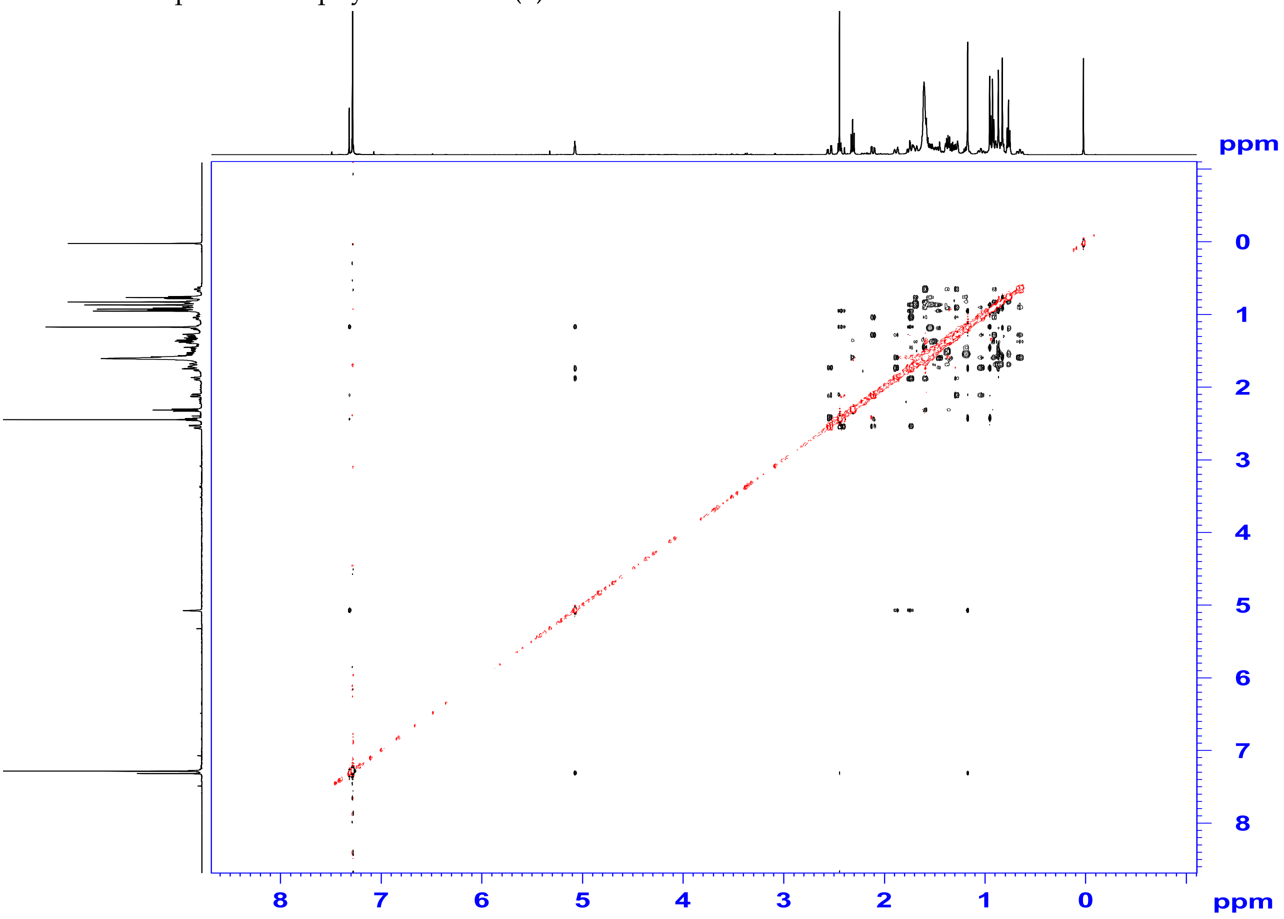

S72. HRESIMS of phyllofenone L (**7**).

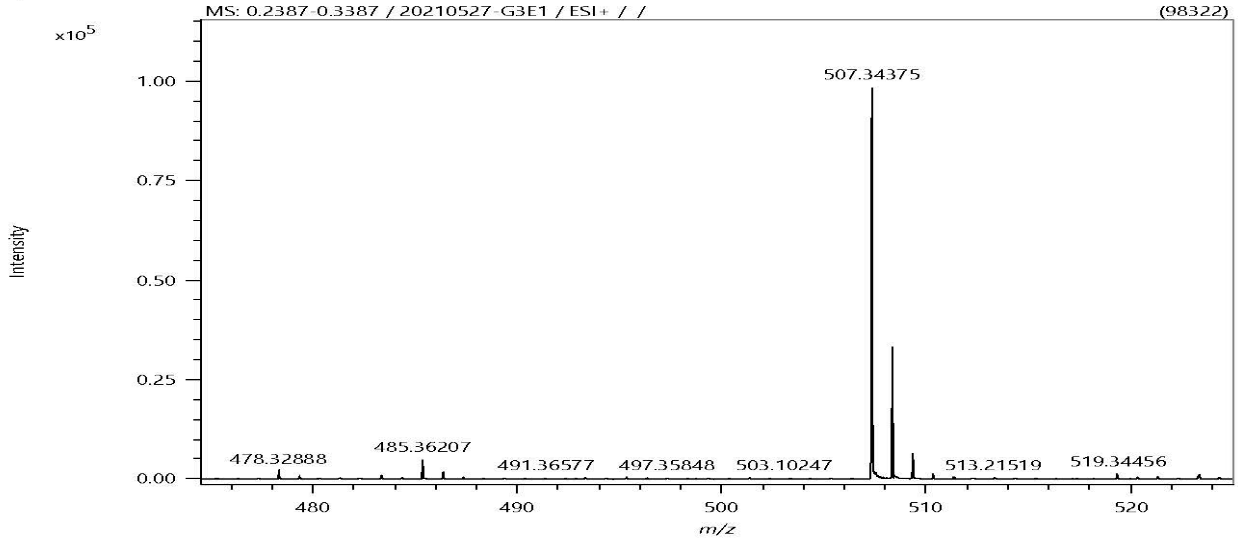

Elemental Composition

Parameters	
Tolerance:	±5.00 ppm
Electron:	Odd/Even
Charge:	+1
DBE:	-1.5 - 200.0

Elements Set 1:

Symbol	C	H	N	O	Na	S	Cl	Br
Min	0	0	0	0	1	0	0	0
Max	200	200	0	8	1	0	0	0

Symbol	F	Si
Min	0	0
Max	0	0

Results

Mass	Intensity	Intensity [%]	Formula	Calculated Mass	Mass Difference [mDa]	Mass Difference [ppm]	DBE
507.34375	98321.87	100.00	C31 H48 O4 Na	507.34448	-0.74	-1.45	7.5

S73. UV spectrum of phyllofenone L (**7**) in MeOH.

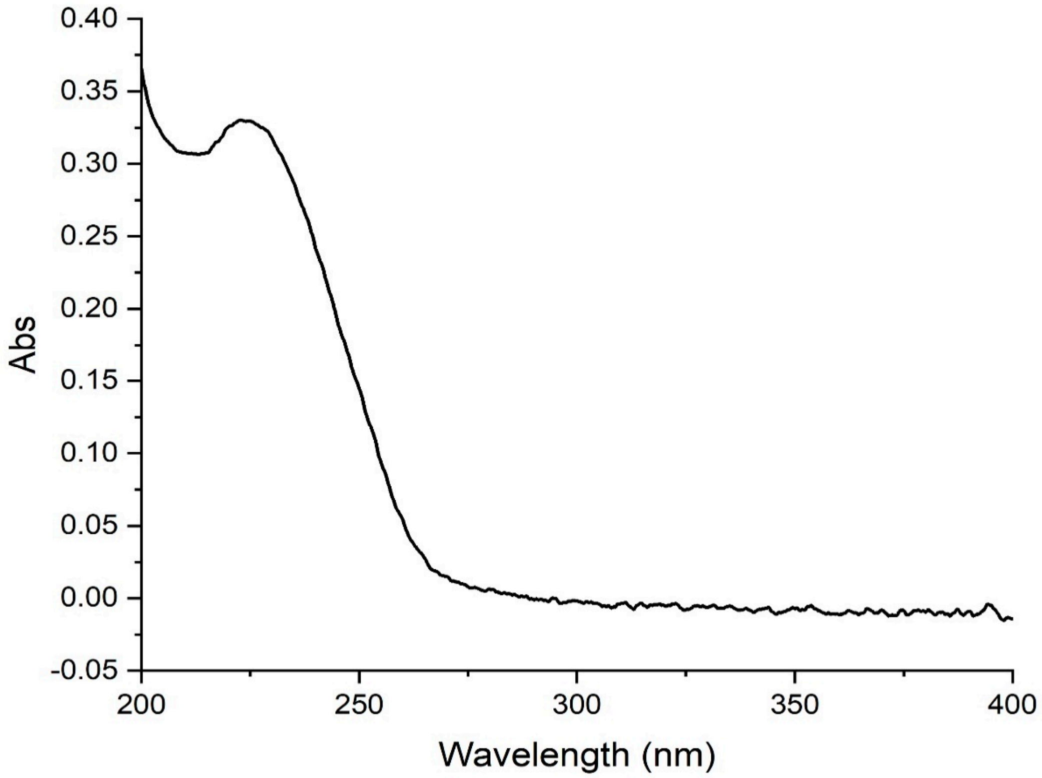

S74. IR spectrum of phyllofenone L (**7**) (KBr).

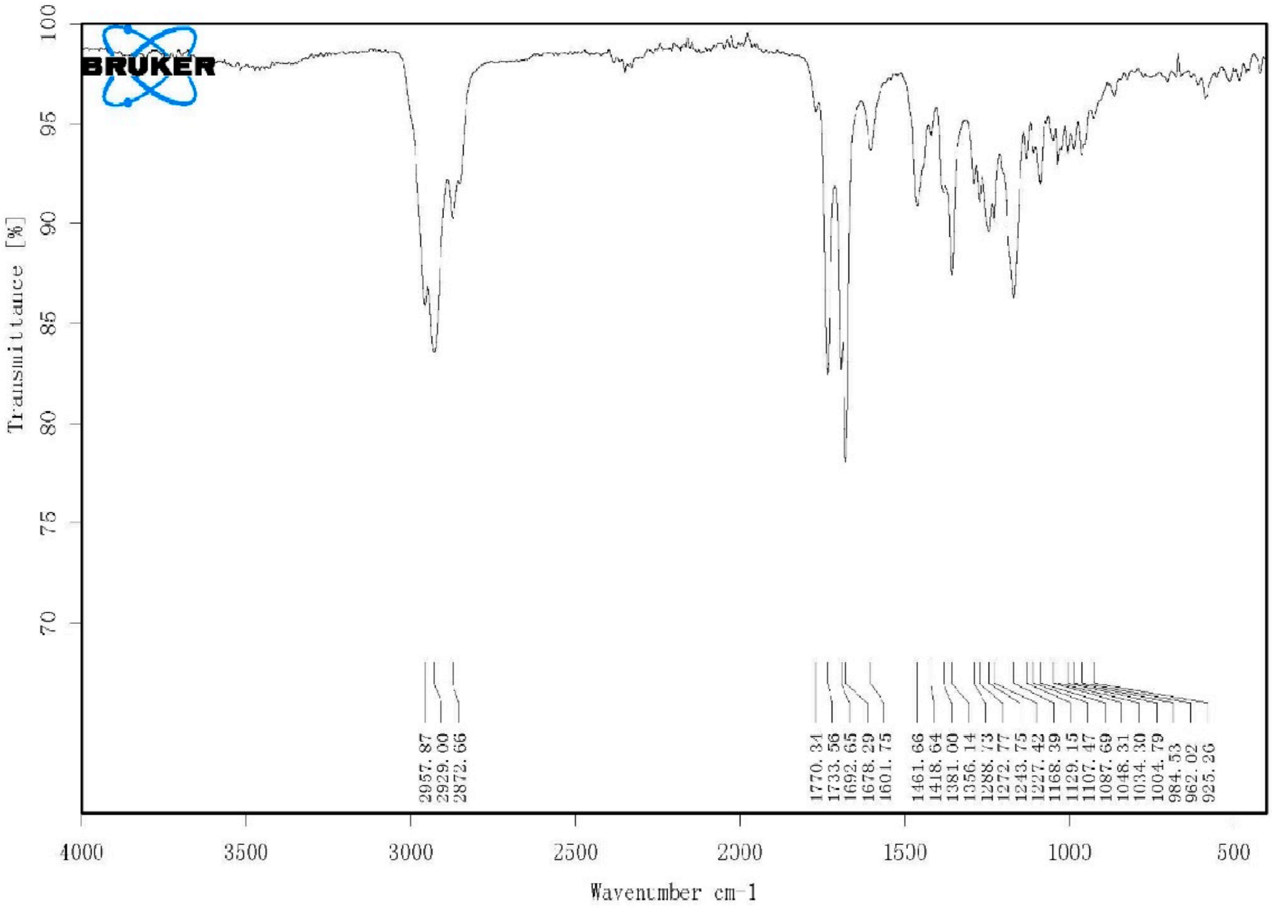

S75. ^{1}H NMR spectrum of phyllofenone M (**8**) in $CDCl_3$.

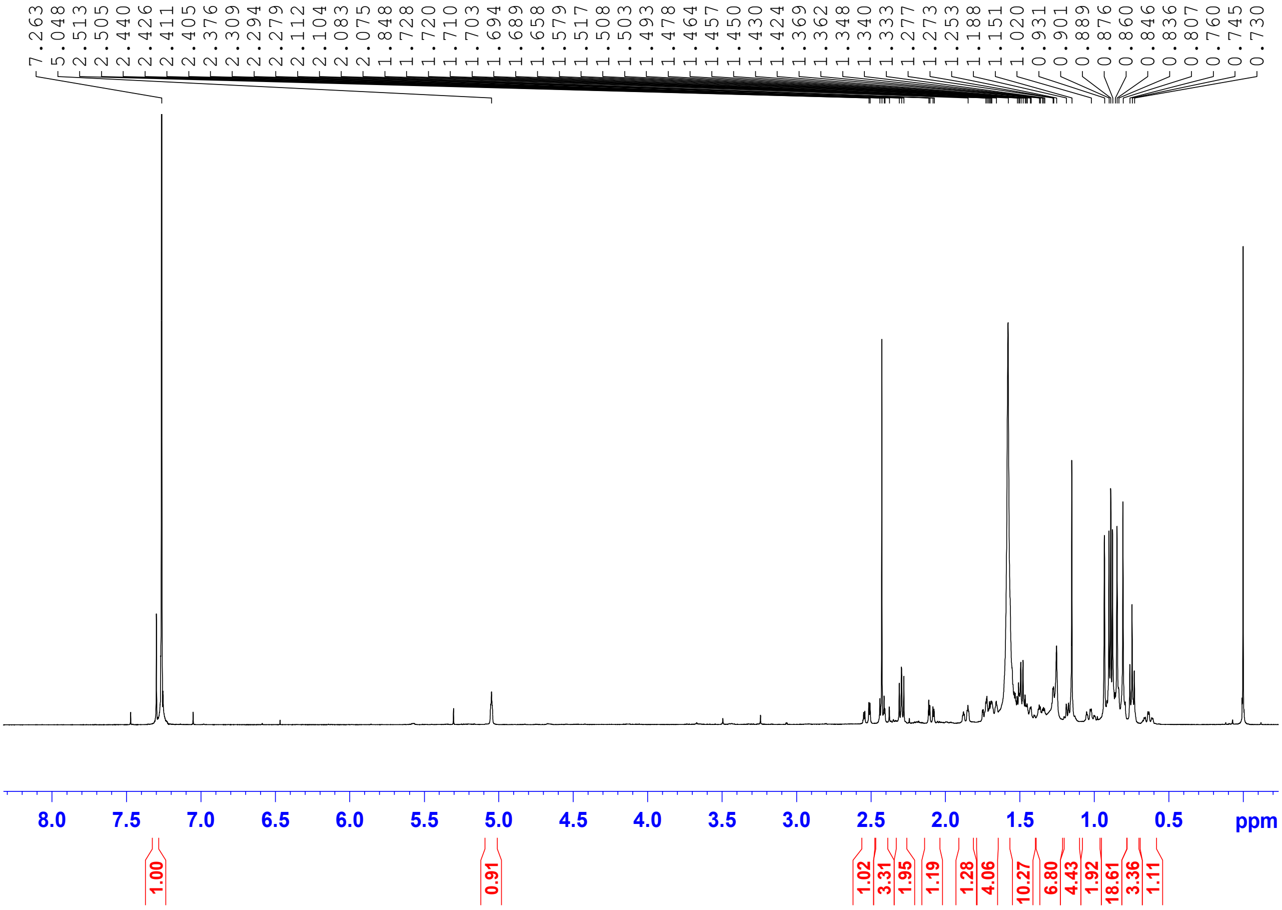

S76. ^{13}C NMR spectrum of phyllofenone M (**8**) in $CDCl_3$.

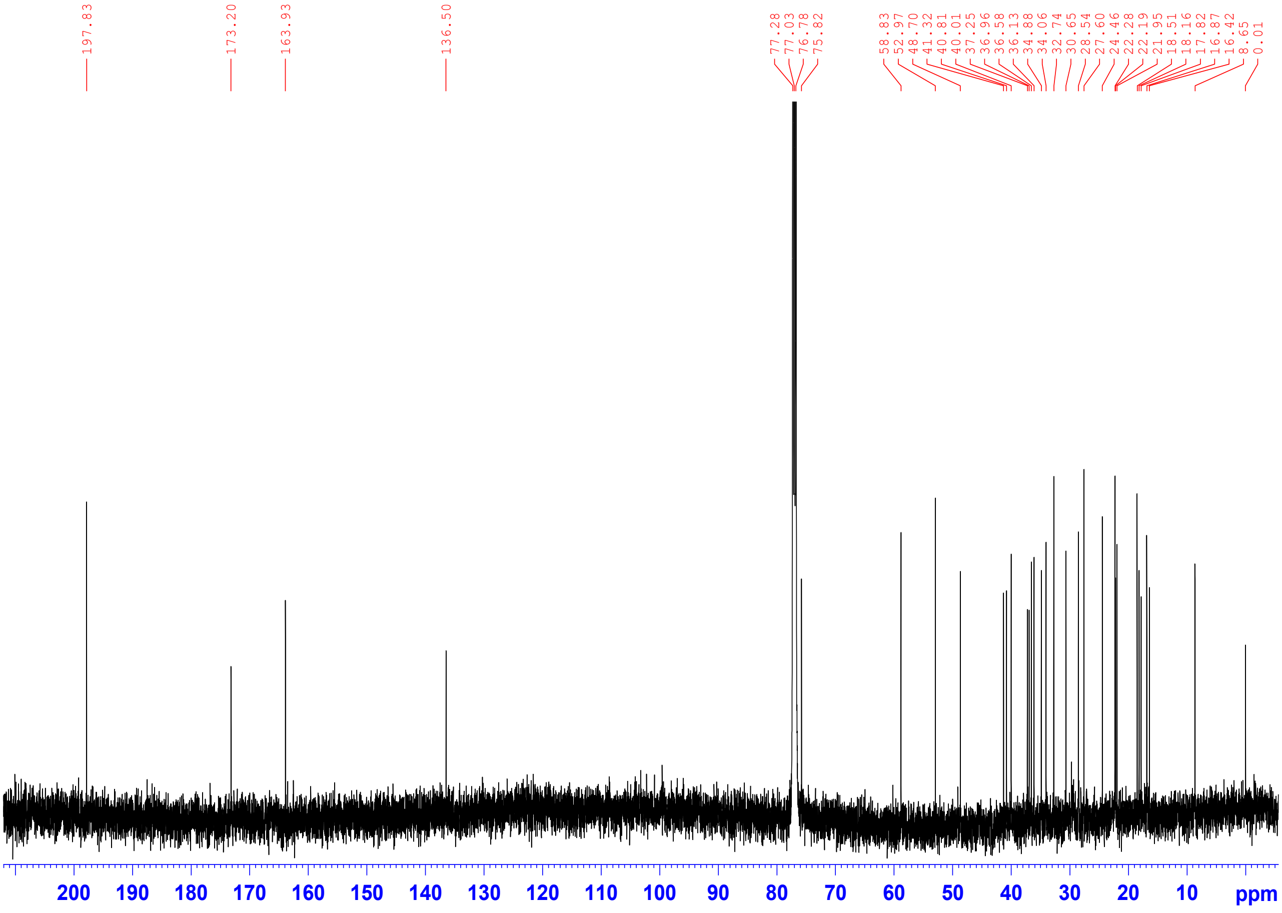

S77. DEPT135 spectrum of phyllofenone M (**8**) in $CDCl_3$.

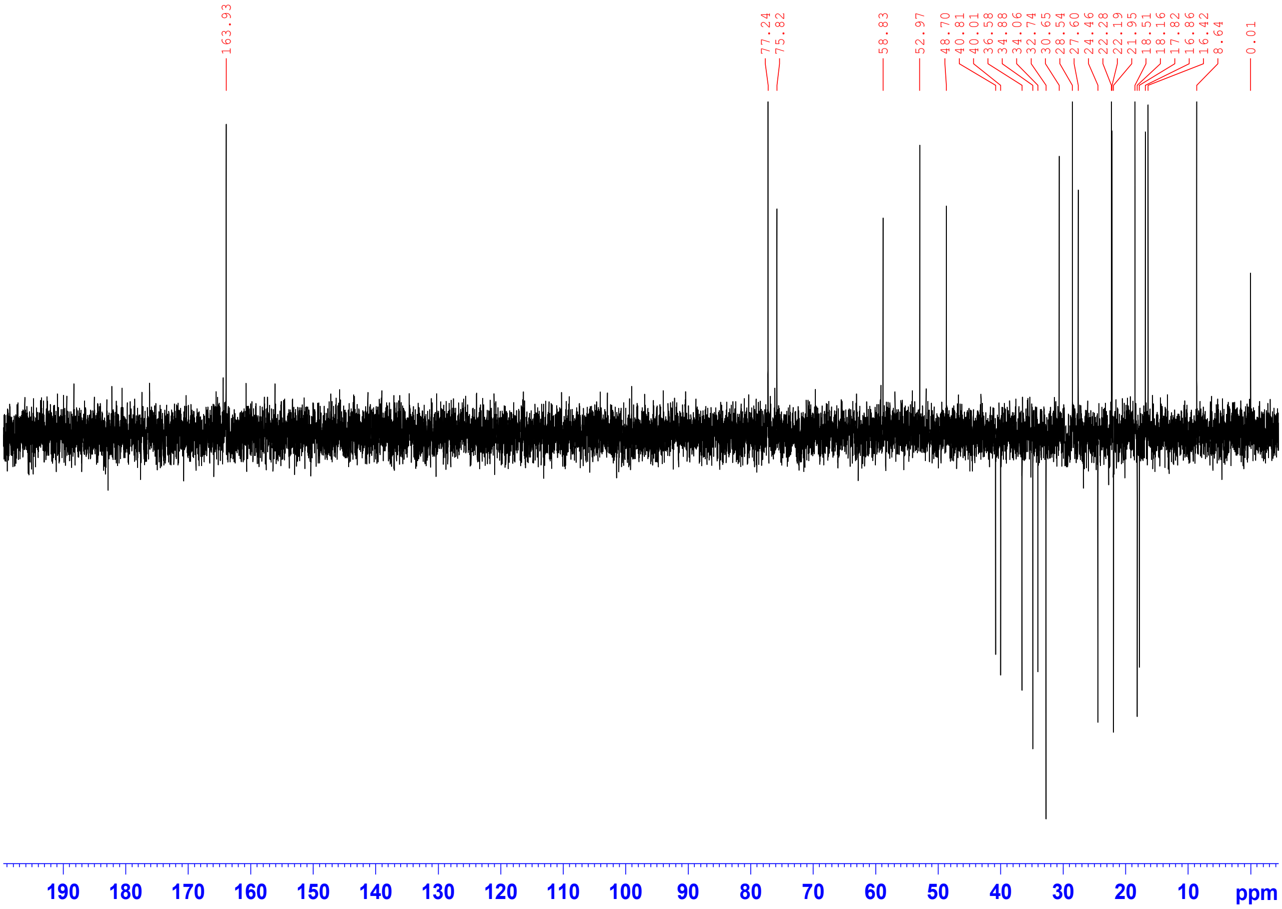

S78. HSQC spectrum of phyllofenone M (**8**) in $CDCl_3$.

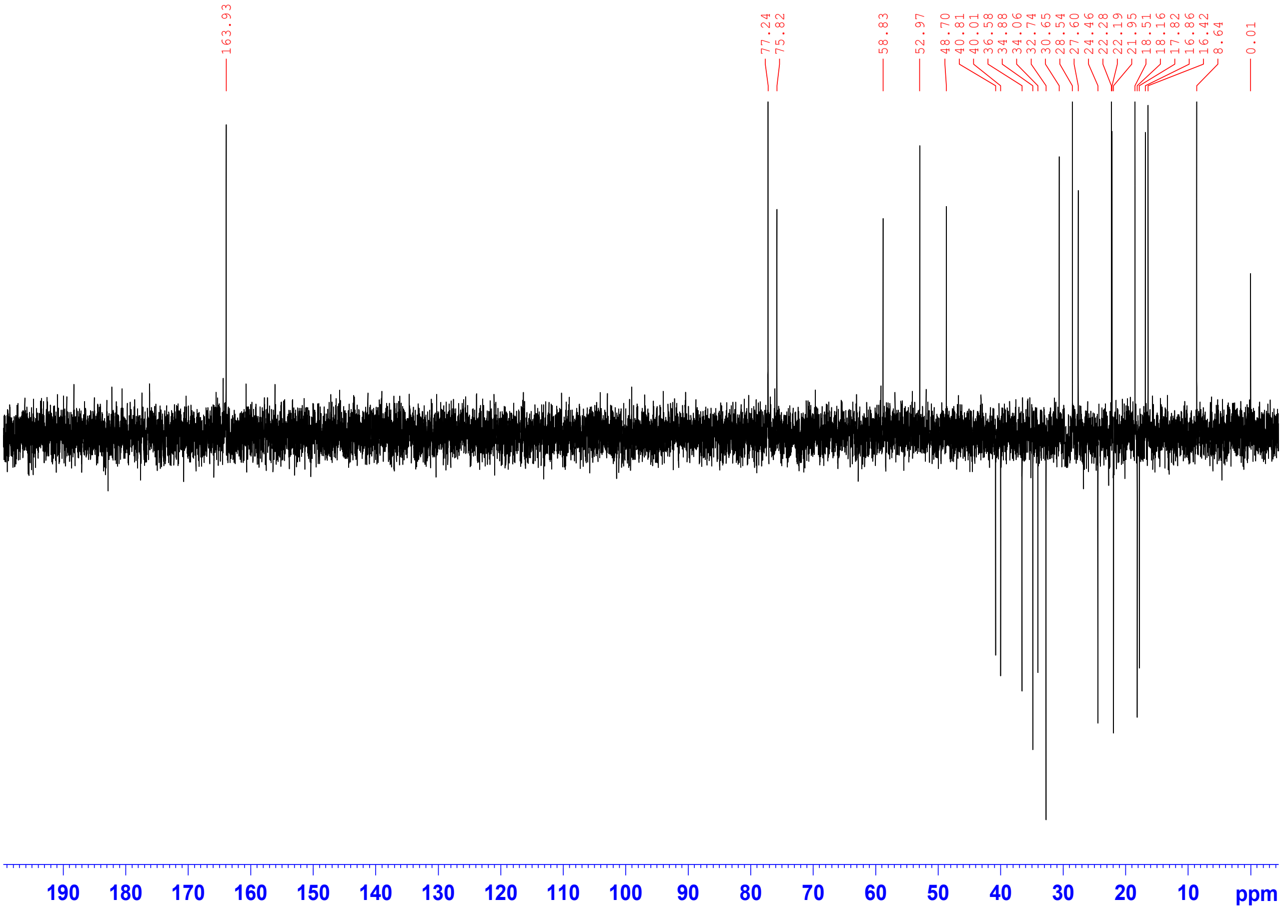

S79. COSY spectrum of phyllofenone M (**8**) in CDCl3.

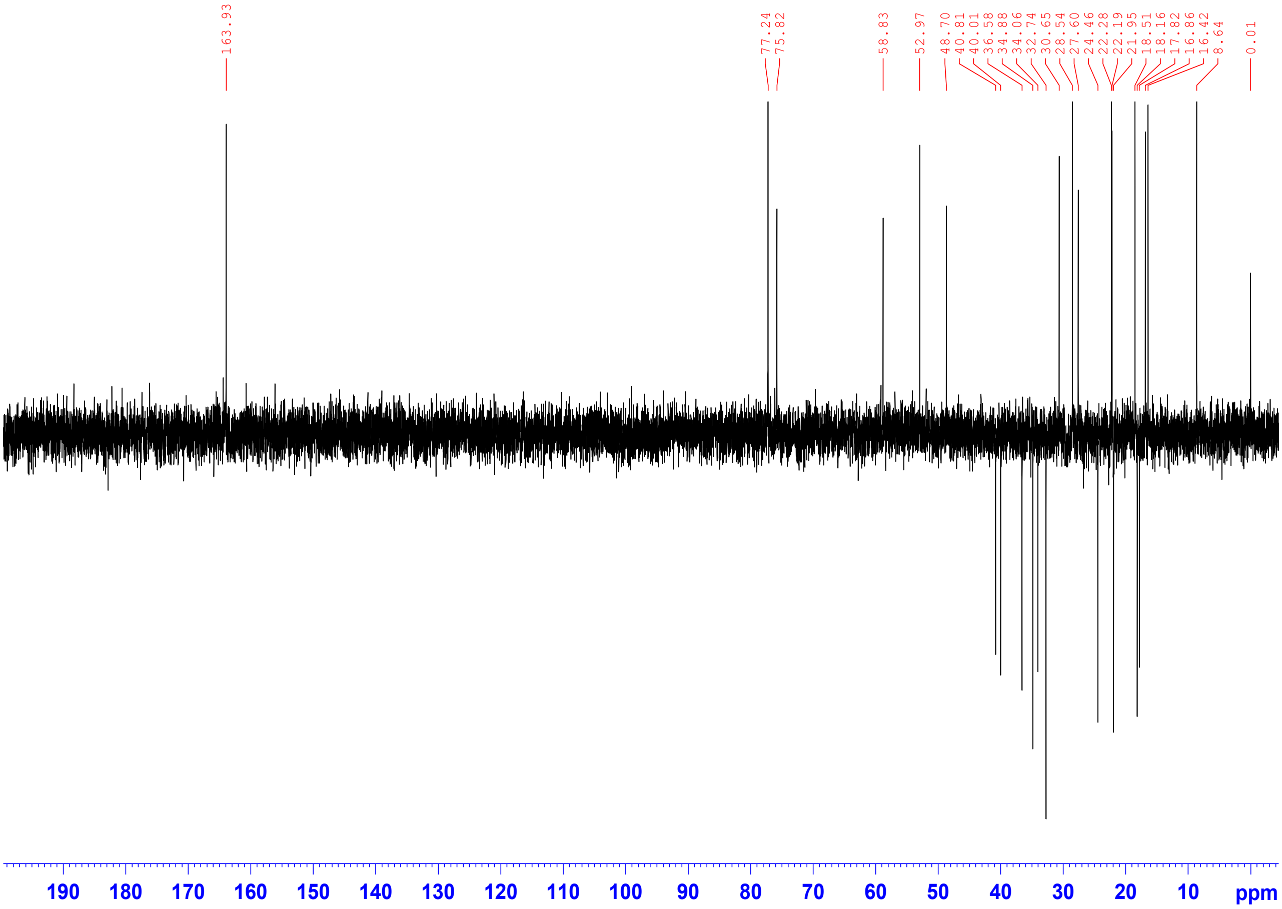

S80. HMBC spectrum of phyllofenone M (**8**) in CDCl3.

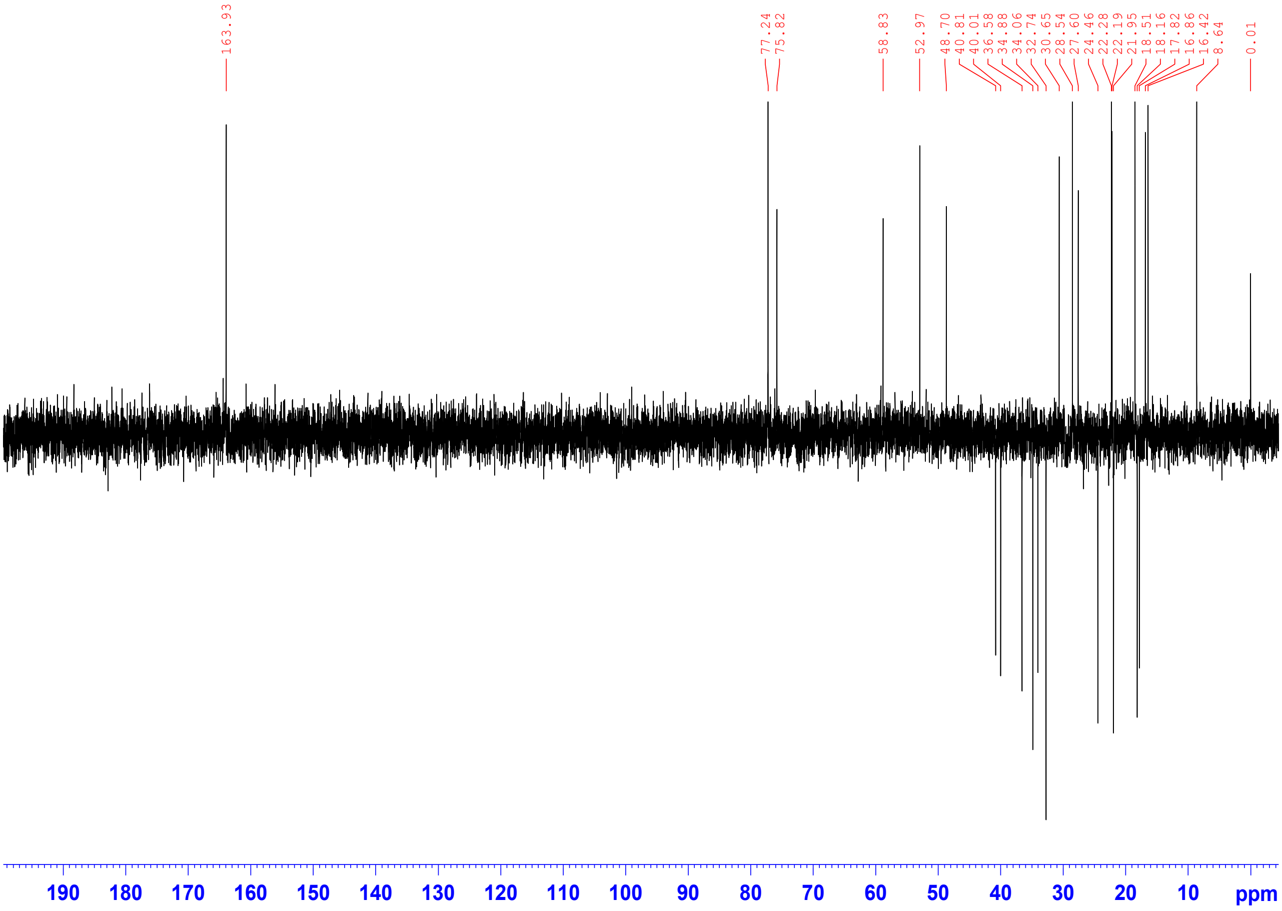

S81. NOESY spectrum of phyllofenone M (**8**) in $CDCl_3$.

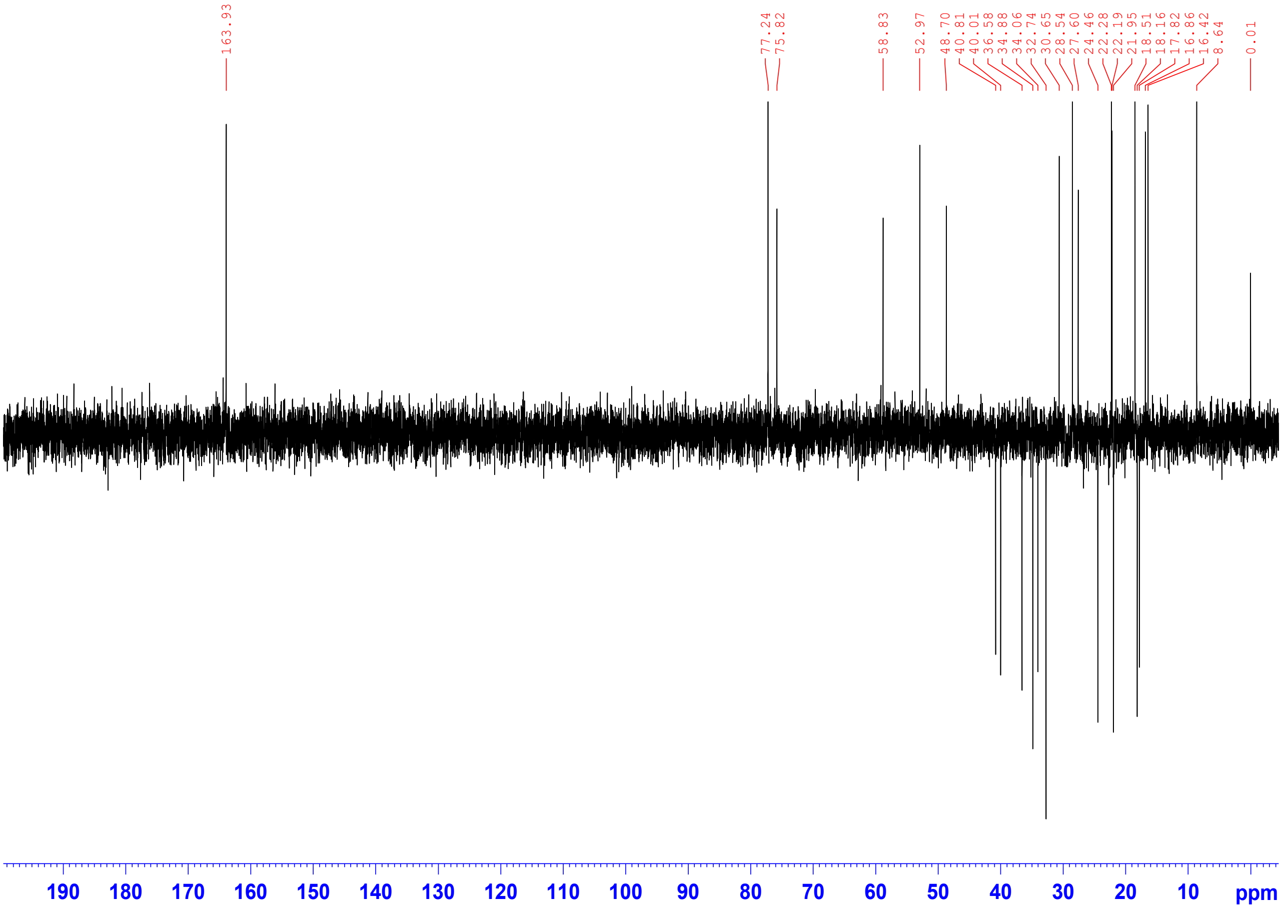

S82. HRESIMS of phyllofenone M (**8**).

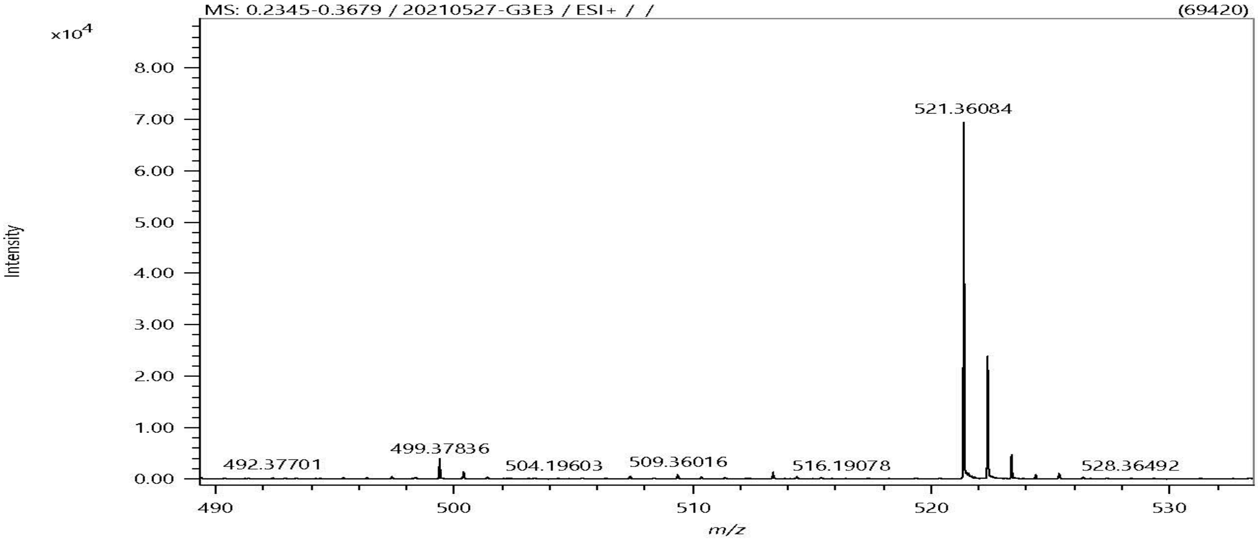

Elemental Composition

Parameters	
Tolerance:	±5.00 ppm
Electron:	Odd/Even
Charge:	+1
DBE:	-1.5 - 200.0

Elements Set 1:

Symbol	C	H	N	O	Na	S	Cl	Br
Min	0	0	0	0	1	0	0	0
Max	200	200	0	8	1	0	0	0

Symbol	F	Si
Min	0	0
Max	0	0

Results

Mass	Intensity	Intensity [%]	Formula	Calculated Mass	Mass Difference [mDa]	Mass Difference [ppm]	DBE
521.36084	69419.71	75.00	C32 H50 O4 Na	521.36013	0.71	1.37	7.5

S83. UV spectrum of phyllofenone M (**8**).

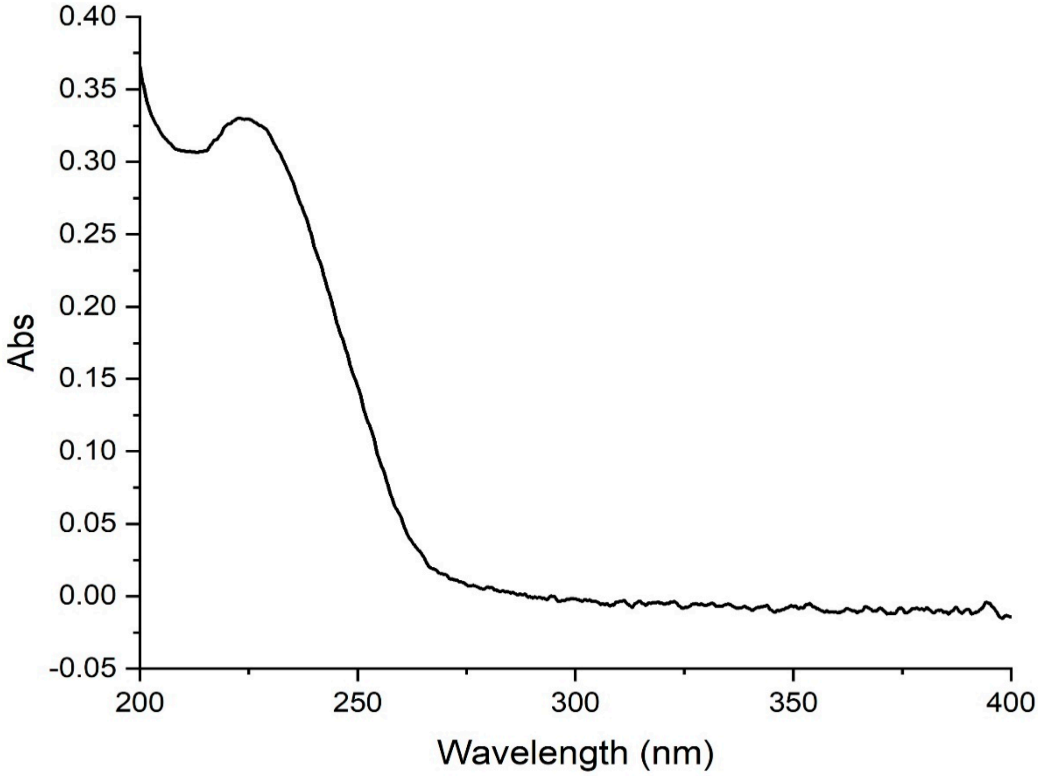

S84. IR spectrum of phyllofenone M (**8**) (KBr).

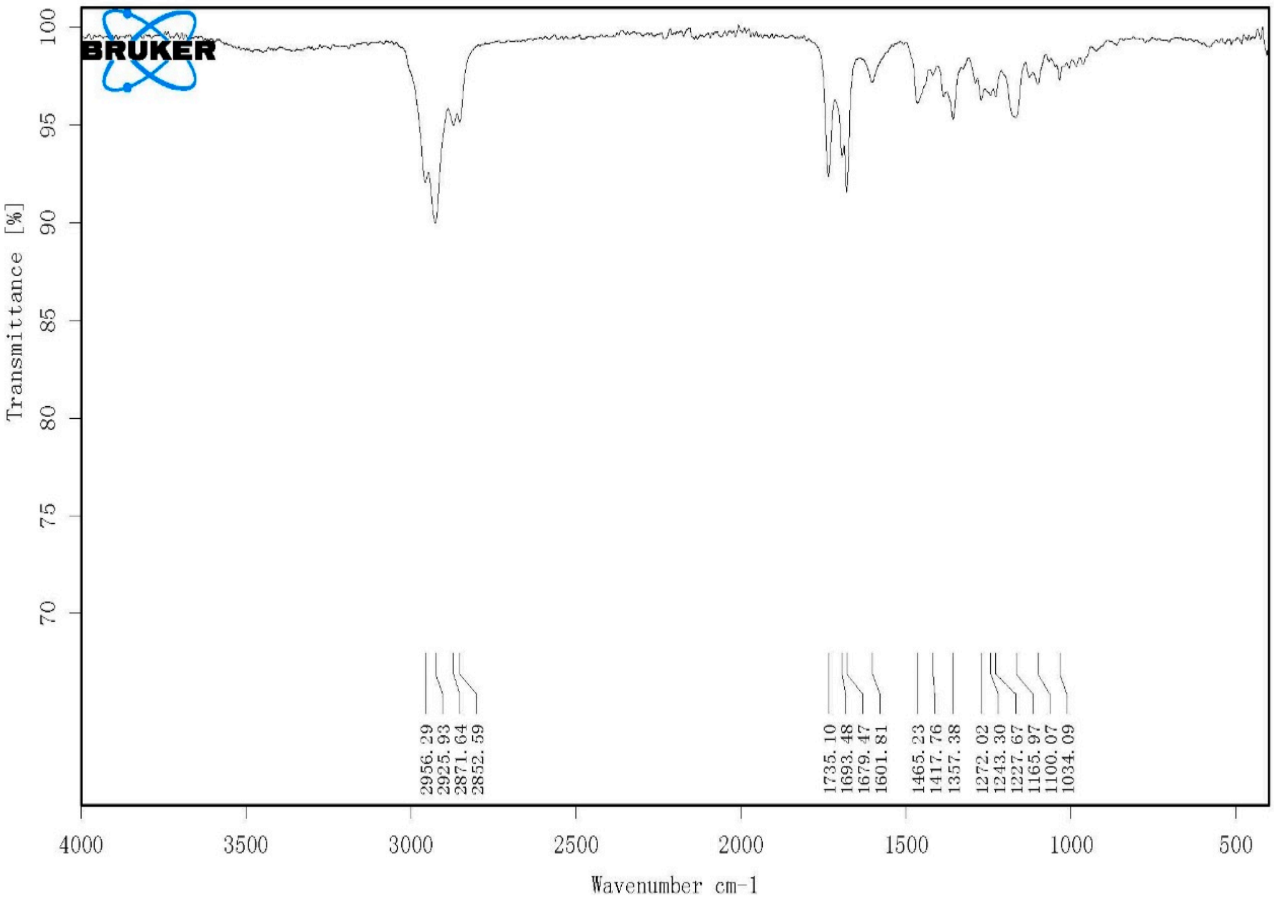

S85. The structure of phyllofenone A.

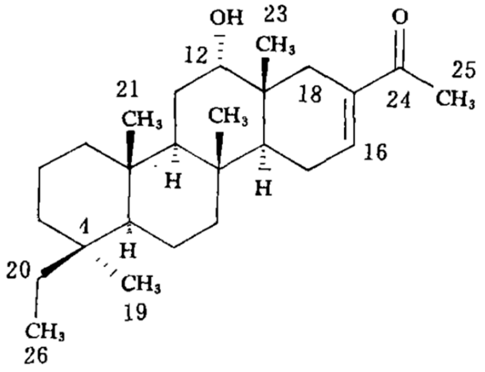

Supplement: Supplementary file 1 [file marinedrugs-21-00507-s001.zip › marinedrugs-2635829-supplementary.pdf]
